# Supramolecular Degraders: An Emerging Paradigm in Targeted Protein Degradation

**DOI:** 10.1002/advs.77305

**Published:** 2026-09-13

**Authors:** Kongjun Liu, Xue Yuan, Yan Zhou, Rui Yuan, Shaocheng Xie, Yong Chen, Peiyi Wang, Jianmei Gao, Qihai Gong

**Affiliations:** ^1^ Key Laboratory of Basic Pharmacology of Ministry of Education and Joint International Research Laboratory of Ethnomedicine of Ministry of Education Zunyi Medical University Zunyi China; ^2^ State Key Laboratory of Green Pesticide Key Laboratory of Green Pesticide and Agricultural Bioengineering Ministry of Education Center for Research and Development of Fine Chemicals of Guizhou University Guiyang China

**Keywords:** co‐assembly, degrader, self‐assembly, supramolecular chemistry, targeted protein degradation

## Abstract

Targeted protein degradation (TPD) redirects endogenous protein‐disposal pathways to selectively eliminate therapeutically relevant proteins. Over the past two decades, this field has progressed from proteolysis‐targeting chimeras (PROTACs) to an expanding repertoire of proteasomal and lysosomal degradation strategies. Recent studies indicate that supramolecular strategies are emerging in TPD, with dynamic noncovalent interactions and ordered assembly shaping degrader construction, delivery, functional integration, or intracellular assembly or activation. Here we organize recent advances into four major modes of supramolecular involvement, encompassing noncovalent modular decoupling of degrader components, delivery‐oriented single‐component self‐assembly, multicomponent co‐assembly for functional integration, and intracellular in situ assembly that generates either persistent local architectures or active degraders following cellular entry. We discuss their applications across proteasomal, endosomal‐lysosomal, and autophagy‐lysosomal degradation pathways. Finally, we discuss translational challenges and emerging opportunities for advancing supramolecular degraders, spanning modular design, strategy diversification, indication selection, systemic biodistribution, intracellular trafficking, safety assessment, and the prospective use of artificial intelligence (AI)‐assisted modeling.

## Graphical abstract

Dynamic supramolecular assembly reshapes targeted protein degradation by coordinating modular degrader construction, delivery, functional integration, and intracellular assembly or activation across proteasomal, endosomal–lysosomal, and autophagy–lysosomal pathways. A function‐oriented framework is established to clarify the roles and boundaries of supramolecular organization and to highlight emerging challenges and future opportunities for adaptable degradation systems.

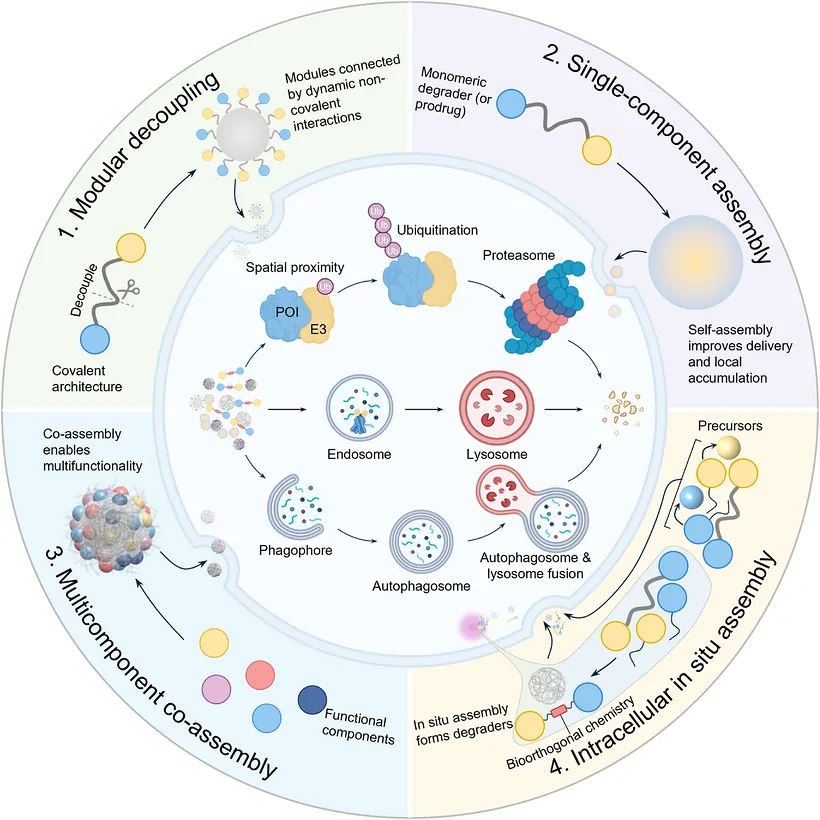

## 1 Introduction

Targeted protein degradation (TPD) has emerged as a powerful therapeutic modality that eliminates proteins of interest (POIs) by hijacking endogenous protein‐clearance machinery, expanding the druggable landscape to include disease drivers that remain recalcitrant to conventional occupancy‐driven pharmacology [1]. Rather than requiring sustained target engagement, TPD follows an event‐driven mechanism in which induced proximity between a target protein and the degradation apparatus triggers proteasomal or lysosomal disposal and results in durable functional silencing [2].

The field is now shaped by ubiquitin‐proteasome system (UPS)‐based modalities, including proteolysis‐targeting chimeras (PROTACs) and molecular glue degraders (MGDs) [3], alongside an expanding set of lysosome‐dependent strategies. These lysosome‐dependent strategies range from endocytosis‐based systems, such as lysosome‐targeting chimeras (LYTACs) and antibody‐based targeted chimeras (AbTACs), to autophagy‐based systems, such as autophagy‐targeting chimeras (AUTACs) and autophagosome‐tethering compounds (ATTECs) (Table S1) [4]. Together, these modalities have broadened the degradable space across intracellular, membrane‐associated, and extracellular proteins [5, 6].

Since the first proof‐of‐concept PROTAC was reported in 2001 [7], TPD has progressed from a chemical biology concept to a clinically engaged therapeutic modality [8], marked by the FDA approval of the ER‐targeting PROTAC vepdegestrant (ARV‐471; Veppanu) [9] and by a growing pipeline of PROTACs and MGDs in clinical development [10]. Lysosome‐dependent strategies have further diversified the field by extending degradation to extracellular proteins and to aggregation‐prone substrates that are less tractable through UPS‐based mechanisms. Despite this progress, important constraints on broader clinical translation remain unresolved. PROTACs offer modularity and mechanistic tractability, but productive ternary‐complex formation remains difficult to predict, optimization is often laborious, and hook effects together with suboptimal pharmacokinetic properties continue to complicate development [11]. MGDs present a leaner architecture, yet their discovery still resists generalizable design principles, as reflected by the fact that the best‐established clinical examples, including thalidomide and its analogues, were recognized as MGDs only retrospectively [12, 13]. Lysosome‐directed systems furnish complementary routes for protein clearance, but the expansion of target scope often brings additional challenges in delivery, intracellular trafficking, immunogenicity, and clinical translation [14], particularly when efficient target engagement depends on receptor‐mediated uptake or more elaborate functional architectures (Table S1) [15, 16, 17]. At this stage, progress is increasingly constrained by the difficulty of converting mechanistic diversity into adaptable design principles and robust strategies for in vivo deployment.

These pressures have brought supramolecular chemistry to the forefront of TPD design. Defined by dynamic noncovalent interactions, supramolecular systems offer modularity, reversibility, and multivalent cooperativity [18, 19, 20], features that map directly onto several unmet demands in degrader development. Within TPD, supramolecular organization can decouple and recombine functional modules, tune local proximity effects, improve solubility and stability, and reprogram how degraders are assembled, delivered, and activated in complex biological settings. Supramolecular chemistry thus emerges as a design principle that shapes degrader architecture, delivery logic, and biological performance across multiple levels, rather than serving merely as a formulation layer.

Although previous reviews have summarized TPD mechanisms, therapeutic progress, and nanomedicine‐enabled delivery strategies [21, 22, 23, 24, 25], the specific roles of supramolecular chemistry remain fragmented across diverse platforms and have yet to be articulated within a unified conceptual framework. In this review, we use supramolecular degraders to refer to degrader systems in which noncovalent interactions or ordered assemblies directly shape degrader design and biological performance. This definition allows recent advances to be organized according to the primary functional contribution of supramolecular organization in TPD. Accordingly, we distinguish four modes centered on degrader architecture, delivery, functional integration, and intracellular construction or activation. We first discuss how supramolecular interactions reconfigure degrader architecture by decoupling functional elements that are covalently fixed in conventional chimeras. We then examine delivery‐oriented single‐component self‐assembly and multicomponent co‐assembly for integrating targeting, responsiveness, and therapeutic synergy, followed by intracellular in situ assembly that generates either persistent supramolecular architectures capable of sustaining or spatially confining degradation activity or active degraders following cellular entry (Figure 1). Through this framework, this review aims to more clearly define the conceptual boundaries of supramolecular strategies in TPD and to discuss the translational challenges and emerging opportunities likely to shape the next phase of the field.

**FIGURE 1:**
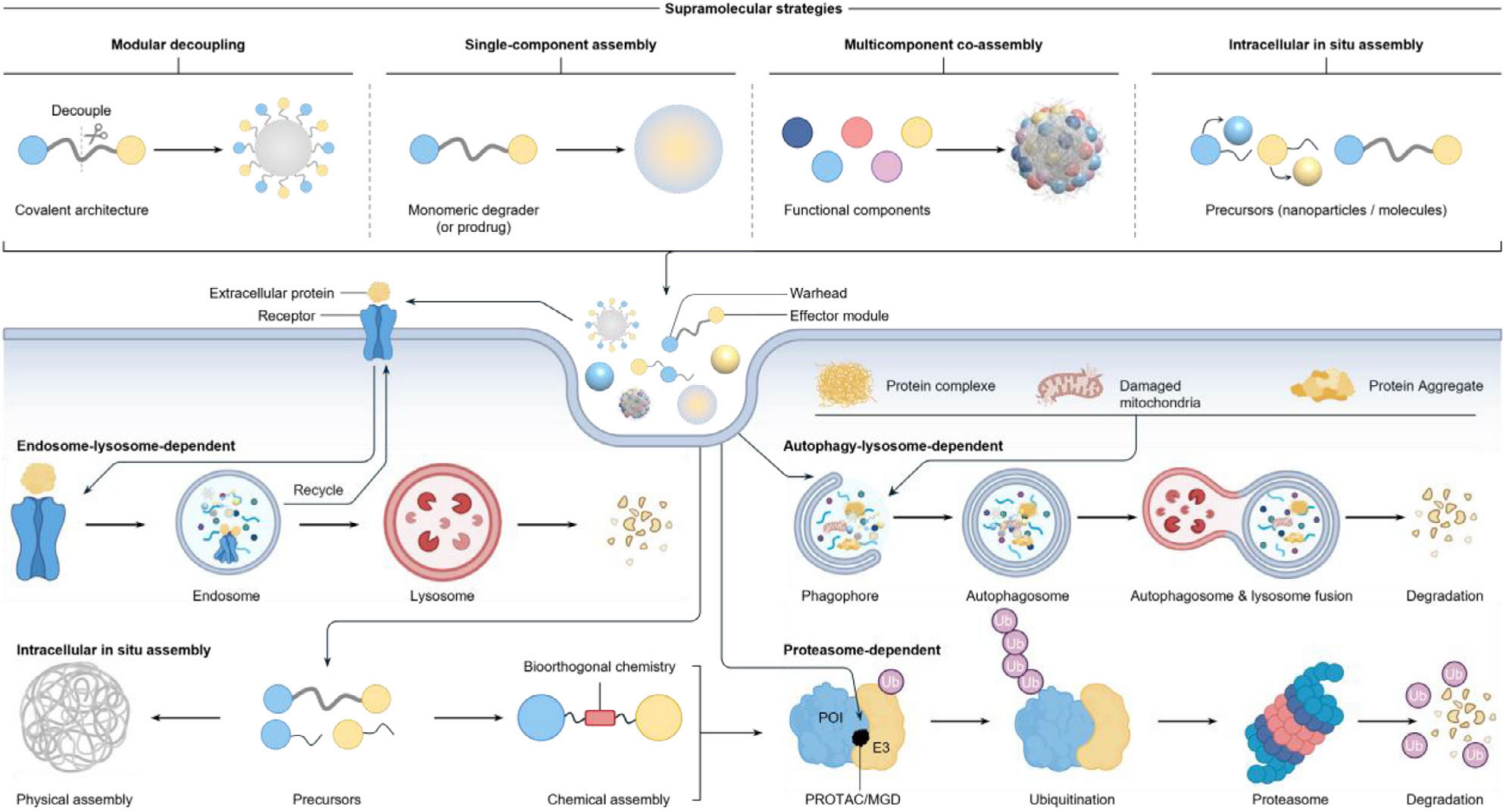
Overview of supramolecular systems in targeted protein degradation (TPD). Four forms of supramolecular involvement in targeted protein degradation are summarized, including noncovalent decoupling of warhead and effector modules, single‐component self‐assembly of covalently constructed degraders, multicomponent co‐assembly involving degraders, and intracellular in situ assembly. Degradation of target proteins or related substrates is shown through proteasomal, endosomal‐lysosomal, and autophagy‐lysosomal pathways. POI, protein of interest; E3, E3 ubiquitin ligase; Ub, ubiquitin; PROTAC, proteolysis‐targeting chimera; MGD, molecular glue degrader.

## 2 Supramolecular Decoupling of Functional Modules in TPD Design

Conventional protein degraders, including PROTACs [26] and LYTACs [27], are commonly built as fixed tripartite systems. They contain a POI‐binding warhead, an effector module that recruits degradation machinery, such as an E3 ligase or a lysosome‐targeting receptor, and a covalent linker that connects the two. Although this covalent architecture confers structural stability, it also imposes substantial design constraints. Once synthesized, the pharmacokinetic behavior, targeting profile, and degradation activity of the molecule are largely fixed. Replacing either the warhead or the effector module to satisfy distinct therapeutic demands or improve degradation efficiency generally requires laborious linker screening and extensive structural refinement. This iterative process is time‐consuming and resource‐intensive and slows TPD development [28].

Supramolecular strategies address these constraints by linking distinct functional units through dynamic noncovalent interactions. This organization decouples modules that are covalently fused in conventional degraders and enables module exchange, reversible assembly, and tunable stoichiometry. Guided by this concept, we focus here on the module‐decoupling role of supramolecular chemistry in degrader design (Tables 1 and S2). Specifically, we discuss four representative approaches, namely host–guest recognition, amphiphilic self‐assembly, oligopeptide self‐assembly, and complementary DNA recognition, and examine how each reconfigures the connection between the warhead and the effector module through a distinct assembly mode (Figure 2). Within this noncovalent decoupling paradigm, improvements in other properties, although important and noted where relevant, are not the central concern of this section. Accordingly, the design logic of each degradation platform provides the primary basis for discussion and classification. Delivery‐related and pharmacokinetic benefits are mentioned only where they help contextualize representative systems, without serving as criteria for classification, thereby distinguishing this section from those that follow.

**TABLE 1:** Summary of supramolecular platforms used for modular decoupling in TPD.

Platform name	Warhead	Effector module	Assembly motif	Assembly mode
SupTACs [31]	Ada‐conjugated POI ligands (e.g., JQ1‐Ada, GEF‐Ada)	Ada‐conjugated E3 ligase ligands (e.g., POM‐Ada)	*β*‐CD and Ada	Host‐guest recognition
HGTACs [33]	Ada‐conjugated POI ligands (e.g., Ada‐biotin, Ada‐antibody)	*β*‐CD‐conjugated lysosome‐targeting receptor ligands (e.g., *β*‐CD‐triGalNAc)	*β*‐CD and Ada	Host‐guest recognition
PLA‐based SM‐PROTAC [39]	PLA‐PEG‐POI ligand conjugates (e.g., JQ1, tamoxifen)	PLA‐PEG‐E3 ligase ligand conjugates (e.g., pomalidomide, VHL ligand)	Hydrophobic PLA segment	Hydrophobic interactions
NRs [40]	Mal‐PEG‐PLA conjugated mutant p53‐binding peptide (MBP)	Cationic lipid DOTAP (induces autophagy)	Hydrophobic PLA segment	Hydrophobic interactions
LipoSM‐PROTAC [45]	DSPE‐PEG‐POI ligand (tamoxifen)	DSPE‐PEG‐E3 ligase ligand (VHL ligand)	Hydrophobic DSPE anchor	Hydrophobic interactions
LipoSM‐PROTAC [46]	DSPE‐PEG‐POI ligands (e.g., Mitb, Cetb)	DSPE‐PEG‐E3 ligase ligand (VHL ligand)	Hydrophobic DSPE anchor	Hydrophobic interactions
LIPOTAC [48]	DSPE‐PEG‐POI ligand (lonidamine)	DSPE‐PEG‐E3 ligase ligand (thalidomide)	Hydrophobic tails of EPC and DSPE	Hydrophobic interactions
GM‐protac [49]	DSPE‐PEG‐POI ligand (gefitinib)	DSPE‐PEG‐E3 ligase ligand (CRBN ligand)	Hydrophobic DSPE anchor	Hydrophobic interactions
SM‐PROTAC [56]	δδRR‐POI ligand conjugates (e.g., Tam, Enza, JQ1)	δδRR‐E3 ligase ligand conjugates (e.g., VHL, Tha)	Aromatic rings of δδ	Diphenylglycine (δδ) recognition, π‐π stacking
Multi‐SM‐PROTAC [59]	δδRR‐POI ligand conjugates (e.g., TMXF, Pal, Enza, JQ1)	δδRR‐E3 ligase ligand conjugates (e.g., VHL, CRBN ligands)	Aromatic rings of δδ	Diphenylglycine (δδ) recognition, *π*–*π* stacking
Supra‐PROTACs [60]	Peptide‐POI ligand conjugate (e.g., Bcl‐xL ligand L1)	Peptide‐E3 ligase ligand conjugate (VHL ligand L2)	Desulfated EIYIYE hexapeptide	Bola‐amphiphilic peptide packing, *β*‐sheet formation
Supra‐LYTAC [61]	Py‐FFK‐POI ligand (e.g., biotin, BMS peptide)	Py‐FFK‐lysosome‐targeting ligand (acetazolamide)	Py‐FF backbone	Hydrophobic interactions, *π*–*π* stacking, hydrogen bonding
LYTACAs [63]	Aromatic‐modified peptide‐POI ligand (e.g., Fmoc‐17Abp)	Aromatic‐modified peptide‐receptor ligand (e.g., Fmoc‐SRAL, Fmoc‐ASGPRL)	Aromatic groups of Fmoc or Nap‐FF	Aromatic core interactions (Fmoc or Nap‐FF), *π*–*π* stacking
SM‐CMAD [66]	δδRR‐POI ligand (e.g., Tam, Enza)	δδRR‐CMA targeting motif (KFERQ)	Aromatic rings of δδ	Diphenylglycine (δδ) recognition, *π*–*π* stacking
DbTACs [71]	DNA strand 1‐POI ligand (e.g., CDK9 ligand L1)	DNA strand 2‐E3 ligase ligand (e.g., CRBN ligand L2)	DNA base sequence	Complementary base pairing
DTACs [72]	DNA strand‐POI ligand (palbociclib)	DNA strand‐E3 ligase ligand (pomalidomide)	DNA double helix	Complementary base pairing
OligoPROTACs [73]	DNA strand‐POI ligand ((+)‐JQ1)	DNA strand‐E3 ligase ligand (VH032)	DNA base sequence and toehold‐mediated strand displacement	Complementary base pairing (reversible strand displacement)
miRiaTAC [75]	Hairpin DNA 1‐POI ligand (e.g., JQ1, palbociclib)	Hairpin DNA 2‐E3 ligase ligand (pomalidomide)	DNA base sequence	Complementary base pairing (HCR)
AS‐TD2‐PRO [77]	DNA strand extended with a STAT3 decoy sequence	DNA strand‐E3 ligase ligand (VHL ligand)	DNA base sequence	Complementary base pairing
DNA Nanoinhibitor [80]	DNA nanostructure‐targeting aptamer (e.g., SL1 aptamer, H‐Apt)	Cholesterol (mediates endocytosis and lysosomal trafficking)	DNA base sequence	Complementary base pairing
LYTAC Plus hydrogel [81]	DNA Y‑motif modified with VEGFR‑binding peptide	M6P motif on DNA Y‑motif	DNA base sequence	Complementary base pairing

*Note*: The assembly motif denotes the structural unit or a molecular recognition pair that mediates supramolecular organization. The assembly mode summarizes the principal assembly or recognition process reported in the cited studies.

**FIGURE 2:**
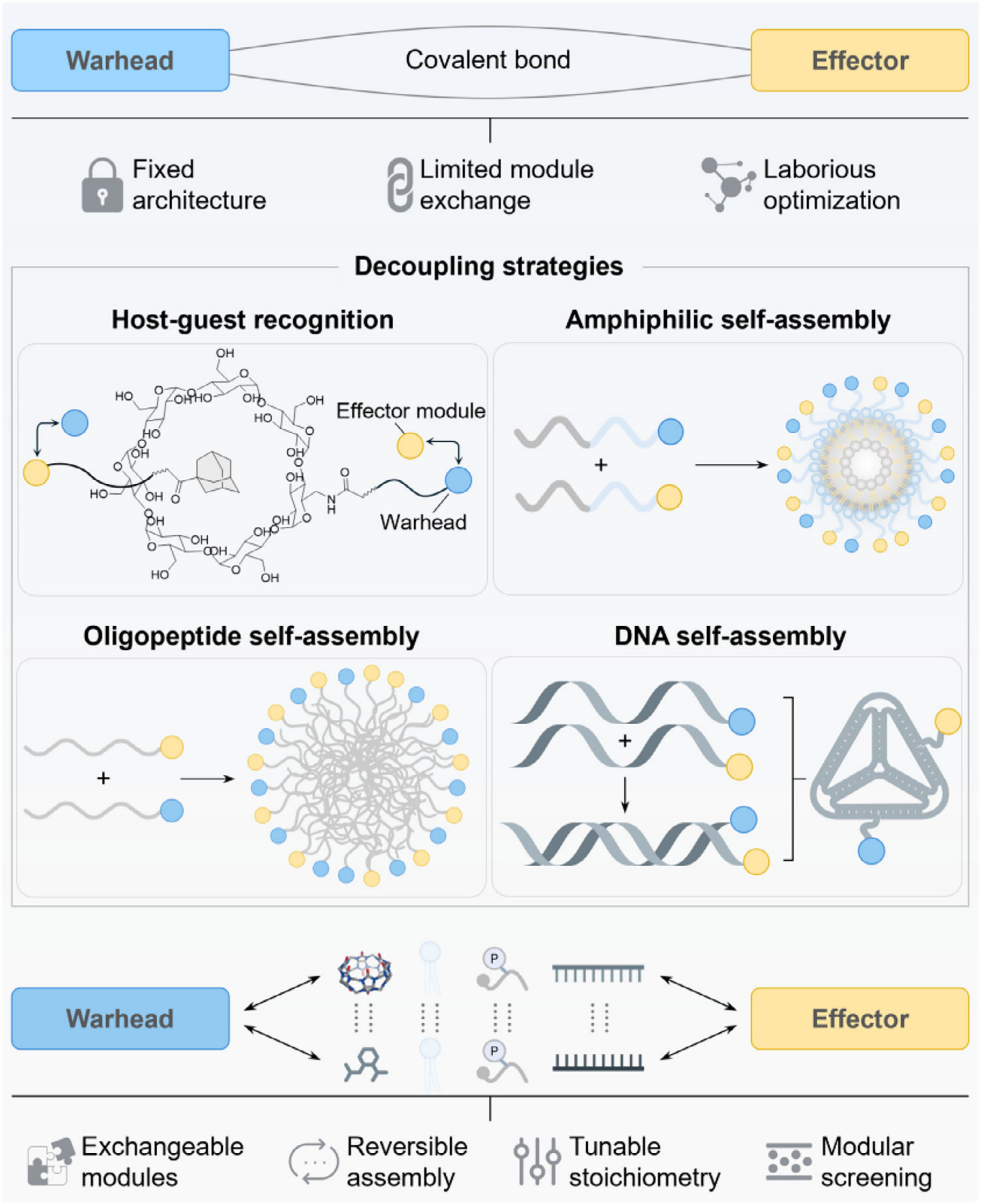
Supramolecular decoupling strategies in TPD. A covalently linked degrader contains a warhead and an effector module connected within a fixed architecture, with limited module exchange and an iterative optimization process. Supramolecular decoupling is illustrated through host–guest recognition, amphiphilic self‐assembly, oligopeptide self‐assembly, and DNA self‐assembly. These strategies connect the warhead and effector module through noncovalent interactions and generate exchangeable modules, reversible assembly, tunable stoichiometry, and modular screening.

### 2.1 Host‐Guest Recognition

Among the strategies used to construct supramolecular degraders, host–guest recognition has emerged as a representative means of achieving noncovalent modular decoupling because it combines high affinity, orthogonality, and reversibility [29]. Within this class, adamantane (Ada) and *β*‐cyclodextrin (*β*‐CD) form a classical host–guest pair that is widely used and remains stable under physiological conditions [30].

The SupTAC platform applies this recognition pair to reconfigure PROTAC design (Figure 3A) [31]. Ada‐functionalized metal‐organic cages assemble with *β*‐CD‐modified polyethyleneimine to form a nanoscale scaffold, onto which Ada‐modified E3 ligase ligands and target‐binding ligands are anchored. This architecture separates the fixed covalent PROTAC into exchangeable functional units and shifts optimization from linker redesign to modular screening and assembly‐level stoichiometric control. Adjusting the relative abundance of the warhead and effector modules optimized bromodomain‐containing protein 4 (BRD4) degradation, while warhead exchange extended the platform to epidermal growth factor receptor (EGFR), acyl‐CoA synthetase long‐chain family member 4 (ACSL4) [32], and mitogen‐activated protein kinases (MAPKs).

**FIGURE 3:**
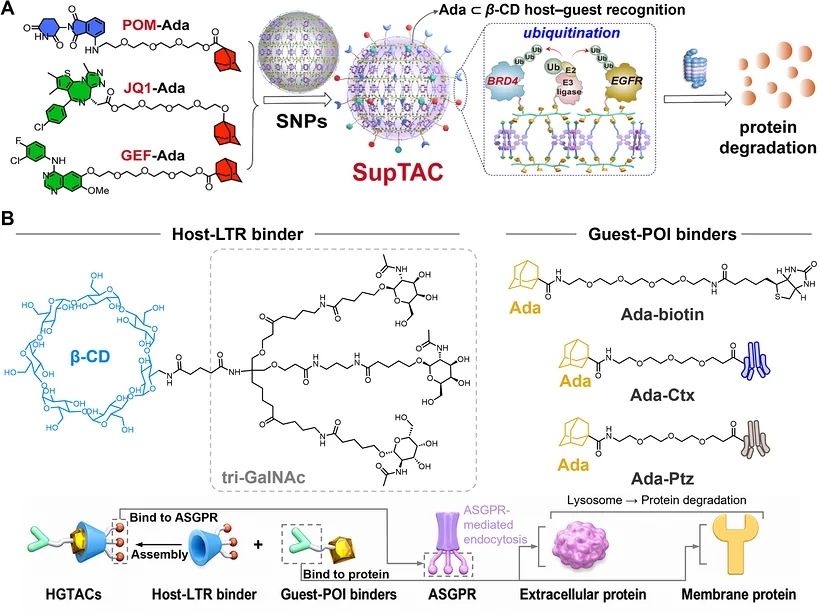
*β*‐CD/Ada host–guest recognition in supramolecular protein degradation. (A) Assembly of POM–Ada, JQ1–Ada, and GEF–Ada into SupTACs for BRD4 and EGFR degradation. Reproduced in part with permission [31]. Copyright 2025, Elsevier Inc. (B) Representative components and mechanism of HGTACs for the lysosomal degradation of extracellular or membrane proteins. Adapted with permission [33]. Copyright 2025, Wiley‐VCH GmbH.

The same recognition logic was subsequently extended from intracellular UPS degradation to receptor‐mediated lysosomal degradation in host–guest‐bridged lysosome‐targeting chimeras (HGTACs) (Figure 3B) [33]. The conventional LYTAC architecture was divided into a *β*‐CD‐conjugated tri‐GalNAc host module that targets asialoglycoprotein receptor (ASGPR) on hepatocyte surfaces [34] and an Ada‐conjugated target‐binding guest module. Their dynamic assembly enabled rapid activity screening by varying the relative proportions of the two modules. The absence of an obvious hook effect in vivo may reflect cooperation among dynamic assembly, multivalent binding, and receptor recycling [35].

Together, SupTACs and HGTACs establish host–guest recognition as a modular connection strategy across UPS and lysosome‐based degradation pathways. Its principal strengths include a well‐defined recognition mode, facile module exchange, and tunable assembly stoichiometry. Reported systems, however, remain dominated by the classical Ada and *β*‐CD pair. Given the expanding repertoire of host–guest motifs [36], future studies should determine whether alternative combinations can balance affinity, dynamic behavior, and biocompatibility, while also evaluating scaffold‐free designs for degrader construction.

### 2.2 Amphiphilic Self‐Assembly

Unlike host–guest recognition, which depends on specific pairing interactions, amphiphilic self‐assembly leverages hydrophilic‐hydrophobic balance to co‐display functional modules at a common nano‐interface, enabling noncovalent integration of warheads and effector modules. Its central design implication is interfacial colocalization, whereby otherwise distinct modules are brought into productive proximity through assembly. Accordingly, degrader optimization shifts from covalent linker architecture to the composition, stoichiometry, and spatial organization of a shared supramolecular interface.

#### 2.2.1 Polymeric Systems

Among amphiphilic building blocks, polymers have attracted broad interest because of their high structural programmability and the superior stability of the resulting assemblies [37]. Poly(ethylene glycol)‐poly(lactic acid) (PEG‐PLA), generated by covalent conjugation of poly(ethylene glycol) (PEG) and poly(lactic acid) (PLA), is a prototypical amphiphilic block copolymer widely used in biomedicine [38].

In PEG‐PLA, the hydrophobic PLA segment drives formation of the nanoparticle core, whereas the hydrophilic PEG segment forms the outer corona and enhances colloidal stability. A PLA‐based SM‐PROTAC was constructed by separately conjugating the target‐binding and E3 ligase ligands to PEG‐PLA and allowing the two polymer precursors to co‐assemble through hydrophobic interactions (Figure 4A) [39]. The ligands remain covalently distinct and become functionally coupled within the same nanoparticle, shifting degrader construction from an intramolecular linker to a shared amphiphilic interface. Within this framework, co‐assembly is mechanistically required, because simple mixing of separately preassembled particles did not reproduce degradation. The PEG‐PLA scaffold accommodated multiple ligand combinations targeting BRD4, ERα, and CDK4. Stable co‐assembly also improved in vivo durability, reduced target‐ligand consumption, and maintained activity with less frequent dosing.

**FIGURE 4:**
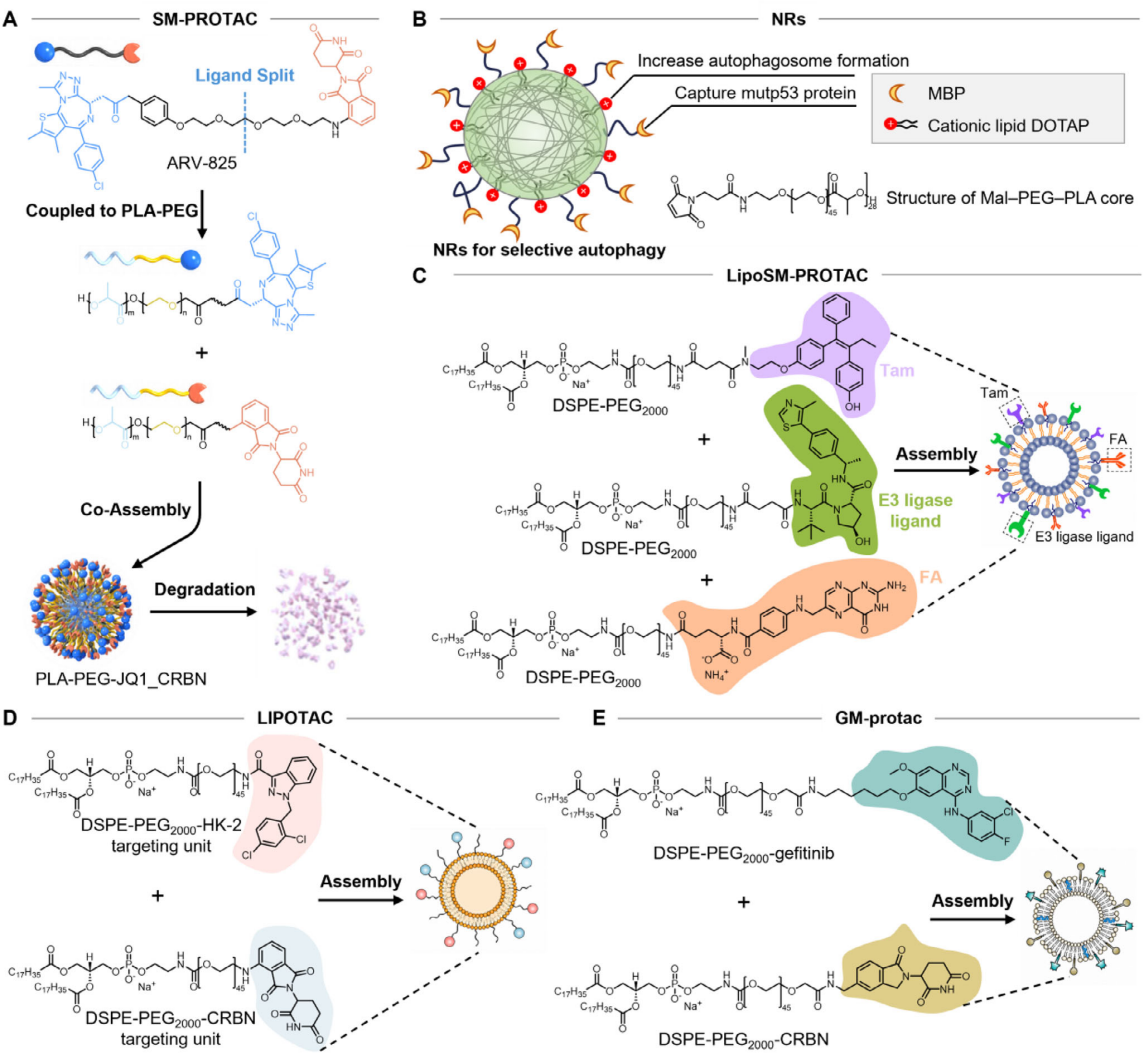
Representative amphiphilic co‐assembly platforms for modular protein degradation. (A) PLA–PEG‐based split‐and‐mix PROTACs formed by co‐assembly of separately conjugated target‐binding and E3 ligase‐recruiting modules. Adapted with permission [39]. Copyright 2025, The Author(s), published by Springer Nature. (B) Biomimetic nanoreceptors comprising MBP and DOTAP for selective autophagic degradation of mutant p53. Adapted with permission [40]. Copyright 2024, The Author(s), under exclusive licence to Springer Nature Limited. (C) LipoSM‐PROTAC assembled from DSPE–PEG_2000_ conjugates bearing ERα‐binding, VHL‐recruiting, and folate‐targeting modules. Adapted with permission [45]. Copyright 2023, American Chemical Society. (D) LIPOTAC and DOX@LIPOTAC for HK‐2 degradation and combined chemotherapy. Adapted with permission [48]. Copyright 2025, Elsevier Ltd. (E) GM‐protac assembled from EGFR‐binding and CRBN‐recruiting lipid conjugates. Adapted from Ref. [49] under the terms of the CC BY 4.0 license. Copyright 2025, The Author(s). Advanced Science published by Wiley‐VCH GmbH.

The same interfacial co‐display principle was subsequently extended to the autophagy‐lysosomal pathway through biomimetic nanoreceptors that mimic selective autophagy receptors (Figure 4B) [40]. These particles (NRs) were formed by co‐assembling maleimide‐functionalized PEG‐PLA with DOTAP, which displayed positively charged autophagy‐inducing headgroups, while surface maleimide groups enabled attachment of the mutant p53‐selective peptide MBP. In this design, MBP mediates target recognition, and DOTAP drives degradation‐pathway engagement, with both functions unified by co‐display on a single nanoparticle. Efficient mutant p53 clearance required this shared particle context, as MBP alone, DOTAP alone, or separation across different carriers failed to recapitulate degradation. The resulting nanoreceptors induced ubiquitination‐dependent selective autophagy of mutant p53 [41] and showed much weaker activity toward wild‐type p53. The two PEG‐PLA platforms therefore apply a common amphiphilic interface to distinct degradation logics, with SM‐PROTACs reconstituting E3 ligase recruitment and biomimetic nanoreceptors redirecting mutant p53 to selective autophagy.

#### 2.2.2 Phospholipid Systems

Beyond polymers, phospholipid derivatives represent another important class of amphiphilic assembly materials [42]. Upon PEGylation, phospholipids form stable amphiphilic frameworks and present terminal anchoring sites for installation of functional ligands [43]. Among these materials, distearoyl phosphatidylethanolamine‐polyethylene glycol 2000 (DSPE‐PEG_2000_) is a core component of clinically used PEGylated liposomal formulations and is widely used because it offers a membrane‐anchored amphiphile whose PEG corona can be independently functionalized before assembly [44].

The split‐and‐mix strategy was applied to DSPE‐PEG_2000_ to establish the LipoSM‐PROTAC platform (Figure 4C) [45]. The E3 ligase ligand, target‐binding ligand, and folic acid (FA) are installed on separate DSPE‐PEG_2000_ conjugates and co‐displayed on a single liposomal surface after assembly. This arrangement transfers degrader coupling from a covalently linked molecule to a compositionally tunable membrane interface, allowing degradation activity to be optimized through the identity and ratio of the surface modules. The platform achieved efficient ERα degradation and was extended to MEK and ALK by replacing the target‐binding ligand [46]. Subsequent systems expanded the scope of this DSPE‐PEG_2000_‐based design to additional targets and functions. LIPOTAC (Figure 4D) enabled degradation of hexokinase‐2 (HK‐2), which catalyzes the first step of glycolysis and represents an important metabolic target in cancer and metabolic disease [47, 48]. An EGFR‐targeting liposomal PROTAC (GM‐protac, Figure 4E) promoted UPS‐dependent EGFR degradation and cooperative clearance through the autophagy‐lysosome pathway [49]. The aqueous core and hydrophobic bilayer also permit encapsulation of companion therapeutics [50], including histone deacetylase inhibitors, without altering the surface‐level modular decoupling strategy.

Overall, amphiphilic self‐assembly based on polymers and phospholipids advances functional‐module decoupling from replaceable linkages at the molecular level to co‐display and cooperative organization at the interfacial level. Polymeric systems primarily emphasize scaffold stability and extensibility, whereas phospholipid systems additionally offer the structural advantage of combining multivalent surface presentation with internal cargo‐loading compartments. Nevertheless, current supramolecular degrader systems remain largely confined to PLA‐PEG and DSPE‐PEG_2000_ as polymeric and phospholipid scaffolds, respectively. Nanomedicine has already established a broader material repertoire, including phospholipids such as hydrogenated soy phosphatidylcholine (HSPC), distearoyl phosphatidylcholine (DSPC), dipalmitoyl phosphatidylethanolamine (DPPE), and dioleoyl phosphatidylcholine (DOPC) [51], together with amphiphilic copolymers such as poly(caprolactone)‐poly(ethylene glycol) (PCL‐PEG) and poly(lactic‐co‐glycolic acid)‐poly(ethylene glycol) (PLGA‐PEG) [52]. Expanding this repertoire should enable systematic evaluation of how scaffold composition governs assembly stability, degradation efficiency, and in vivo behavior.

### 2.3 Oligopeptide Self‐Assembly

Oligopeptide self‐assembly provides a distinct, biomimetically inspired route to modular decoupling in degrader design. Compared with amphiphilic polymeric or phospholipid systems, oligopeptides offer sequence programmability, a rich structural hierarchy, and the ability to generate diverse supramolecular morphologies through hydrogen bonding, *π*–*π* stacking, and hydrophobic interactions [53, 54]. These features allow peptide‐based assemblies to co‐display warheads and effector modules on their surfaces while also permitting in situ activation, morphology switching, and spatiotemporal selectivity through enzyme responsiveness, microenvironmental cues, or secondary‐structure engineering, providing a versatile framework for supramolecular degraders.

The diphenylglycine dipeptide Phg‐Phg, denoted δδ, self‐assembles through π‐π stacking into negatively charged nanospheres [55]. Appending two arginine residues generated δδRR, which retained self‐assembly while reversing the surface charge and improving cellular uptake. Separate conjugation of target‐binding and E3 ligase ligands to δδRR produced two monomers that co‐assembled into multivalently displayed SM‐PROTACs (Figure 5A) [56]. Adjusting their feed ratio optimized degradation, while ligand exchange extended the platform to cyclin‐dependent kinase 4/6 (CDK4/6), AR [57], BRD, mitogen‐activated protein kinase (MEK), breakpoint cluster region‐Abelson (BCR‐ABL) [58], and EGFR. The same split‐and‐mix logic was further adapted to Multi‐SM‐PROTACs [59] for programmable multitarget and multi‐effector degradation. Beyond preassembled module display, Supra‐PROTAC introduced intracellular morphology switching (Figure 5B) [60]. Its bola‐type EIYIYE hexapeptide scaffold has a strong intrinsic propensity for self‐assembly, while tyrosine sulfation generates EIYsIYsE, which forms disordered nanoparticles with the functional ligands buried in the interior. In tumor cells enriched in aryl sulfatase, enzymatic desulfation converts the nanoparticles into nanofibers, exposes the ligands on the fiber surface, and enables von Hippel–Lindau (VHL) recruitment and Bcl‐xL degradation. This morphological reconfiguration links intracellular assembly to spatially controlled degrader activation.

**FIGURE 5:**
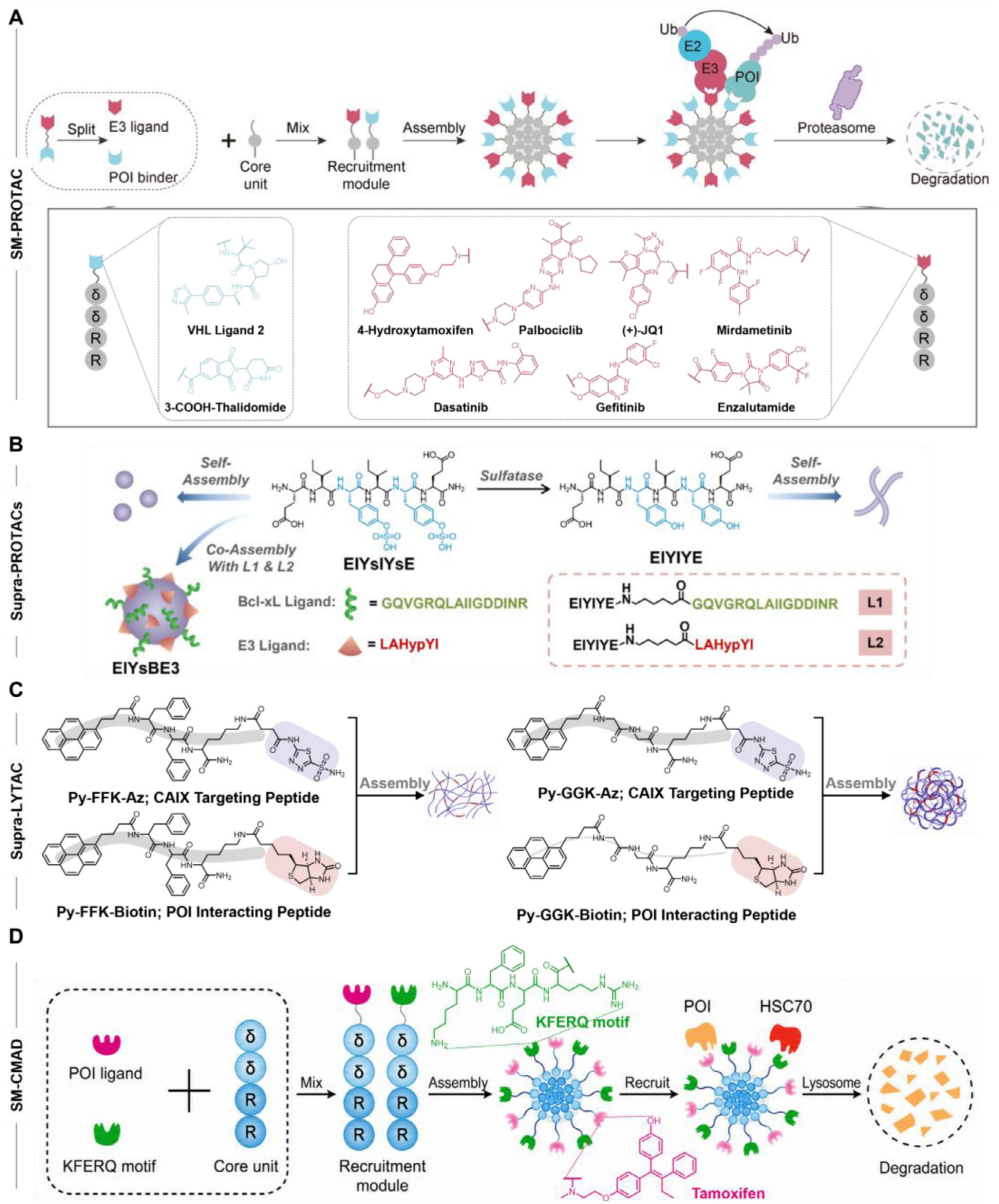
Representative peptide‐assembled supramolecular platforms for targeted protein degradation. (A) Split‐and‐mix SM‐PROTACs formed by co‐assembly of POI‐binding and E3 ligase‐recruiting modules for proteasomal degradation. Adapted with permission [56]. Copyright 2023, American Chemical Society. (B) Sulfatase‐triggered nanoparticle‐to‐nanofiber transformation of Supra‐PROTACs for Bcl‐xL degradation. Reproduced with permission [60]. Copyright 2024, American Chemical Society. (C) Peptide design and co‐assembly of Supra‐LYTACs into nanofibers or nanospheres. Adapted from Ref [61]. under the terms of the CC BY 4.0 license. Copyright 2025, The Author(s). Advanced Science published by Wiley‐VCH GmbH. (D) Split‐and‐mix SM‐CMAD platform for Hsc70‐dependent chaperone‐mediated autophagic degradation. Adapted with permission [66]. Copyright 2024, American Chemical Society.

Peptide co‐assembly was also extended to receptor‐mediated lysosomal degradation through the Supra‐LYTAC platform (Figure 5C) [61]. A CAIX‐targeting Py‐FFK effector peptide first accumulates on cancer‐cell membranes, where local enrichment recruits a target‐binding peptide that remains monomeric below its critical aggregation concentration. Their co‐assembly generates nanofibers displaying both functional modules. Because CAIX is selectively overexpressed in hypoxic solid tumors [62], this process confines assembly and degradation to the target‐cell surface. Redirecting receptor recognition to scavenger receptor A (SR‐A) enabled lysosomal degradation of IL‐17A [63], a key pro‐inflammatory factor [64], and alleviated symptoms in a psoriasis model. This extension (LYTACAs) preserved the modular assembly principle while transferring it from tumor‐associated membrane targets to inflammatory extracellular proteins. Peptide self‐assembly was subsequently redirected to chaperone‐mediated autophagy (CMA), a highly selective lysosomal degradation pathway [65]. In the SM‐CMAD platform (Figure 5D) [66], the CMA‐recognition motif KFERQ and the target‐binding ligand were conjugated to separate δδRR modules that co‐assembled into positively charged nanoparticles. Following cellular uptake, heat shock cognate protein 70 (Hsc70) [67] recognized KFERQ and mediated lysosomal delivery and target degradation. Effector substitution therefore reassigned the same supramolecular carrier from E3 ligase recruitment to chaperone‐mediated lysosomal recognition. Target‐dependent optimization of module ratios and extension to ERα, AR, MEK1/2, and BCR‐ABL further demonstrated programmable co‐assembly after pathway redirection.

Across these systems, peptide programmability supports multivalent module display, intracellular morphology switching, cell‐surface assembly, and degradation‐pathway reassignment across UPS, endosomal‐lysosomal, and CMA routes. Nevertheless, its assembly behavior remains highly sensitive to peptide sequence and microenvironment, and the associated structure‐activity relationships, together with quantitative characterization under biologically relevant conditions, remain insufficiently understood [68]. Future efforts should therefore focus on improving the predictability and controllability of these systems, while developing responsive peptide assemblies better suited to in vivo, in situ activation.

### 2.4 DNA Complementarity‐Guided Recognition

Although the strategies described above provide convenient platforms for multivalent display and stoichiometric modulation of degrader modules, control over warhead‐effector separation still depends on assembly size and ligand‐placement statistics, making atomically precise regulation difficult to achieve. Moreover, many such assemblies remain governed by thermodynamic equilibrium, dilution, or passive microenvironmental triggering, limiting deliberate external control over the timing and mode of degrader activation. DNA complementarity introduces a distinct level of programmability to supramolecular degrader design. Through predictable base pairing and structurally defined nanoscaffolds, DNA can encode the spacing, orientation, and assembly state of functional modules with a degree of precision that is difficult to attain in other supramolecular formats [69]. At the same time, its dynamic hybridization behavior enables reversible control and on‐demand switching of degrader activity [70].

DNA framework‐based PROTACs termed DbTACs (Figure 6A) established spatial configuration as an explicitly programmable variable [71]. In this system, a CDK9‐targeting ligand and an E3 ligase ligand were attached to two DNA strands and co‐assembled with two unmodified strands into a tetrahedral framework. Varying the number of nucleotides at the attachment sites tuned the interligand distance with single‐nucleotide precision. The related DTAC platform (Figure 6B) used a rigid DNA duplex to vary both interligand distance and dihedral angle [72], generating conformational libraries in which degradation potency depended on ligand geometry. The optimal arrangement enabled simultaneous degradation of the Cyclin D1, CDK4, and CDK6 protein complex. These platforms therefore convert spatial parameters that are difficult to encode in conventional PROTACs into defined and screenable design variables. DNA hybridization also enables reversible control over degrader activity. In OligoPROTACs, target‐binding and E3 ligase ligands were conjugated to complementary DNA strands that formed a rigid duplex [73]. A terminal single‐stranded toehold allowed an added complementary strand to initiate strand displacement, separate the two ligands, and switch off degradation [74]. miRiaTAC (Figure 6C) extended this dynamic control to an endogenous tumor‐associated microRNA [75]. The microRNA initiated a hybridization chain reaction between two ligand‐bearing DNA hairpins, generating elongated assemblies that brought the ligands into proximity, while a photocleavable linker (PC‐linker) enabled degradation activity to be terminated by light [76]. Together, these systems complement spatial optimization by controlling when an active degrader architecture forms.

**FIGURE 6:**
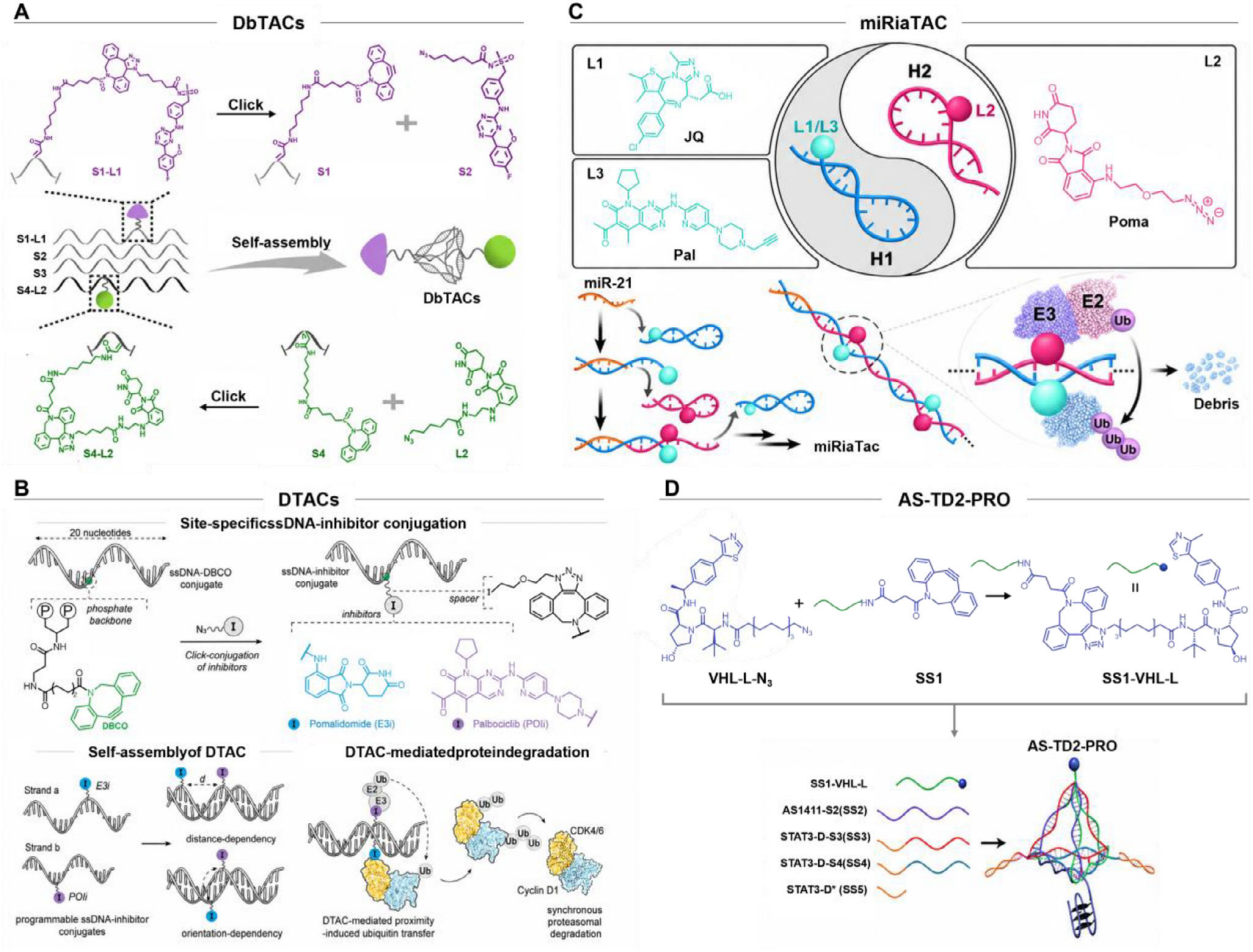
Representative DNA‐assembled platforms for targeted protein degradation. (A) Click chemistry and self‐assembly of DbTACs with programmable ligand spacing. Adapted from Ref [71]. under the terms of the CC BY 4.0 license. Copyright 2023, The Author(s). (B) DNA‐templated DTACs for geometry‐dependent degradation of the Cyclin D1–CDK4/6 complex. Adapted with permission [72]. Copyright 2025, American Chemical Society. (C) miRNA‐triggered assembly of miRiaTACs for programmable protein degradation. Adapted with permission [75]. Copyright 2024, Wiley‐VCH GmbH. (D) Assembly of AS‐TD2‐PRO integrating tumor targeting, VHL recruitment, and bivalent STAT3 recognition. Adapted with permission [77]. Copyright 2025, American Chemical Society.

DNA scaffolds can further integrate multiple functions within a single architecture. AS‐TD2‐PRO (Figure 6D) incorporated tumor‐targeting, E3 ligase‐recruiting, and bivalent signal transducer and activator of transcription 3 (STAT3)‐recognition modules at the four vertices of a DNA tetrahedron, enabling cooperative degradation of STAT3, a transcription factor that is difficult to engage with small‐molecule ligands [77, 78]. Replacement of the recognition sequence generated a dual‐target system that simultaneously degraded STAT3 and forkhead box protein M1 (FOXM1) [79]. Related architectures extended this modularity to membrane and extracellular targets. A cholesterol‐anchored DNA tetrahedron bearing exchangeable aptamer modules enabled lysosome‐mediated downregulation of either c‐Met or HER2 through aptamer substitution [80]. Although reported as a DNA nanoinhibitor rather than a canonical chimeric degrader, this platform separates membrane anchoring, target recognition, and downstream trafficking into independently tunable elements. In the nucleic acid‐based LYTAC Plus hydrogel, mannose‐6‐phosphate (M6P) motifs and vascular endothelial growth factor receptor (VEGFR)‐binding peptides provided lysosome targeting and target capture, while angiopoietin‐2 (ANG‐2)‐targeting siRNAs served as both structural cross‐linkers and gene‐silencing modules [81]. Complementary base pairing thereby integrated receptor downregulation and gene silencing within the same construct. Beyond functional integration, DNA self‐assembly can also reduce reliance on laborious empirical optimization and improve spatial control relative to conventional PROTACs [82]. Its in vivo application, however, remains constrained by nuclease degradation, low‐Mg^2^^+^ environments, and poor endosomal escape [83], often requiring chemical modification to preserve structural integrity. Hybrid systems based on DNA‐hydrophobic block copolymers [84] or DNA‐directed amphiphile assembly [85] may combine spatial programmability with improved stability and intracellular delivery.

Overall, the four strategies discussed in this section differ in their assembly‐driving forces and structural hierarchies, yet each replaces fixed covalent linkage with supramolecularly organized proximity. Host‐guest recognition provides reversible connectivity, amphiphilic self‐assembly enables interfacial co‐display and multivalent cooperativity, oligopeptides add sequence programmability, and DNA complementarity supports precise spatial control and programmable switching. Despite these differences, the four strategies share several design advantages over covalently linked degraders. First, they separate module synthesis from module combination, allowing warheads, effector ligands, targeting elements, or pathway‐directing motifs to be exchanged without redesigning a covalent linker for each module pair. This modularity can accelerate screening and facilitate adaptation to different targets or degradation pathways. Second, they shift optimization from iterative linker resynthesis to assembly‐level stoichiometric control, allowing the relative abundance of functional modules to be adjusted to tune degradation activity. Third, supramolecular organization can generate multivalent and spatially organized proximity, shifting degrader optimization from intramolecular linker‐length tuning to control over interfacial co‐display, ligand density, spatial arrangement, and scaffold‐templated positioning. Fourth, the dynamic and reversible nature of noncovalent assembly enables modes of regulation that are difficult to encode in permanently linked chimeras, including conditional activation, disassembly, morphology switching, strand displacement, and signal‐responsive assembly. Finally, reuse of the same assembly logic with different warheads or effector modules can facilitate pathway reassignment across UPS, endosomal–lysosomal, and autophagy‐related degradation mechanisms. Together, these features define a design logic complementary to covalent linkage, in which degrader performance can be optimized through modular exchange, stoichiometric tuning, spatial organization, and dynamic regulation at the assembly level.

## 3 Single‐Component Self‐Assembly Enhances Delivery and Accumulation

Supramolecular noncovalent interactions can circumvent the structural constraints of conventional architectures and free degraders from rigid design paradigms. For covalently linked degraders, however, which remain the dominant and most extensively studied class, these interactions have thus far served primarily to facilitate delivery rather than reshape degrader design. To mitigate recurrent delivery liabilities across degrader classes, including poor aqueous solubility, limited stability in circulation, and suboptimal biodistribution, investigators have explored single‐component preassembly of the degrader itself to improve in vivo delivery (Table 2 and Figure S2). Depending on whether prodrug modification is introduced, these strategies can be divided into non‐prodrug and prodrug self‐assembly, each guided by a distinct design logic for delivery optimization (Figure 7).

**TABLE 2:** Summary of single‐component self‐assembled degraders used for delivery enhancement in TPD.

Platform name	Assembly motif	Assembly mode	Key delivery gains
PT‐NPs [88]	FF dipeptide linker	Hydrophobic interactions, *π*–*π* stacking	Enhanced serum stability (48 h); improved tumor accumulation; durable PD‐L1 degradation
NanoTACs [90]	FF dipeptide linker	Hydrophobic interactions, *π*–*π* stacking	Prolonged structural integrity (7 d in serum); 3.7‐fold extended T_1/2_; 1.12‐fold higher tumor accumulation vs. Cetuximab; sustained EGFR degradation over 72 h
Nano‐LYTACs [91]	Lauryl hydrophobic tail	Hydrophobic interactions	Improved serum stability vs. free antibody; ASGPR‐mediated hepatic tumor targeting
Z_EGFR:1907_‐MS28 ADCN [95]	Hydrophobic PROTAC MS28	Hydrophobic interactions	Extended blood retention (T_1/2_ = 1.84 h); 1.9‐fold higher tumor accumulation vs. free PROTAC; enhanced biosafety at elevated doses
Z_HER2:342_‐MZ1 APCN [96]	Hydrophobic PROTAC MZ1	Hydrophobic interactions	Maintained colloidal stability (48 h in 10% FBS); prolonged circulation evidenced by slower tumor signal decay; 1.5‐fold higher tumor uptake vs. free affibody
MPRO [97]	Hydrophobic PROTAC PRO	Hydrophobic interactions, *π*–*π* stacking	High storage and serum stability (10 d at 4°C; 48 h in 10% FBS); enhanced tumor retention with reduced normal cell toxicity.
RCNprotac [99]	Hydrophobic PROTAC MZ1	Hydrophobic interactions	Improved physiological stability and PEGylated circulation; tumor signal peaked at 12 h and persisted to 24 h (vs. rapid MZ1 clearance)
Cle‐NP (nano‐mGlu) [100]	Hydrophobic molecular glue H1‐mGlu	Hydrophobic interactions	Reduced premature systemic exposure; preferential tumor accumulation; improved biosafety; more controlled in vivo release
NFTP [103]	KLVFF self‐assembling peptide	Hydrophobic interactions, *β*‐sheet formation	Stable in PBS and 10% FBS; efficient in vivo tumor targeting; minimal off‐target toxicity in normal tissues
NapYp‐ARV [105]	Dephosphorylated NapY‐peptide	Hydrophobic interactions, *π*–*π* stacking	Maintained structural integrity (24 h in serum); prolonged tumor retention (AIR effect); consistently higher intratumoral ARV‐771 levels vs. free drug
pNano‐PROTAC_GPX4_ [109]	*β*‐hairpin peptide (IHHHYI‐GPG‐IHHHYI)	Hydrogen bonding, *β*‐sheet formation, hydrophobic interactions	Hexameric assembly extended plasma T_1/2_ by 23‐fold (1.2 h→27.7 h); enhanced tumor accumulation with reduced hepatic/renal distribution
SPN_pro_ [113]	Hydrophobic PCB backbone	Hydrophobic interactions	Stable in PBS and 10% FBS; 90‐fold higher tumor signal vs. background; persistent IDO degradation
SPN_COX_ [115]	Hydrophobic PCB backbone	Hydrophobic interactions	Stable nanoparticle integrity; 58.6‐fold higher tumor fluorescence vs. background; sustained COX‐1/2 depletion
NAP [117]	Hydrophobic photosensitizer PA	Hydrophobic interactions, *π*–*π* stacking	Stable under physiological pH and serum; nanoformulation promoted tumor accumulation; reduced systemic toxicity
NPRO [118]	Hydrophobic photosensitizer PpIX	Hydrophobic interactions	High in vivo biocompatibility and minimal organ toxicity
LPN [120]	Aromatic rings of VPF	*π*–*π* stacking, hydrophobic interactions	High serum stability (48 h); prolonged circulation and enhanced PK parameters; 3.57‐fold higher tumor accumulation vs. free VPF; high drug loading (80.8%)
NanoTAC [121]	VPF moiety in drug conjugate	Hydrophobic interactions, van der Waals interactions, hydrogen bonding, *π*–*π* stacking	High serum stability (4 d); prolonged circulation and improved PK; 3.57‐fold higher tumor accumulation vs. free VPF; high drug loading (78.6%)

*Note*: The assembly motif denotes the structural unit or a molecular recognition pair that mediates supramolecular organization. The assembly mode summarizes the principal assembly or recognition process reported in the cited studies.

**FIGURE 7:**
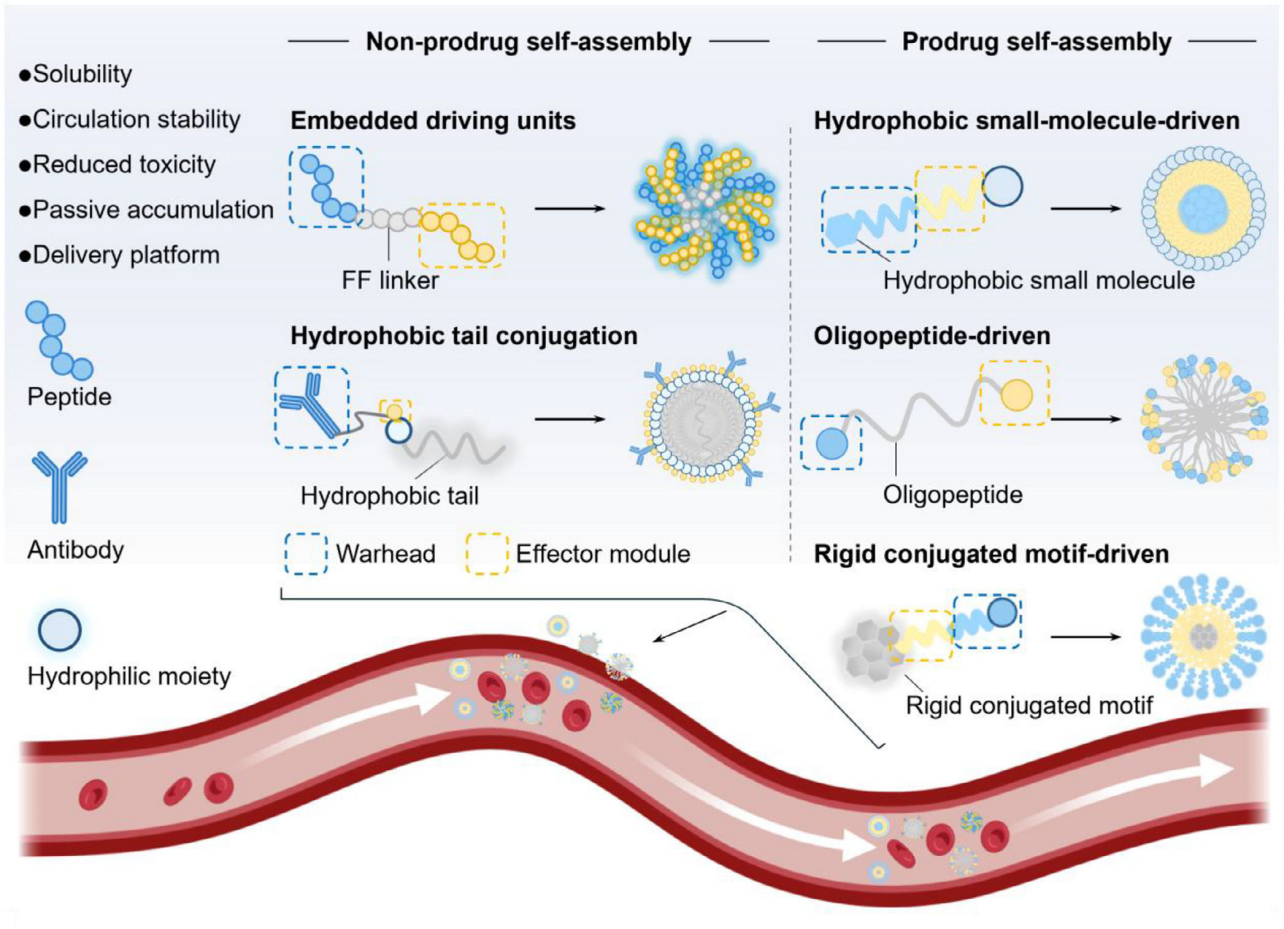
Single‐component self‐assembly of covalently constructed degraders. Single‐component self‐assembly is divided into non‐prodrug self‐assembly and prodrug self‐assembly. Non‐prodrug self‐assembly includes embedded driving units with an FF linker and hydrophobic tail conjugation. Prodrug self‐assembly includes hydrophobic small‐molecule‐driven, oligopeptide‐driven, and rigid conjugated motif‐driven assembly. FF, diphenylalanine.

### 3.1 Non‐Prodrug Self‐Assembly

In non‐prodrug self‐assembly, degraders in their active form are used directly as building blocks, with assembly‐promoting motifs introduced to drive the spontaneous formation of ordered nanoscale assemblies. Depending on how these motifs are incorporated, this strategy can be broadly divided into two modes, namely embedded driving units and hydrophobic‐tail conjugation.

#### 3.1.1 Embedded Driving Units

The diphenylalanine dipeptide (FF) is a widely used hydrophobic and π‐stacking motif in supramolecular assembly, prized for its structural simplicity and ease of incorporation [86, 87]. Leveraging the assembly propensity of FF, a peptide‐based PROTAC containing a programmed death‐ligand 1 (PD‐L1)‐targeting peptide, an FF linker, and a VHL‐recruiting peptide self‐assembled into nanoparticles termed PT‐NPs (Figure 8A) [88]. Assembly improved the aqueous dispersibility and in vivo stability of the hydrophobic peptide, while nanoscale dimensions and multivalent presentation were expected to support passive accumulation and target‐directed enrichment. Following receptor‐mediated internalization and intracellular disassembly, PT‐NPs promoted lysosomal degradation of membrane‐localized PD‐L1 and ubiquitin‐mediated degradation of cytosolic PD‐L1, limiting PD‐L1 reappearance after surface depletion, a recognized limitation of antibody‐based blockade [89]. The same FF‐driven strategy was subsequently extended to EGFR in NanoTACs (Figure 8B), which showed improved serum stability, prolonged circulation, enhanced tumor accumulation, and sustained degradation of both wild‐type and mutant EGFR [90].

**FIGURE 8:**
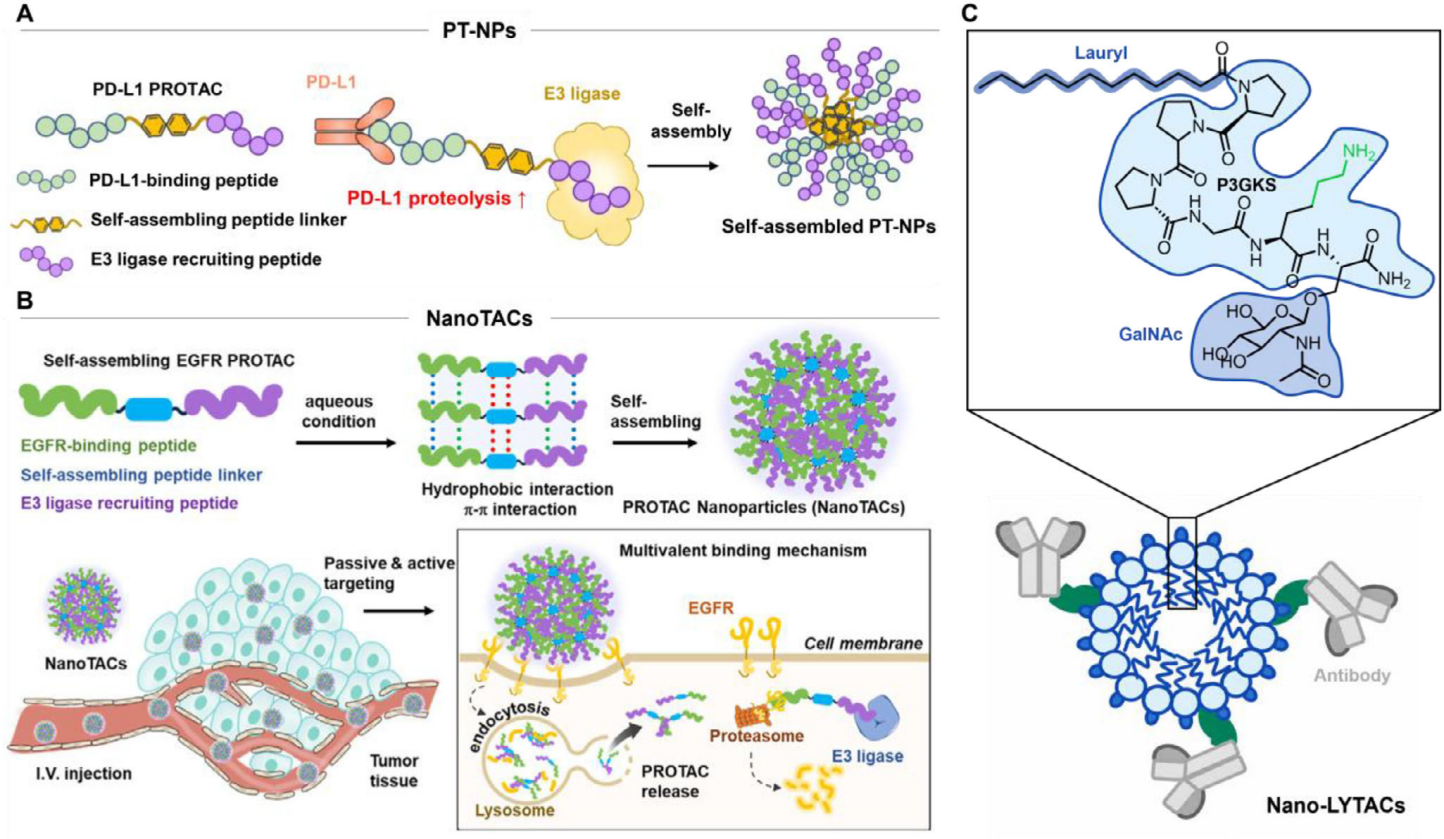
Representative self‐assembled platforms based on active degrader building blocks. (A) Self‐assembled peptide‐derived PROTAC nanoparticles (PT‐NPs) for PD‐L1 degradation. Reproduced with permission [88]. Copyright 2024, Wiley‐VCH GmbH. (B) Self‐assembled peptide‐derived PROTAC nanoparticles (NanoTACs) for EGFR degradation. Reproduced with permission [90]. Copyright 2025, Wiley‐VCH GmbH. (C) Amphiphilic glycopeptide‐based Nano‐LYTACs for ASGPR‐mediated lysosomal degradation of membrane proteins. Adapted from Ref [91]. under the terms of the CC BY 4.0 license. Copyright 2023, The Authors. Advanced Science published by Wiley‐VCH GmbH.

#### 3.1.2 Hydrophobic Tail Conjugation

Although strategies based on embedded driving units enable a high degree of functional integration, embedding also imposes functional fixation. A more extensible platform was constructed by introducing a lauroyl chain into a GalNAc‐bearing glycopeptide, generating an amphiphile that self‐assembled into nanospheres and was subsequently conjugated to antibodies through surface amino groups to form Nano‐LYTACs (Figure 8C) [91]. In this system, the antibody captures the target protein CD24, whereas multivalent surface display of GalNAc drives ASGPR‐mediated endocytosis and directs the target to lysosomes for degradation. Assembling the scaffold before antibody conjugation shifts LYTAC construction from case‐specific synthesis toward a post‐functionalizable platform for degrading extracellular and membrane proteins. The nanosphere scaffold also improved the in vivo stability of the conjugated antibody relative to its free counterpart. Although the hydrophobic core of the nanoparticle can encapsulate glucose oxidase (GOx) for starvation therapy [92], GOx remains a loaded cargo and does not participate in supramolecular organization.

These delivery gains are accompanied by unresolved liabilities arising from the constitutive activity and dynamic nature of non‐prodrug assemblies. The relationship between target‐site disassembly, active‐species release, and degradation efficiency remains poorly defined. Because degrader activity is retained during circulation, the in vivo fate of these assemblies must be evaluated against the competing requirements of supramolecular reversibility and structural stability. Under blood‐dilution conditions, for example, assemblies sustained by noncovalent interactions may undergo uncontrolled disassembly [93], leading to premature exposure of active degraders, compromised targeted delivery, and an increased risk of systemic toxicity.

### 3.2 Prodrug Self‐Assembly

The constitutive activity of non‐prodrug degraders limits the protection achievable through physical shielding alone, motivating the incorporation of chemical masking into self‐assembled platforms. Relative to non‐prodrug systems, prodrug self‐assembly combines delivery enhancement with reversible covalent suppression of degrader activity, reducing nonspecific interactions and systemic exposure through complementary chemical and physical protection. Based on the dominant source of the assembly‐driving force, these strategies are grouped into three classes, namely hydrophobic small‐molecule‐driven, oligopeptide‐driven, and rigid conjugated motif‐driven systems.

#### 3.2.1 Hydrophobic Small‐Molecule‐Driven Assembly

Hydrophobic small‐molecule‐driven assembly repurposes the intrinsic hydrophobicity of degraders as a driving force for nanoscale organization. This design logic has been developed extensively in PROTAC systems, which are often highly hydrophobic, a property that compromises druggability while supporting self‐assembly [94]. Uncontrolled hydrophobic aggregation, however, typically produces disordered and unstable structures that are unsuitable for delivery. The central challenge is therefore to direct this intrinsic hydrophobicity toward defined nanoarchitectures with improved circulation, biodistribution, and target‐site enrichment.

To this end, a hydrophilic EGFR‐targeting affibody was linked to a hydrophobic PROTAC via a disulfide bond, generating an amphiphilic conjugate that self‐assembled into nanoparticles (Z_EGFR:1907_‐MS28 ADCN) under the driving force of the intrinsic hydrophobicity of the PROTAC (Figure 9A) [95]. The affibody corona conferred colloidal stability and prolonged blood retention, while EGFR‐mediated uptake and nanoscale transport enhanced tumor accumulation and PROTAC distribution. The same design was extended to HER2‐targeting affibody‐PROTAC nanoparticles (Z_HER2:342_‐MZ1 APCN) [96], which retained receptor‐mediated uptake and showed prolonged circulation and greater tumor deposition than the free affibody. In both systems, intracellular GSH cleaved the disulfide linkage and released the active PROTAC. A related ligand‐based design used PEGylated FA as the hydrophilic segment to generate MPRO nanomicelles (Figure 9B) [97]. In this construct, the PEG corona improved aqueous solubility and storage and serum stability, FA mediated active targeting [98], and intracellular GSH cleaved the disulfide bond to release the PROTAC, increasing tumor retention and reducing toxicity toward normal cells. A PEG‐PROTAC nanomicelle (RCNprotac, Figure 9C) containing a diselenide linker extended this design from endogenous reductive activation to X‐ray‐triggered release [99]. The assembly remained largely inactive and stable during circulation, accumulated in tumors, and released the active PROTAC upon irradiation.

**FIGURE 9:**
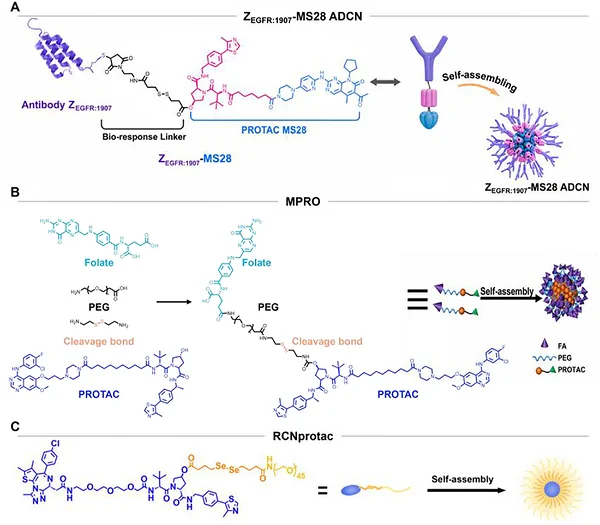
Representative degrader nanoassemblies driven by hydrophobic small molecules. (A) Amphiphilic affibody–PROTAC conjugate and its self‐assembled nanoagent, Z_EGFR:1907_–MS28 ADCN. Adapted with permission [95]. Copyright 2024, Elsevier B.V. (B) FA–PEG–PROTAC conjugate and self‐assembled MPRO nanomicelles. Adapted with permission [97]. Copyright 2024, Wiley‐VCH GmbH. (C) Self‐assembly of the X‐ray‐responsive nanomicelle RCNprotac. Reproduced from Ref [99]. with permission from the Royal Society of Chemistry.

Hydrophobic small‐molecule‐driven self‐assembly has also been extended to MGDs. The amphiphilic nano‐mGlu system Cle‐NP was generated by installing a hydrophilic mPEG segment and a GSH/H_2_O_2_‐cleavable linker on a Bcr‐Abl‐targeting molecular glue [100]. Self‐assembly reduced premature systemic exposure and the widespread toxicity of the parent fumarate‐based degrader, while improving tumor accumulation and release control. Cle‐NP therefore extends this assembly strategy from bifunctional PROTACs to monovalent MGDs.

Collectively, these studies show that intrinsic degrader hydrophobicity can support the formulation of both PROTACs and MGDs as controllable nanoassemblies. Targeting and stimulus responsiveness remain encoded in the molecular design, whereas self‐assembly primarily improves their delivery behavior in vivo.

#### 3.2.2 Oligopeptide‐Driven Assembly

Beyond exploiting the intrinsic hydrophobicity of degraders, another important strategy for prodrug self‐assembly involves the incorporation of oligopeptide motifs with inherent self‐assembling propensity as assembly‐driving modules [101]. An amyloid‐inspired prodrug termed NFTP integrated the self‐assembling KLVFF motif [102], an integrin α6‐targeting ligand, a cathepsin B (CatB)‐cleavable linker, and a FOXM1‐targeting PROTAC within a single peptide scaffold [103]. KLVFF‐driven *β*‐sheet formation produced nanoparticles that remained stable in PBS and FBS and underwent integrin α6‐mediated uptake, with in vivo imaging showing progressive tumor accumulation and low systemic toxicity. Following internalization, the CatB‐cleavable linker released the active PROTAC and induced degradation of FOXM1, a regulator of cell‐cycle progression, DNA damage repair, stem‐cell maintenance, and tumorigenesis [104].

Building on this static assembly, NapYp‐ARV introduced enzyme‐triggered morphology switching to prolong intratumoral retention (Figure 10A) [105]. The phosphotyrosine‐containing NapYp scaffold linked to ARV‐771 formed nanoparticles under physiological conditions, supporting circulatory stability and limiting premature leakage. Cell‐surface alkaline phosphatase overexpressed in tumors hydrolyzed the phosphate group, perturbed the amphiphilic balance, and induced an in situ transition from nanoparticles to nanofibers. The resulting assembly and aggregation induced a retention effect that prolonged local drug availability [106, 107, 108] and produced higher and more persistent intratumoral ARV‐771 exposure than the free drug while reducing distribution to off‐target organs. After internalization, carboxylesterase released the active PROTAC and induced BRD4 degradation.

**FIGURE 10:**
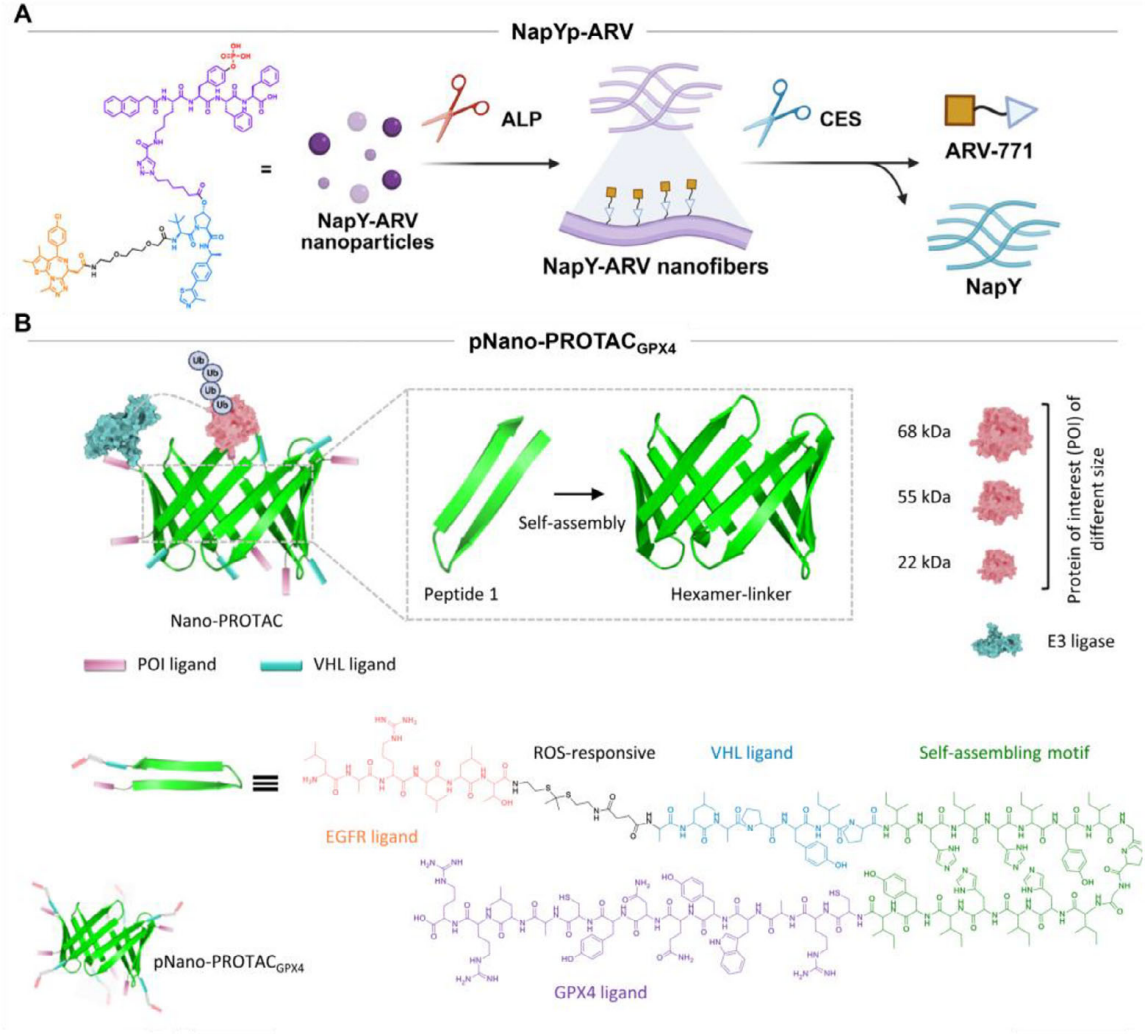
Representative peptide‐motif‐driven prodrug nanoassemblies for targeted protein degradation. (A) Enzyme‐responsive morphological transformation of NapYp‐ARV and subsequent PROTAC release. Reproduced with permission [105]. Copyright 2026, Wiley‐VCH GmbH. (B) Hexamer‐linker‐based Nano‐PROTAC design and the dual‐gated pNano‐PROTAC_GPX4_ system for tumor‐selective GPX4 degradation. Reproduced with permission [109]. Copyright 2025, Wiley‐VCH GmbH.

A distinct peptide design used supramolecular preorganization to optimize pharmacokinetics and target‐engagement geometry. Hydrogen bonding and hydrophobic interactions within a designed *β*‐hairpin scaffold generated a size‐defined, rigid, *β*‐barrel‐shaped hexamer (pNano‐PROTAC_GPX4_, Figure 10B) [109]. Its well‐defined cavity and surface supported high‐density, multidirectional ligand presentation and accommodated the spacing requirements between target proteins and E3 ligases of different molecular weights, establishing a geometry‐matching strategy for glutathione peroxidase 4 (GPX4) degradation [110]. Compared with linear‐linker counterparts, the hexamer improved plasma stability and in vivo distribution. Incorporation of an EGFR‐targeting peptide and a reactive oxygen species‐responsive bond further enhanced tumor‐selective accumulation and reduced off‐target exposure before intracellular activation of GPX4 degradation.

#### 3.2.3 Rigid Conjugated Motif‐Driven Assembly

Rigid conjugated motifs, owing to their strong *π*–*π* stacking and hydrophobic interactions, can drive the spontaneous assembly of prodrug molecules into stable nanostructures in aqueous media and thus constitute another important class of assembly‐driving modules for prodrug self‐assembly [111]. Among these motifs, poly(cyclopentadithiophene‐alt‐benzothiadiazole) (PCB) is of particular interest because of its favorable photophysical properties and chemical tunability [112]. These properties were exploited in a PCB‐based platform in which hydrophilic PEG and an IDO‐targeting PROTAC were installed on the PCB scaffold, with the PROTAC connected through a CatB‐cleavable peptide linker. The resulting amphiphilic conjugate self‐assembled into SPN_pro_ nanoparticles (Figure 11A) [113], which showed colloidal stability and passive tumor accumulation, followed by CatB‐mediated activation of the PROTAC [114]. Replacing the IDO‐targeting module with a cyclooxygenase‐1/2 (COX‐1/2)‐targeting PROTAC generated SPN_COX_ (Figure 11B) [115], which retained the same assembly and biomarker‐responsive activation logic.

**FIGURE 11:**
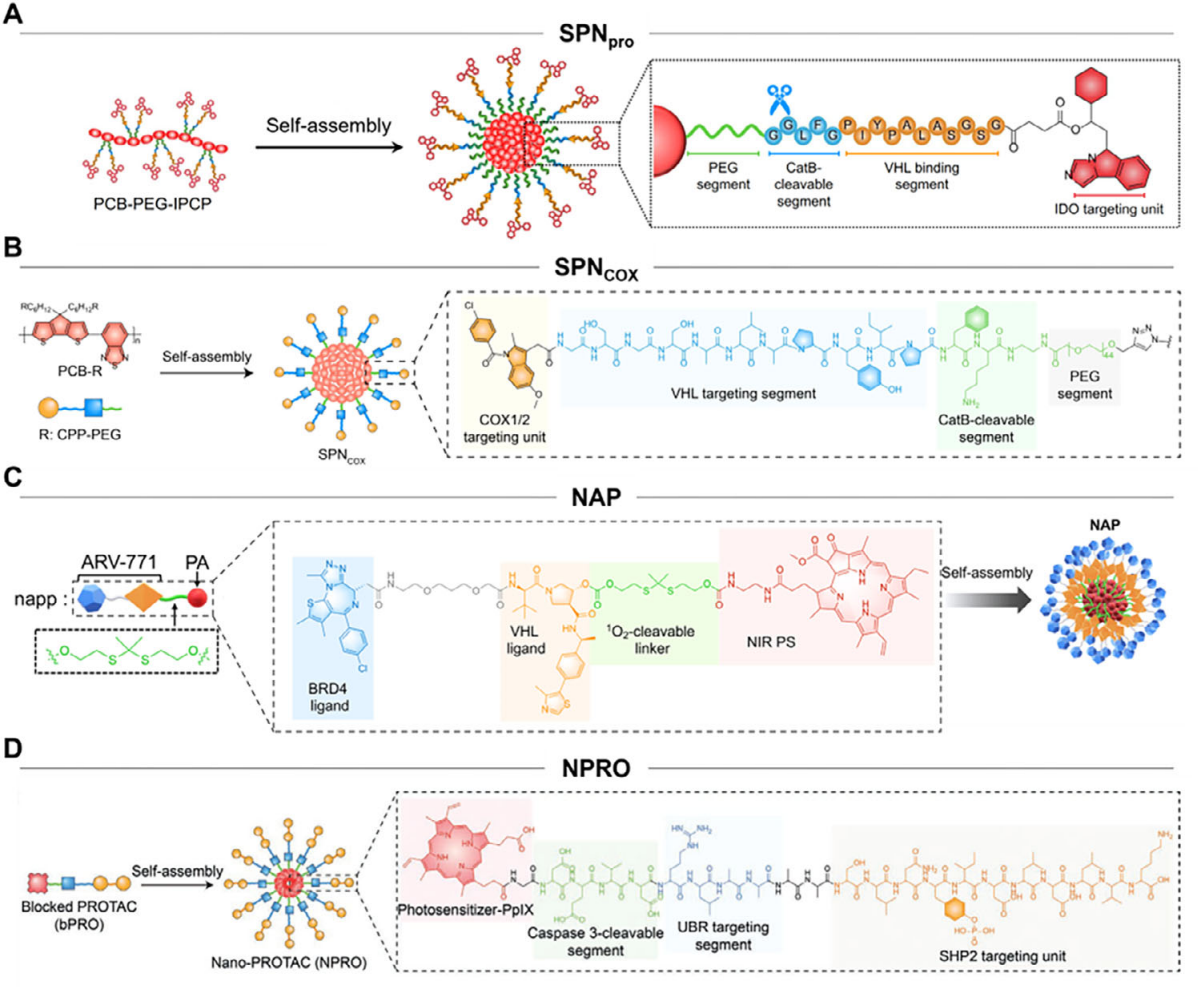
Representative rigid conjugated motif‐driven prodrug nanoassemblies for targeted protein degradation. (A) SPN_pro_, a PCB‐based nanoassembly for CatB‐activated IDO degradation. Adapted from Ref [113]. under the terms of the CC BY 4.0 license. Copyright 2021, The Author(s), published by Springer Nature. (B) SPN_COX_, a PCB‐based nanoassembly for CatB‐activated COX‐1/2 degradation. Reproduced with permission [115]. Copyright 2021, Wiley‐VCH GmbH. (C) NAP, a near‐infrared‐responsive nanoassembly for controlled activation of a BRD4‐targeting PROTAC. Reproduced with permission [117]. Copyright 2023, American Chemical Society. (D) NPRO, a photosensitizer‐driven nanoassembly for caspase‐3‐activated SHP2 degradation. Reproduced with permission [118]. Copyright 2022, Wiley‐VCH GmbH.

Related designs replaced the polymeric PCB scaffold with small‐molecule photosensitizers, which combine structural simplicity, defined functionality, and strong *π*–*π*‐stacking propensity [116]. In NAP, pyropheophorbide a (PPa) was linked to a PROTAC through a singlet‐oxygen‐cleavable thioketal linker, and the hydrophobicity and *π*–*π* stacking of PPa drove nanoparticle formation (Figure 11C) [117]. Masking of the VHL‐binding hydroxyl silenced degrader activity until near‐infrared irradiation cleaved the linker and released the active PROTAC. A more tightly coupled intracellular cascade was achieved in NPRO (Figure 11D) [118]. Protoporphyrin IX (PpIX) was linked to a caspase‐3‐cleavable substrate, an SHP2‐targeting peptide, and an E3 ligase‐recruiting sequence to form the amphiphilic precursor bPRO. PpIX served as both the assembly‐driving and photodynamic module. Light‐induced apoptosis activated caspase‐3, which cleaved the substrate and enabled SHP2 degradation [119]. Compared with systems governed solely by an external trigger, this design introduced an additional intracellular processing step after tumor accumulation, tightening spatial control over PROTAC activation. NAP therefore relied on direct photochemical uncaging, whereas NPRO introduced apoptosis‐dependent intracellular processing to strengthen spatial control over degrader activation.

In multimodal combination therapy, a central challenge is to ensure the synchronous release of multiple functional modules within the same spatiotemporal window. One reported strategy addressed this challenge by linking the photosensitizer verteporfin (VPF) to an IDO‐targeting PROTAC through a CatB‐cleavable peptide segment [120]. The hydrophobicity and *π*–*π* stacking of VPF drove carrier‐free self‐assembly into nanoparticles termed LPN, while intracellular CatB cleavage co‐released VPF and the active PROTAC, coupling photodynamic therapy (PDT) with IDO degradation to remodel the tumor immune microenvironment. Substitution of the IDO‐targeting module with an HK‐2‐targeting PROTAC generated the related NanoTAC platform [121], which retained high drug loading, serum stability, passive tumor accumulation, and biomarker‐selective activation.

Distinct from the two preceding prodrug self‐assembly strategies, the defining value of rigid conjugated motif‐driven systems lies in the therapeutic activity of the driving unit itself. Self‐assembly therefore allows phototheranostic capability and TPD to be integrated within a single nanoparticle, enabling synchronized delivery and intracellular colocalization. These three strategies together define the core framework of single‐component prodrug self‐assembly and reflect a progression from structural simplicity to functional integration and from single‐agent degradation to combination therapy. Hydrophobic small‐molecule‐driven systems establish the feasibility of nanoparticle construction by exploiting the intrinsic hydrophobicity of degraders, oligopeptide‐driven systems extend the functional scope from static delivery to dynamic responsiveness, and rigid conjugated motif‐driven systems introduce a second therapeutic modality without sacrificing delivery capability. However, these studies have thus far focused almost exclusively on cancer and have yet to be meaningfully extended to non‐oncological diseases, which remain an important area for future exploration. For example, although COX‐1/2 also plays pivotal regulatory roles in inflammatory disorders and has long been recognized as an important anti‐inflammatory target [122], SPN_COX_ has so far been validated in vivo primarily in tumor models.

Across both non‐prodrug and prodrug formats, single‐component self‐assembly follows a shared logic of transforming discrete degrader molecules into ordered nanostructures and reshaping their in vivo fate through collective behavior. It should be emphasized, however, that active targeting, stimulus‐responsive release, and therapeutic activity remain encoded in the constituent molecules, whereas self‐assembly primarily organizes and delivers these pre‐existing functions. Its central value lies in enabling passive accumulation through size control, improving stability and circulation through hydrophilic surface engineering, limiting nonspecific activity through nanoscale shielding, and enhancing molecular recognition through multivalent display. It is precisely this functional positioning that distinguishes single‐component self‐assembly from multicomponent co‐assembly, as the former optimizes the delivery of an individual molecular entity whereas the latter collaboratively constructs multifunctional platforms.

## 4 Multicomponent Co‐Assembly Integrates Multifunctionality

Single‐component self‐assembly simplifies the assembly process and obviates the need for auxiliary components [123]. Yet such highly integrated designs can also impose a degree of functional rigidity, as the introduction of additional capabilities often requires re‐engineering and resynthesis of the entire construct, constraining flexibility and extensibility [124]. To circumvent this limitation, increasing attention has shifted to the more versatile strategy of multicomponent co‐assembly. Unlike single‐component systems, in which a single molecule is tasked with fulfilling all functions, multicomponent co‐assembly enables targeting, responsiveness, and therapeutic activity to be introduced through complementary building blocks, thereby expanding functional tunability without requiring complete molecular redesign (Table 3 and Figure S2). This section focuses primarily on degrader‐participating co‐assembly, in which the degrader acts as an active building block and contributes directly to supramolecular organization. These systems directly illustrate how multifunctionality can emerge from the cooperative organization of degraders with complementary functional components. According to whether the degrader is introduced in its pharmacologically active form or as a masked prodrug, the following discussion is organized into non‐prodrug and prodrug‐integrated forms of degrader‐participating co‐assembly (Figure 12).

**TABLE 3:** Summary of supramolecular co‐assemblies for multifunctional TPD.

Platform name	Co‐assembly components	Assembly motif	Assembly mode	Trigger/responsive element	Added function(s)
Fi‐dBET6 NPs/Fi‐MS432 NPs [127]	ICG, fucoidan, dBET6 or MS432 (PROTAC)	Highly hydrophobic PROTAC scaffold	Hydrophobic interactions, *π*–*π* stacking	—	Active targeting (fucoidan to P‐selectin)
BHP NPs [129]	BBR, HPC, dBET57 (BRD4 PROTAC)	Hydrophobic/aromatic/charged domains of all three components	Hydrophobic interactions, *π*–*π* stacking, hydrogen bonding, electrostatic interactions	Acidic pH; protonatable groups	Mitochondrial targeting (BBR), necrosis targeting/deeper tumor penetration (HPC), PDT (HPC), metabolic inhibition, ferroptosis, stimuli‐responsive release
PPET_3_N_2_/P9TC Polyplexes [137]	PPET_3_N_2_, P9TC (aptamer‐PROTAC oligonucleotide)	Cationic side chains and hydrophobic backbone of PPET_3_N_2_	Electrostatic interactions, hydrophobic interactions	—	Enhanced membrane penetration, enhanced cellular internalization, nuclease protection
TPD‐NPs [138]	Antibody‐PEG‐DSPE, PLGA or liposome (HSPC/Chol), optional HSPC/Chol lipid module for liposomal variants	Hydrophobic polymer/lipid core	Hydrophobic interactions	—	Nanoparticle‐mediated endocytosis, noncanonical lysosomal trafficking
NaC4A‐PROTACs [149]	Naph‐SAC4A, MZ1 or dBET6 (PROTAC)	Hydrophobic cavity of Naph‐SAC4A	Host‐guest recognition	Hypoxia (reductive tumor microenvironment); Azo bond (reductive cleavage)	Stimuli‐responsive release
DdLD NPs [142]	DOX, dBET57 (BRD4 PROTAC)	Hydrophobic/aromatic/charged domains of DOX and dBET57	Hydrophobic interactions, electrostatic interactions, *π*–*π* stacking	—	Chemotherapy (DOX), ICD induction (DOX), immunomodulatory synergy (DOX and dBET57)
Ce6‐PROTAC NPs [145]	Ce6, CDK4/6 PROTAC	Hydrophobic and aromatic regions of both components	Hydrophobic interactions, *π*–*π* stacking	—	PDT (Ce6), ICD induction (Ce6), mitochondrial sensitization, G1 arrest
SDNpro [146]	Ce6, dBET57 (BRD4 PROTAC)	Hydrophobic pyrrole/porphyrin moiety of Ce6 and aromatic groups of dBET57	Hydrophobic interactions, electrostatic interactions, *π*–*π* stacking	—	PDT (Ce6), DNA‐damage/repair‐blockade synergy (Ce6 and dBET57)
GM NPs [154]	MTX, GN (IDO AUTAC)	Guanine derivative of GN and nucleoside‐like motif of MTX (base‐pairing‐like hydrogen bonding)	Hydrogen bonding, *π*–*π* stacking, electrostatic interactions	Acidic pH; pH‐sensitive hydrogen bonding between GN and MTX	Chemotherapy (MTX), ICD induction (MTX), autophagy induction (MTX), immunomodulation (GN and MTX), stimuli‐responsive release
PRO‐P [151]	AA‐SS‐Ppa (amphiphilic prodrug), HMGCR‐targeting PROTAC	Hydrophobic segments of AA‐SS‐Ppa and the PROTAC	Hydrophobic interactions, *π*–*π* stacking	GSH; disulfide bond	PDT (Ppa), ferroptosis (PROTAC and Ppa), ICD induction (PROTAC and Ppa), GSH depletion (AA‐SS‐Ppa), lipid metabolism reprogramming, stimuli‐responsive release
POLY‐PROTAC NPs (PGD7/N_3_@PGDA7) [159]	mPEG‐GALGLPG*‐b‐*P(DPA‐r‐ARV771), mPEG‐GALGLPG*‐b‐*P(DPA‐r‐HEMA), PPa‐labeled polymer (PGDA), PED pretargeting NPs (DBCO)	Hydrophobic segment of P(DPA)	Hydrophobic interactions	MMP‐2, acidic pH, GSH; GPLGLAG peptide (MMP‐2), DPA segment (pH), disulfide bond (GSH)	PDT (Ppa), cascade‐responsive release/activation
LND‐MZ1 [156]	HSPC, DSPE‐PEG2k, PE‐S‐S‐MZ1	Hydrophobic tails of phospholipids	Hydrophobic interactions	GSH; disulfide bond	Stimuli‐responsive release/activation
RPG7 NPs/RPG7@DOX [157]	PEG*‐b‐*PLGA‐DT‐ARV771 (or corresponding D43 analog), cRGDK‐PEG*‐b‐*PLGA, DOX as cargo (encapsulated)	Hydrophobic PLGA segment	Hydrophobic interactions	GSH; disulfide bond	Active targeting (cRGDK to NRP‐1), chemotherapy (DOX), responsive release/activation
NP_Ce6+PRO_ [161]	TK‐PROTAC‐PCL, Ce6‐PCL, PEG*‐b‐*PCL	Hydrophobic PCL segment	Hydrophobic interactions	ROS generated by ultrasound; TK linker	SDT (Ce6), ICD induction, reversal of PD‐L1 upregulation, stimuli‐responsive release/activation
PPT7 NPs [163]	PDPA‐PPa, OT7 (OEGs‐TK‐ARV771), BM NPs (separate component)	Hydrophobic segment of PDPA PPa	Hydrophobic interactions	Lysosomal acidic pH, singlet oxygen (^1^O_2_) generated by 671 nm laser; PDPA segment (pH‐responsive protonation), TK linker (^1^O_2_‐cleavable)	PDT (PPa), light‐triggered PROTAC release/activation, and O_2_ generation and hypoxia alleviation by BM nanoparticles

*Note*: The assembly motif denotes the structural unit or a molecular recognition pair that mediates supramolecular organization. The assembly mode summarizes the principal assembly or recognition process reported in the cited studies.

**FIGURE 12:**
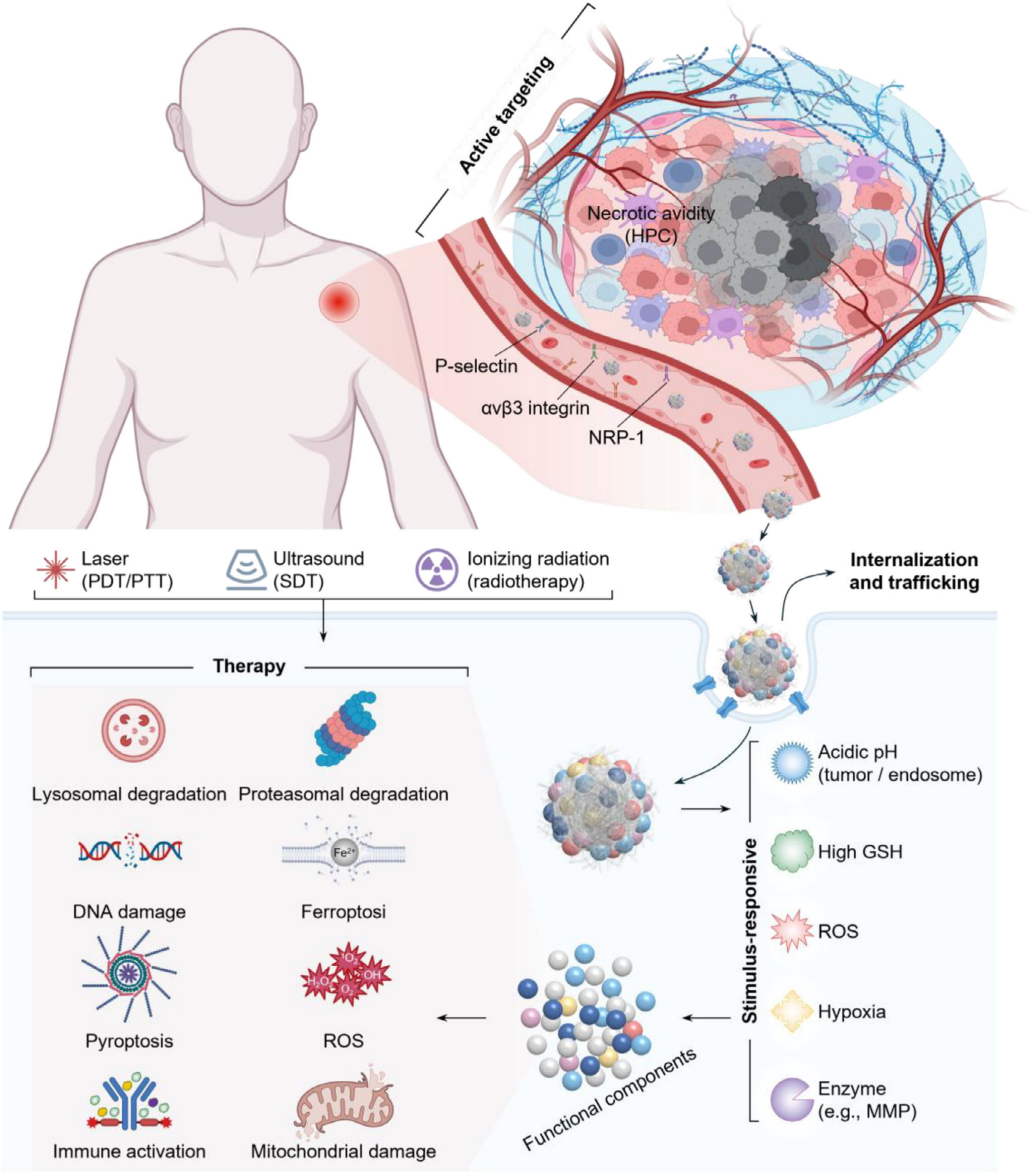
Multicomponent co‐assembly involving covalently constructed degraders. Multicomponent co‐assembled systems containing different functional components can separately or simultaneously mediate active targeting, cellular uptake, intracellular stimulus responsiveness, and synergistic therapy. Active targeting is shown through recognition of P‐selectin, αv*β*3 integrin, NRP‐1, and necrotic tumor regions associated with HPC. Endogenous stimuli include acidic pH, high GSH, ROS, hypoxia, and enzyme activity. Therapeutic processes include lysosomal degradation, proteasomal degradation, DNA damage, ferroptosis, pyroptosis, ROS production, immune activation, and mitochondrial damage.

### 4.1 Non‐Prodrug Degrader‐Participating Co‐Assembly

Non‐prodrug degrader‐participating co‐assembly centers on degraders in their pharmacologically active state and augments them with higher‐order functions, including protection, targeting, responsiveness, and combination therapy, through the incorporation of additional functional components. Within this degrader‐participating class, the reported systems are discussed here according to the distinct functions introduced through co‐assembly, including active targeting, enhanced cellular internalization, therapeutic synergy, and response‐therapeutic coupling.

#### 4.1.1 Active Targeting

Unlike passive accumulation, which is often associated with size‐dependent transport processes [125], active targeting strategies introduce targeting modules through co‐assembly [126] that enable assemblies to recognize specific tissues, cells, or even subcellular structures, conferring a higher degree of selectivity at the delivery stage.

Vogt and colleagues combined cheminformatic screening with structure‐guided co‐assembly to formulate nanoPROTACs (Fi‐dBET6 NPs/Fi‐MS432 NPs, Figure 13A) [127]. Spatial autocorrelation descriptors weighted by ionization potential, particularly the Geary autocorrelation index GATS1i, predicted the assembly propensity of PROTACs. This analysis identified the extended conjugation and planar electronic surfaces of dBET6 and MS432 as favorable for molecular packing with indocyanine green (ICG). Fucoidan further conferred P‐selectin‐directed targeting, exploiting its nanomolar affinity for this adhesion receptor, which is upregulated in tumor‐associated vasculature and other activated vascular and inflammatory settings [128]. The platform therefore integrated predictive assembly with targeted delivery while repurposing the physicochemical features of beyond‐rule‐of‐five degraders as drivers of nanoformulation. A complementary carrier‐free strategy generated BHP nanoparticles (Figure 13B) through co‐assembly of berberine (BBR), hypericin (HPC), and a PROTAC [129]. BBR promoted mitochondrial localization through the mitochondrial membrane potential [130], whereas HPC conferred necrosis avidity, favoring accumulation in necrotic tumor regions and deeper tissue penetration [131]. Their combination thus provided tissue‐ and organelle‐level targeting within a single assembly.

**FIGURE 13:**
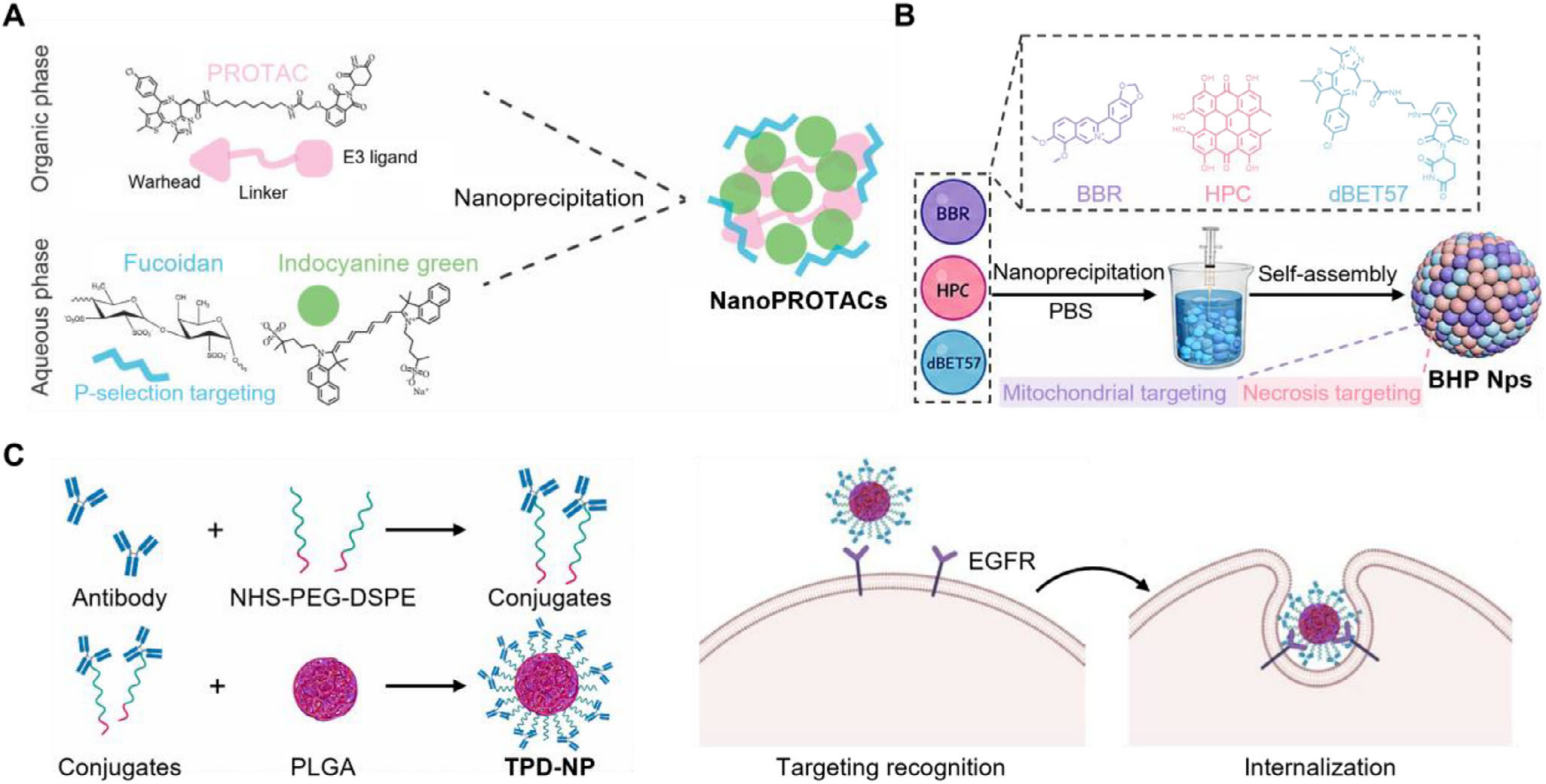
Representative multicomponent co‐assembled platforms for targeted degrader delivery and cellular internalization. (A) Fucoidan‐functionalized nanoPROTACs assembled with ICG for P‐selectin‐targeted delivery. Adapted from Ref [127]. under the terms of the CC BY 4.0 license. Copyright 2025, The Authors, some rights reserved; exclusive licensee American Association for the Advancement of Science. (B) Carrier‐free co‐assembly of BBR, HPC, and dBET57 into mitochondria‐targeting and necrosis‐avid BHP NPs. Adapted with permission [129]. Copyright 2025, Elsevier B.V. (C) Antibody‐decorated PLGA TPD‐NPs for EGFR capture, endocytic internalization, and autolysosomal degradation. Adapted with permission [138]. Copyright 2024, The Author(s), under exclusive licence to Springer Nature Limited.

Together, these studies show that co‐assembly can introduce active targeting through receptor recognition or preferential localization to pathological tissues and subcellular compartments. The resulting selectivity nevertheless depends on the availability of the corresponding receptor or microenvironmental feature, making performance inherently indication‐dependent.

#### 4.1.2 Enhanced Cellular Internalization

For degraders directed against intracellular targets, targeted accumulation alone is insufficient, as the active species still require access to the intracellular compartment to exert their effects. Cellular entry therefore becomes the next major bottleneck, and multicomponent co‐assembly can introduce additional components to help overcome the barriers faced by different classes of degraders during uptake [132].

A range of nanoparticle‐based strategies has been developed to enhance the cellular uptake of degraders, most commonly through receptor‐mediated or nonspecific endocytic pathways [133]. Yet these mechanisms are each subject to intrinsic limitations. For nucleic‐acid‐based degraders, the anionic phosphate backbone generates electrostatic repulsion from the negatively charged cell membrane and hinders membrane association [134]. For membrane‐protein and extracellular‐protein degradation, receptor‐dependent internalization strategies are constrained by the expression of lysosome‐targeting receptors and often require additional receptor‐binding or lysosome‐targeting motifs, limiting their generalizability [135, 136].

To overcome the electrostatic barrier facing nucleic acid degraders, a cationic conjugated polyelectrolyte was co‐assembled with an aptamer‐based PROTAC through electrostatic and hydrophobic interactions, generating polyplexes (PPET_3_N_2_/P9TC) with improved nuclease resistance, a reversed ζ‐potential, and enhanced cellular uptake [137]. This design converted an anionic degrader into a membrane‐compatible assembly, providing a direct physicochemical solution to poor internalization. To circumvent the reliance of membrane‐protein and extracellular‐protein degradation on specific internalization or lysosome‐targeting receptors, a more platform‐oriented solution was proposed [138]. Antibodies were linked to PEG‐DSPE and co‐assembled with clinically approved PLGA to form core‐shell TPD‐NPs displaying antibodies at high surface density (Figure 13C). The antibody corona captured targets such as EGFR, whereas the nanoparticle scaffold drove clathrin‐ and caveolin‐dependent endocytosis and subsequent autolysosomal degradation, bypassing both the intrinsic endocytic activity of the target and auxiliary lysosome‐targeting receptors. Antibody substitution, or co‐display of two antibody–PEG–DSPE conjugates, enabled retargeting to individual or multiple membrane proteins. This strategy is independent of the intrinsic endocytic activity of the target protein and does not require additional lysosome‐targeting receptors, establishing a flexible, plug‐and‐play platform for degrading membrane and extracellular proteins through a non‐canonical internalization route. In brief, these strategies broaden the delivery scope of nucleic‐acid‐based and membrane‐protein‐targeting degradation systems, while showing how particle physicochemical properties can be engineered to regulate degrader uptake and intracellular trafficking [139].

#### 4.1.3 Therapeutic Synergy

Whereas the preceding strategies extend multicomponent co‐assembly from molecular recognition to the control of cellular internalization and trafficking, efficient delivery alone may fail to produce durable therapeutic benefit in complex and refractory diseases. In such settings, protein degradation operating through a singular mechanism is frequently insufficient to overcome drug resistance, the immunosuppressive microenvironment, and tumor heterogeneity [140, 141]. These limitations have motivated co‐assembly strategies that integrate distinct therapeutic modalities within a single platform.

A representative example is the self‐delivery nano‐PROTAC DdLD NPs, formed by co‐assembly of the BRD4 degrader dBET57 with doxorubicin (DOX) (Figure 14A) [142]. DOX induces immunogenic cell death (ICD) and enhances tumor immunogenicity [143, 144], whereas dBET57‐mediated BRD4 degradation inhibits c‐Myc‐dependent glycolysis, reduces lactate accumulation, and suppresses PD‐L1 expression. This mechanistic complementarity combines immune priming with metabolic and immune‐checkpoint regulation, thereby alleviating the immunosuppressive tumor microenvironment and counteracting adaptive immune resistance. Phototherapeutic modules provide a complementary route to mechanism‐based synergy. A hydrophobic CDK4/6‐targeting PROTAC was co‐assembled with chlorin e6 (Ce6) to form carrier‐free Ce6‐PROTAC nanoparticles [145]. The degrader enforced G1 arrest and promoted mitochondrial accumulation and activation, increasing susceptibility to Ce6‐mediated reactive oxygen species generation, apoptosis, and ICD. This coupling of photodynamic injury with cell‐cycle and mitochondrial vulnerability enhanced immune‐cell chemotaxis and remodeled the immunosuppressive tumor microenvironment. A conceptually related carrier‐free assembly, SDNpro (Figure 14B), combined Ce6 with the BRD4 degrader dBET57; Ce6‐induced oxidative DNA damage was reinforced by BRD4 degradation‐mediated suppression of the RAD51–RAD51AP1 repair axis, thereby prolonging PDT‐induced genotoxic stress [146].

**FIGURE 14:**
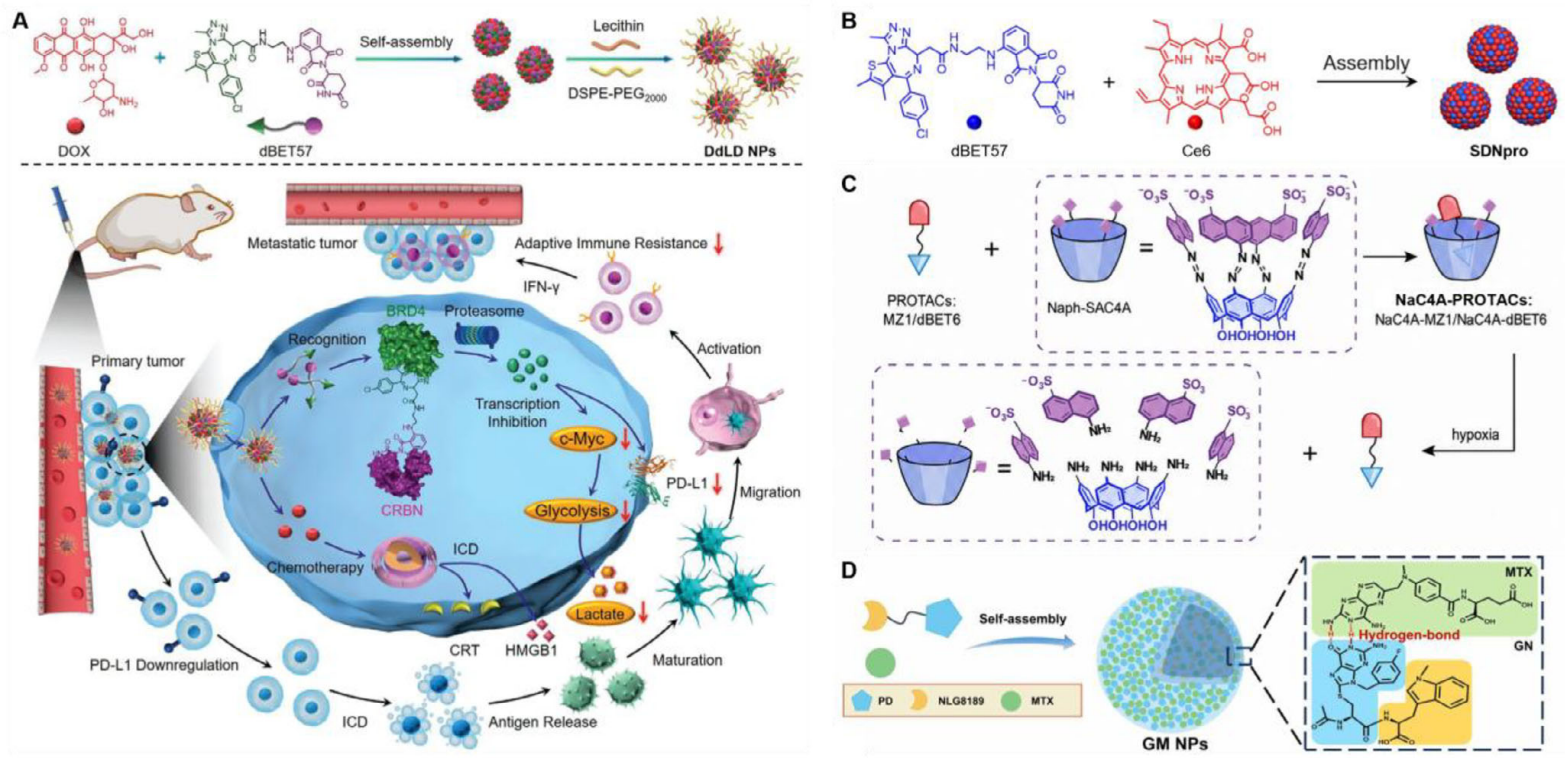
Representative multicomponent co‐assembled platforms for therapeutic synergy and stimulus‐responsive protein degradation. (A) Self‐delivery DdLD NPs co‐assembled from DOX and dBET57 for chemo‐immunotherapy. Reproduced from Ref [142]. under the terms of the CC BY 4.0 license. Copyright 2024, The Authors. Advanced Science published by Wiley‐VCH GmbH. (B) Carrier‐free co‐assembly of Ce6 and dBET57 into SDNpro for combined photodynamic therapy and BRD4 degradation. Reproduced with permission [146]. Copyright 2023, American Chemical Society. (C) Hypoxia‐responsive NaC4A‐PROTACs for conditional activation of BRD4‐targeting PROTACs. Adapted with permission [149]. Copyright 2025, Elsevier B.V. (D) Acid‐responsive GM NPs for combined IDO degradation and methotrexate chemotherapy. Reproduced with permission from Wang et al., Science Advances, 2024, AAAS [154].

#### 4.1.4 Stimulus‐Responsive Therapeutic Synergy

Beyond therapeutic synergy, an additional level of functional integration emerges when environmental responsiveness is directly coupled to therapeutic action. In these systems, stimulus‐triggered events spatially and temporally coordinate degrader exposure, supramolecular disassembly, and downstream therapeutic processes [147]. Responsive co‐assemblies combine degraders with components that remain stable in circulation but dissociate within disease‐associated microenvironments or defined subcellular compartments, improving degradation efficiency and therapeutic precision [148]. The trigger may selectively activate degradation itself or coordinate it with complementary therapeutic pathways.

NaC4A‐PROTACs (Figure 14C) illustrate that response‐therapeutic coupling can be realized through conditional activation of protein degradation itself [149]. In this hypoxia‐responsive host–guest system, an azo‐modified calixarene sequesters PROTAC molecules under normoxic conditions, suppressing ternary‐complex formation and thereby keeping degradative activity largely dormant during circulation. Enzymatic cleavage of the azo bonds in hypoxic tumor regions [150] weakens host–guest affinity, releases the free PROTAC, and restores BRD4 degradation within the malignant niche. This hypoxia‐responsive gating mechanism improves the spatial precision of TPD without introducing a second therapeutic modality.

More extensive functional integration is achieved in PRO‐P, a redox‐responsive HMGCR‐targeting nano‐PROTAC [151]. Disulfide cleavage within the reductive tumor milieu depletes glutathione and increases oxidative vulnerability, while laser irradiation activates pheophorbide a (PA) to generate reactive oxygen species. Concurrent HMGCR degradation disrupts the mevalonate–CoQ10–GPX4 defense axis, promoting ferroptosis and extending the response to photoimmunotherapy through ICD and immune remodeling. Related systems apply this cascade logic through distinct stimuli and degradation pathways. BHP NPs couple acid‐triggered activation and photodynamic stress with BRD4‐degradation‐driven metabolic perturbation to promote ferroptotic tumor‐cell death [129, 152, 153]. Beyond the UPS, acid‐responsive GM NPs (Figure 14D) co‐assembled from the IDO‐targeting AUTAC GN and methotrexate release methotrexate in intracellular acidic compartments, inducing cytotoxic stress and autophagy that favor GN‐mediated IDO degradation and suppress immunosuppression, primary tumor growth, and metastasis [154].

Overall, non‐prodrug degrader‐participating co‐assembly has evolved from a means of physical protection and passive accumulation into a programmable framework for spatiotemporal control and therapeutic coordination. This progression reflects a broader shift in how co‐assembly is used, from a structural scaffold to a strategy for imparting selectivity, controllability, and cooperativity to active degraders without altering their intrinsic pharmacology [155]. Nevertheless, despite the expanded functional scope of non‐prodrug multicomponent co‐assembly, premature exposure of constitutively active degraders remains difficult to eliminate completely. Microenvironment‐responsive and cascade‐responsive designs improve spatial control over release and better confine degradation to pathological settings, but they do not chemically silence the degrader itself. As a result, premature disassembly or leakage can still activate degradation before the intended trigger is encountered, providing a strong rationale for prodrug‐integrated degrader‐participating co‐assembly systems.

### 4.2 Prodrug‐Integrated Degrader‐Participating Co‐Assembly

Prodrug‐integrated degrader‐participating co‐assembly combines functional integration with activity silencing in a single architecture. Degraders remain quiescent in off‐target environments and are activated only within defined spatiotemporal windows. According to the origin of the triggering cue, these systems can be broadly divided into endogenous signal‐responsive and exogenous energy‐mediated systems.

#### 4.2.1 Endogenous Signal‐Mediated Activation

Within endogenous signal‐responsive co‐assembly, prodrug activation has been implemented with increasing functional complexity, from selective restoration of degrader activity to integration with targeting and complementary therapy. LND‐MZ1 exemplifies the former configuration (Figure 15A) [156]. In this system, MZ1 was converted into an amphiphilic phospholipid prodrug through disulfide and ester linkages, with esterification of the VHL‐ligand hydroxyl silencing degrader activity until intracellular GSH restored active MZ1 and BRD4 degradation in tumor cells. RPG7@DOX (Figure 15B) extended this GSH‐responsive design by co‐assembling a disulfide‐linked polymer–PROTAC prodrug with DOX and cRGDK‐bearing chains [157]. Following tumor‐cell entry, GSH restored degrader activity, while cRGDK provided active targeting [158] and DOX supplied chemotherapy, integrating delivery and combination treatment with prodrug activation.

**FIGURE 15:**
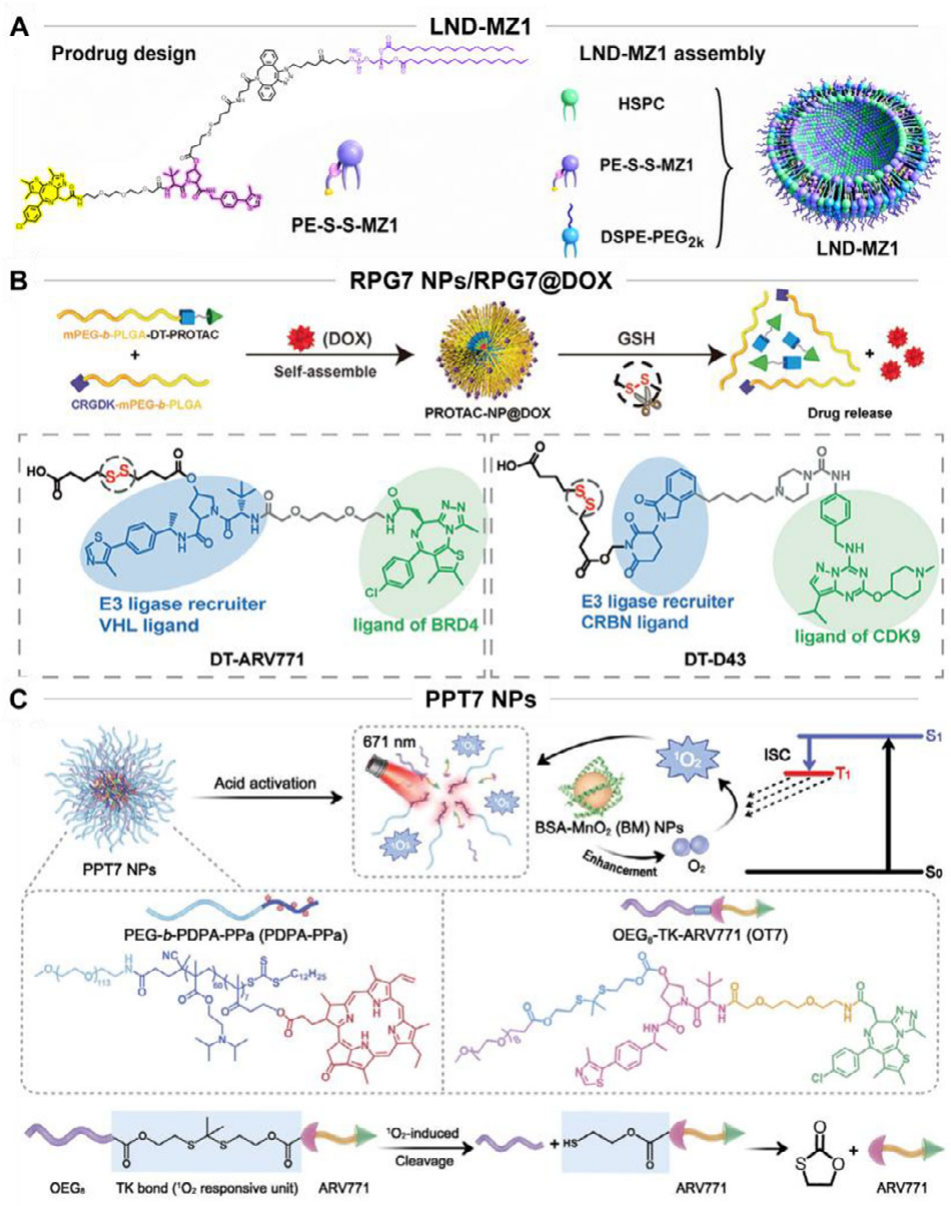
Representative prodrug‐integrated degrader‐participating co‐assemblies for endogenous and exogenous signal‐mediated activation. (A) GSH‐activatable liposomal nano‐PROTAC LND‐MZ1 for BRD4 degradation. Adapted from Ref [156]. under the terms of the CC BY 4.0 license. Copyright 2025, The Author(s). Advanced Science published by Wiley‐VCH GmbH. (B) Reduction‐activatable PROTAC prodrug nanoparticles RPG7 NPs/RPG7@DOX for tumor‐targeted delivery and combination therapy. Reproduced with permission [157]. Copyright 2024, The Author(s), under exclusive licence to Shanghai Institute of Materia Medica, Chinese Academy of Sciences and Chinese Pharmacological Society; published by Springer Nature. (C) Self‐oxygenating PPT7 NPs for light‐triggered ARV771 release and combined photodynamic therapy and BRD4 degradation in glioblastoma. Reproduced with permission [163]. Copyright 2025, Wiley‐VCH GmbH.

POLY‐PROTAC further arranged multiple endogenous cues in sequence [159]. In this platform, methacrylation of the VHL hydroxyl through a disulfide spacer initially silenced BRD4‐degradation activity, and the resulting polymeric prodrug was integrated into a two‐component pretargeted system together with a DBCO‐bearing nanoparticle module. After accumulation in the tumor, extracellular acidity promoted click‐mediated retention of the azide‐functionalized POLY‐PROTAC nanoparticles, whereas MMP‐2 cleavage and endosomal acidification facilitated uptake and disassembly, followed by intracellular GSH‐mediated restoration of the active degrader. A PPa‐containing module additionally enabled irradiation‐triggered PDT, coupling tumor retention, degrader activation, and phototherapy within a staged response cascade.

#### 4.2.2 Exogenous Energy‐Mediated Activation

Although endogenous signal‐responsive hybrid assemblies can improve release precision, their performance remains constrained by the heterogeneity of pathological cues [160] and the increasing complexity of cascade design. Exogenous energy‐mediated activation may address these limitations by using externally applied physical energy as the triggering input, reducing reliance on endogenous variability and enabling tighter spatiotemporal control over degrader release and activation.

NP_Ce6+PRO_ illustrates the use of exogenous energy to activate degraders in deep‐seated lesions [161]. In this system, a BRD4‐targeting PROTAC was silenced by hydroxyl masking through a thioketal linker and co‐assembled, in polymer‐conjugated form, with Ce6 and PEG‐*b*‐PCL. Upon ultrasound irradiation, Ce6‐generated ROS both mediated sonodynamic therapy (SDT) [162] and cleaved the thioketal linkage, restoring the active degrader. Subsequent BRD4 degradation suppressed the PD‐L1 upregulation induced by sonodynamic stress, coordinating SDT, ICD, and protein degradation within the same treatment window.

A complementary platform combined exogenous activation with local delivery and microenvironmental priming in glioblastoma (Figure 15C) [163]. In this platform, a double‐layered microneedle bypassed the blood–brain barrier and confined treatment to the tumor lesion: its outer layer released PPT7 nanoparticles assembled from an acid‐sensitive PPa‐containing polymer and a light‐activatable BRD4 PROTAC prodrug, whereas the inner layer supplied oxygen‐generating BM nanoparticles. Intracellular acidity dissociated PPT7 and restored the photodynamic activity of PPa; subsequent near‐infrared irradiation generated singlet oxygen that mediated PDT [164] and activated the prodrug to release ARV771. By converting H_2_O_2_ into O_2_, BM nanoparticles alleviated hypoxia and amplified both singlet‐oxygen generation and PROTAC activation. In brief, exogenous light functions as the decisive spatiotemporal trigger, whereas acid priming and self‐oxygenation reinforce locally confined PDT and BRD4 degradation in glioblastoma.

Within prodrug‐integrated degrader‐participating co‐assembly systems, chemical masking maintains degrader quiescence in non‐target tissues, whereas physical shielding helps limit premature exposure during circulation. Activation can then be coupled either to endogenous pathological cues or to externally applied physical energy. These two modes offer complementary routes to spatiotemporal control, but each introduces distinct translational constraints. Endogenous responsiveness can integrate targeting and therapeutic cooperation, but becomes increasingly dependent on biological heterogeneity and system complexity. Exogenous activation offers tighter spatiotemporal control, including in deep‐seated lesions, but depends on reliable energy delivery [165, 166]. Whether these activation modes can be robustly implemented in vivo remains a central translational challenge.

More broadly, across both non‐prodrug and prodrug‐integrated systems, multicomponent co‐assembly enables degraders to be integrated with complementary structural and functional elements in ways that are difficult to achieve with single‐component designs. Yet efficient cooperation in vivo cannot be assumed, as different components may still undergo divergent intracellular trafficking and subcellular localization [167]. Progress toward precise control will also require structurally better‐defined assemblies. Current characterization remains dominated by ensemble parameters such as particle size and drug loading, with limited insight into internal organization, ligand presentation, and particle heterogeneity [168]. These unresolved features complicate therapeutic optimization, scale‐up, and quality control.

### 4.3 Boundary Cases in Carrier‐Assembled Degrader Delivery

It should be noted that, beyond degrader‐participating co‐assembly, a related but mechanistically distinct class of delivery systems exists in which the carrier is assembled by other components and the degrader functions primarily as an encapsulated cargo. In these systems, the degrader does not serve as a structural building block or contribute materially to the driving force, architecture, or stability of the assembly. Instead, it is incorporated into nanocarriers formed by amphiphilic lipids, polymers, or proteins, typically within a hydrophobic core or bilayer domain. Although many such formulations are prepared through spontaneous organization in aqueous media and are described as self‐assembled in the literature, their organization is governed primarily by the carrier materials themselves. They are therefore considered separately here as boundary cases, both to distinguish them from degrader‐participating co‐assembly and to acknowledge their substantial importance in biomedical delivery.

Representative systems illustrate how carrier‐assembled formulations can extend the functional envelope of degrader delivery without converting the degrader into an organizing component. For example, liposomal L@NBMZ [169] couples encapsulated BRD4 degrader delivery with photodynamic therapy and pyroptotic killing, whereas the protein nanocage HFn@Thal [170] combines CDK9 degrader loading with intrinsic TfR1‐mediated targeting and acidic release. Polymeric systems further extend this carrier‐centric logic, as illustrated by dBET6@nano‐CXCL9 [171], which uses staged pH responsiveness to coordinate CXCL9 release, BRD4 degradation, and immune modulation. Together with additional carrier‐assembled formulations summarized in Table S3, these examples indicate a progression from degrader solubilization and protection toward targeted, stimulus‐responsive, and multimodal therapeutic orchestration [21, 22, 172, 173, 174, 175, 176, 177, 178, 179, 180].

This functional expansion underscores the biomedical value of carrier‐assembled systems, which adapt established nanomedicine principles to degrader delivery and are often broad in applicability and relatively straightforward to formulate. Their principal limitation, however, is that the degrader remains largely passive during assembly, exerting little influence over carrier architecture, stability, or release behavior. Consequently, encapsulation efficiency, drug loading, retention, and release coupling depend strongly on carrier‐payload compatibility and formulation parameters, and coordinated intracellular action with auxiliary components is not inherently ensured [155, 181, 182, 183, 184, 185]. These considerations do not diminish the importance of carrier‐assembled systems; instead, they delineate the functional boundary of passive cargo delivery and highlight the distinctive value of architectures in which the degrader itself participates in supramolecular organization.

## 5 Intracellular Assembly Creates In Situ Degradation Traps

Single‐ and multicomponent self‐assembly strategies have successfully converted protein degraders into nanomedicines that accumulate efficiently at diseased sites [186]. These approaches generally form assemblies extracellularly, internalize them intact, and then rely on intracellular disassembly to release the active molecular species. Released monomers, particularly PROTACs, may diffuse away from sites of productive engagement or undergo metabolic inactivation, thereby limiting effective intracellular exposure [187]. One strategy for overcoming this bottleneck is to move beyond the paradigm of extracellular assembly followed by intracellular disassembly and instead construct degradation‐competent systems directly within cells, either as persistent supramolecular architectures or as active degraders (Table 4 and Figure S4). These approaches can be broadly divided into in situ physical assembly and in situ chemical assembly according to the dominant architecture‐forming step (Figure 16). This classification reflects how the intracellular architecture is generated, irrespective of chemical transformations that may precede or follow assembly.

**TABLE 4:** Representative intracellular in situ construction strategies for regulating targeted protein degradation.

Platform or system	Key precursor(s)	Intracellular trigger or reaction	In situ‐generated architecture or active species
In situ physical assembly			
Supra‐PROTACs / pro‐Supra‐PROTAC [60]	EIYsIYsE, Bcl‐xL ligand L1, and VHL ligand L2	Sulfatase‐mediated desulfation and peptide assembly	Nanofibrillar Supra‐PROTACs
Nano‐PROTACs [188]	POI‐ and VHL‐binding GNNQQNY peptide precursors	GSH‐activated CuAAC followed by *β*‐sheet assembly	Multivalent Nano‐PROTAC nanofibers
ROS‐instructed supramolecular assembly platform [189]	BQA‐K(Tz)GGFF and TCO‐MZ1	ROS‐induced nanofiber assembly and IEDDA‐mediated decaging	Tz‐bearing nanofibers and released MZ1
Psa‐AR [191]	PSMA‐617–YYQNYQ–AR ligand–CRBN ligand conjugate	PSMA‐mediated uptake and intracellular *β*‐sheet assembly	Psa‐AR nanofibrils
PACES [193]	Tz‐MitoFlag or BQA‐K(Tz)GGGFF, with TP2T	ENTK‐ or ROS‐induced nanofiber assembly followed by IEDDA	CRBN‐recruiting SA‐PROTAC nanofibers
In situ chemical assembly			
Nano‐CLIPTACs [198]	ALK‐TCO precursor W4 and POM‐Tz precursor Z2	Intracellular TCO–Tz IEDDA	ALK‐targeting CLIPTAC WZ42
BT‐PROTAC [199]	TCO‐caged MZ1 and a tetrazine activator	Tz–TCO click‐to‐release reaction	Released MZ1
BTz‐enabled ROS‐activated tetrazine ligation [201]	BTz 5 and TCO‐JQ1 6	H_2_O_2_‐mediated tetrazine formation followed by IEDDA	In situ‐synthesized PROTAC 7
ABC‐PROTAC [202]	Azido‐JQ1 P1, alkynyl VHL ligand P2, and Click‐T‐Cu(II)	GSH‐activated CuAAC catalysis	In situ‐synthesized PROTACs
Split‐Deliver‐Click [203]	JQ1‐DBCO and POMA‐N_3_	Legumain‐ and cathepsin B‐mediated release followed by SPAAC	Click‐PROTAC
Ligation to scavenging system [204]	Tz‐PROTACs and PAMAM‐G5‐TCO	Tz–TCO IEDDA	PAMAM–PROTAC scavenging conjugates

*Note*: Platforms are grouped according to the dominant step that generates the intracellular architecture or active species. Bioorthogonal ligation may precede or follow physical assembly in hybrid systems. ‘ROS‐instructed supramolecular assembly platform’ and ‘BTz‐enabled ROS‐activated tetrazine ligation’ are descriptive labels used in this review because no formal platform names were provided in the original studies.

**FIGURE 16:**
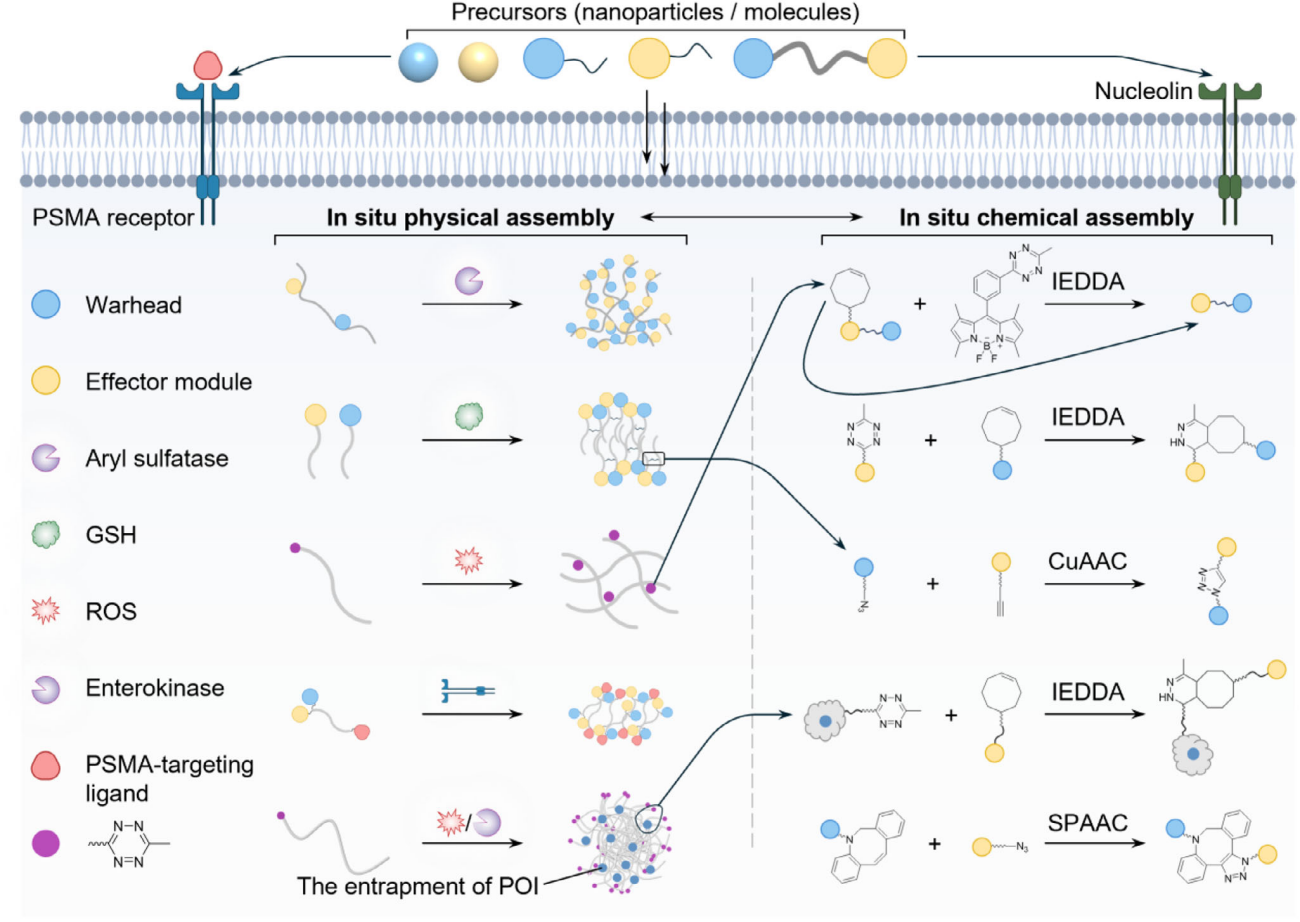
Intracellular in situ assembly strategies in targeted protein degradation. Intracellular in situ assembly is shown as physical assembly and chemical assembly, with intersections between stimulus‐triggered molecular assembly and bioorthogonal chemical reactions. Physical assembly includes assembly processes triggered by aryl sulfatase, GSH, ROS, enterokinase, and combined ROS and enterokinase, with warhead and effector modules organized into intracellular assemblies or POI entrapment structures. Chemical assembly includes IEDDA, CuAAC, and SPAAC reactions that connect functional modules or activate prodrugs within cells.

### 5.1 In Situ Physical Assembly

Physical assembly offers a direct means of constructing functional degradative architectures in situ. The previously described Supra‐PROTAC provides a representative example. Arylsulfatase‐mediated desulfation in tumor cells converts peptide nanoparticles into nanofibers, exposes the functional ligands, and activates PROTAC function in situ [60]. This system establishes enzyme‐triggered morphological reconfiguration as a direct route to intracellular degrader activation. Building on this principle, a subsequent design (Nano‐PROTACs, Figure 17A) coupled intracellular PROTAC formation with nanofiber assembly using two independent precursors. PPOI contains a POI ligand and an alkyne group, whereas PE3 contains an E3‐binding ligand, an azide moiety, and a GSH‐responsive GHK‐Cu(II) catalytic site. Both precursors were further modified with the strongly self‐assembling peptide segment GNNQQNY [188]. After tumor‐cell entry, elevated intracellular GSH reduces Cu(II) to Cu(I). The resulting Cu(I) catalyzes a click reaction between the two precursors, generating a PROTAC monomer that is subsequently driven by the self‐assembling peptide motif into *β*‐sheet nanofibers. Through multivalent display of ligands on the fiber surface, these nanostructures establish a radially organized degradation network that prolongs intracellular retention while also mitigating the hook effect associated with conventional small‐molecule PROTACs.

**FIGURE 17:**
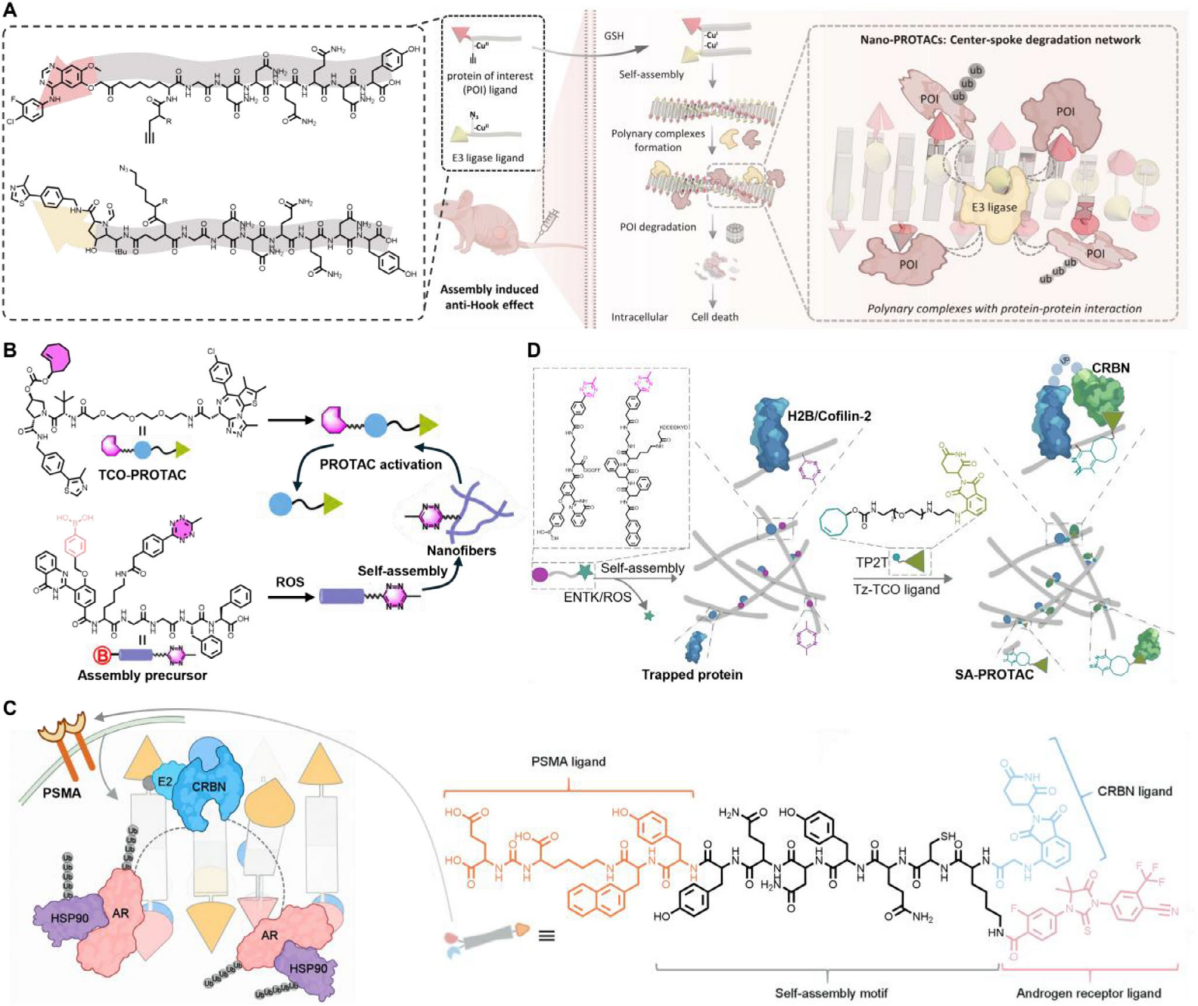
Representative intracellular physical assembly platforms for supramolecular protein degradation. (A) GSH‐triggered in situ click formation and nanofiber assembly of Nano‐PROTACs. Adapted with permission [188]. Copyright 2023, Wiley‐VCH GmbH. (B) ROS‐instructed supramolecular assembly for bioorthogonal activation of PROTACs. Adapted with permission [189]. Copyright 2025, American Chemical Society. (C) Receptor‐triggered self‐assembly of Psa‐AR for AR and HSP90 degradation. Adapted from Ref [191]. under the terms of the CC BY 4.0 license. Copyright 2025, The Author(s), published by Springer Nature. (D) Ligand‐independent protein capture and E3 ligase recruitment by PACES for proteasomal degradation. Adapted in part with permission [193]. Copyright 2025, The Author(s), published by Springer Nature.

ROS‐responsive assembly extended this sequence by generating a localized activation scaffold before prodrug uncaging (Figure 17B) [189]. In pancreatic cancer cells, ROS induced planarization of BQA‐K(Tz)GGFF and promoted π‐π‐stacking‐driven nanofiber formation, leading to intracellular enrichment of tetrazine groups. The fibers subsequently activated a PROTAC prodrug through the inverse electron‐demand Diels‐Alder (IEDDA) reaction [190] and induced BRD4 degradation. Their mitochondrial localization also increased mitochondrial damage and ROS production, establishing a positive‐feedback loop that amplified prodrug activation.

Beyond chemically triggered assembly, receptor recognition offers another route to initiating supramolecular organization. This receptor‐triggered mechanism is exemplified by Psa‐AR (Figure 17C), which integrates a prostate‐specific membrane antigen (PSMA)‐targeting ligand, the self‐assembling peptide segment YYQNYQ, an AR‐binding motif, and a cereblon (CRBN) ligand [191]. The precursor remains a dilute random‐coil monomer in circulation, with a critical assembly concentration of 95.5 µm. Binding to prostate‐specific membrane antigen stabilizes the precursor, lowers the critical assembly concentration to 10.9 µm, and induces a *β*‐sheet transition. Following endocytosis, the nanofiber network matures in the cytoplasm and prolongs retention through the assembly and aggregation‐induced retention effect. The fibers also create confined proximity domains in which AR and its chaperone HSP90 are recruited near cereblon, resulting in degradation of AR, HSP90, and the AR‐V7 splice variant [192].

The preceding systems depend on defined POI ligands. A complementary strategy (PACES) bypassed this requirement by using intracellular nanofibers to capture target proteins through physical interactions, thereby extending degradation to targets that lack suitable ligands (Figure 17D) [193]. This ligand‐independent capture concept was implemented using two tumor‐responsive precursors. Enterokinase (ENTK) cleavage of Tz‐MitoFlag in enterokinase‐overexpressing cancer cells triggered nanofiber formation around mitochondria, whereas elevated ROS induced BQA‐K(Tz)GGGFF to assemble into similar fibers. Both assemblies physically sequestered nearby proteins and subsequently anchored a TCO‐modified CRBN ligand on the fiber surface through a bioorthogonal reaction. This spatial organization brought the captured proteins into proximity with CRBN and initiated proteasomal degradation. The two systems enabled degradation of H2B and Cofilin‐2, two cancer‐associated proteins for which directly acting small‐molecule ligands remain unavailable [194, 195]. The assemblies therefore assume a functional role beyond tumor‐selective activation and precursor delivery by acting as intracellular target‐capture interfaces. This design addresses a central limitation of conventional PROTACs and enables degradation without a pre‐existing target ligand [196].

Physical assembly strategies rely on endogenous signals or target recognition to trigger noncovalent assembly. Their principal advantages are a relatively simple design logic and the ability to exploit the intracellular milieu directly for in situ activation and structural retention. Nevertheless, the performance of these systems generally depends on the specificity of the triggering cue and the degree of control over the assembly process, and their generalizability remains to be improved. Together, these studies define two complementary roles for intracellular physical assembly. Ligand‐based systems stabilize productive proximity and prolong degrader residence, whereas capture‐based systems extend degradation to proteins that lack suitable ligands. Given that many disease‐associated proteins remain inaccessible because suitable ligands are unavailable [197], the development of intracellular in situ assembly systems based on physical capture holds substantial potential.

### 5.2 In Situ Chemical Assembly

Unlike physical assembly, which depends on weak intermolecular interactions and conformational transitions, chemical assembly forges new covalent linkages in situ via intracellular bioorthogonal reactions, enabling the precise construction of functional degradative architectures. Although based on covalent bond formation, this strategy pursues the same overarching objective of building functional structures in situ and has likewise been classified within the broader concept of self‐assembly in the literature (Nano‐CLIPTACs, Figure 18A) [198]. It may therefore be regarded as an alternative, more broadly defined route to intracellular assembly.

**FIGURE 18:**
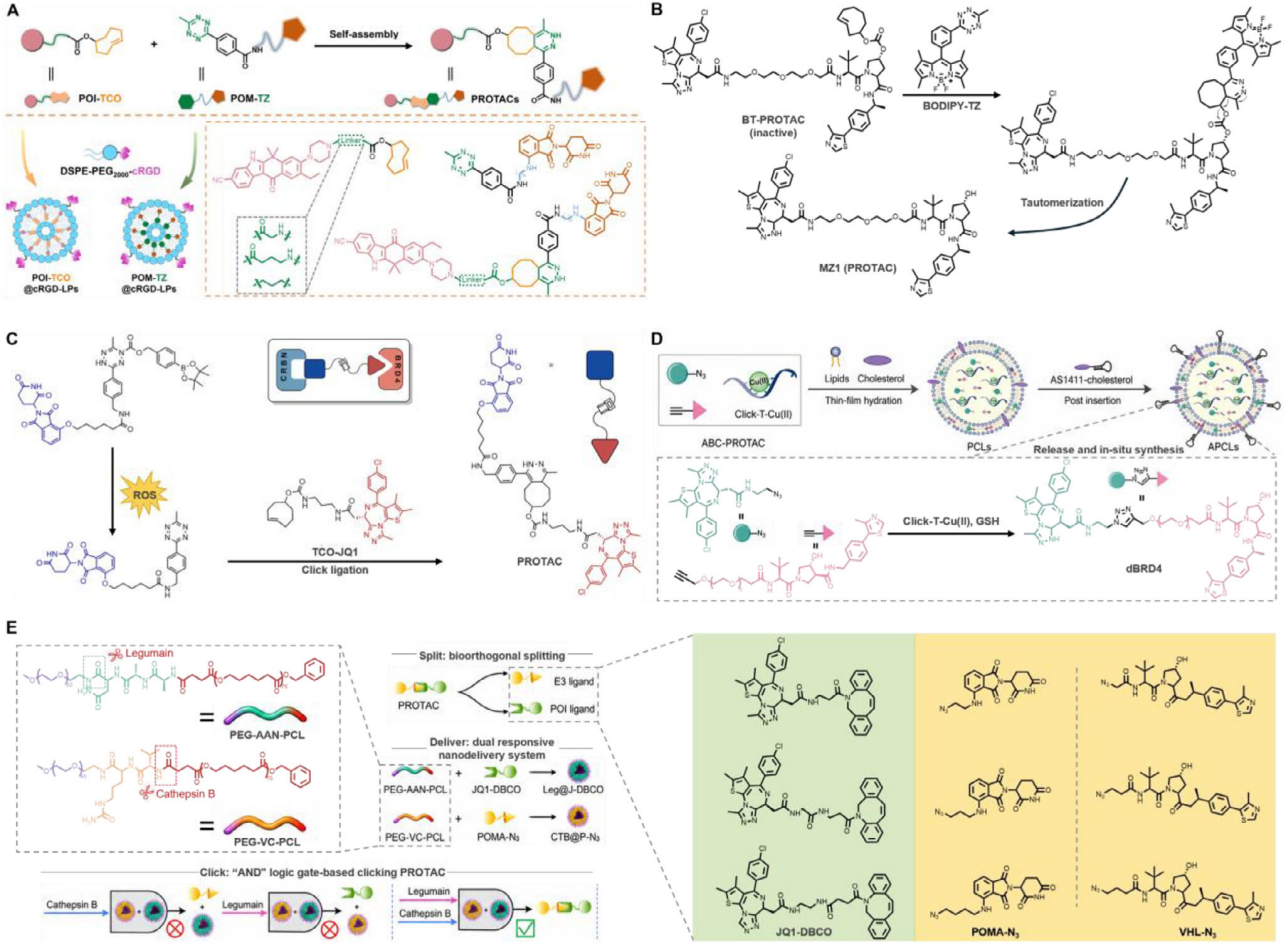
Representative bioorthogonal strategies for the in situ construction and activation of PROTACs. (A) Nano‐CLIPTACs formed through bioorthogonal ligation of separately delivered PROTAC precursors. Adapted with permission [198]. Copyright 2024, Wiley‐VCH GmbH. (B) BODIPY–TZ‐triggered bioorthogonal activation of BT‐PROTAC and release of MZ1. Independently redrawn based on Ref [199]. (C) H_2_O_2_‐responsive construction of a BRD4‐targeting PROTAC. Adapted with permission [201]. Copyright 2026, The Author(s), published by Springer Nature. (D) GSH‐activated ABC‐PROTAC for intracellular PROTAC synthesis. Adapted with permission [202]. Copyright 2025, American Chemical Society. (E) Dual‐enzyme‐responsive Split‐Deliver‐Click platform for logic‐gated PROTAC formation. Adapted with permission [203]. Copyright 2026, Wiley‐VCH GmbH.

Notably, some of the physical assembly systems discussed above already incorporate bioorthogonal reactions. Examples include click‐mediated ligation of precursors before assembly [188] and post‐assembly anchoring of effector molecules via the IEDDA reaction [189]. In these systems, bioorthogonal chemistry and physical assembly act in concert to generate intracellular functional architectures with degradative activity. Bioorthogonal reactions can also independently enable the intracellular formation and conditional activation of active degraders. As a proof of concept, trans‐cyclooctene (TCO) was introduced into the PROTAC MZ1 to generate the inactive prodrug BT‐PROTAC. Bioorthogonal reaction with BODIPY–TZ triggered MZ1 release and restored BRD4 degradation in solution and in cells, whereas the tumor‐targeting tetrazine IR808–TZ enabled pretargeted activation of BT‐PROTAC in vivo (Figure 18B) [199]. The key design implication is that a functional degrader can be generated in situ through bioorthogonal ligation of otherwise inactive precursors, without relying on a preassembled nanostructure.

Because bioorthogonal reactions proceed efficiently and selectively in living systems without perturbing endogenous biochemical processes [200], their triggering mechanisms have increasingly been coupled to characteristic features of the tumor microenvironment. One ROS‐responsive design combined a boronic ester‐caged, dihydrotetrazine‐modified thalidomide with a TCO‐tagged PROTAC, enabling elevated tumor ROS to initiate the IEDDA reaction and generate the active PROTAC on demand (Figure 18C) [201]. A complementary strategy (ABC‐PROTAC, Figure 18D) exploited the elevated intracellular GSH concentration in tumor cells by engineering a Click‐T‐Cu(II) complex as a bioorthogonal precatalyst and co‐encapsulating an azide‐modified BRD4 ligand and an alkyne‐modified VHL ligand within AS1411 aptamer‐decorated liposomes [202]. Upon entry into tumor cells, elevated GSH reduces Cu(II) to Cu(I), which catalyzes the copper‐catalyzed azide‐alkyne cycloaddition (CuAAC) between the two ligand modules to generate the active PROTAC in situ and induce BRD4 degradation. A dual‐enzyme‐responsive nanoplatform (Split‐Deliver‐Click) extended this principle to logic‐gated activation (Figure 18E) [203]. In that system, a PROTAC was divided into two modules that were separately loaded into legumain‐ and CatB‐responsive polymeric micelles, respectively. Exposure to both enzymes triggered disassembly of the corresponding micelles, allowing the released modules to reconstitute the active degrader through strain‐promoted azide‐alkyne cycloaddition (SPAAC).

Beyond initiating degradation, bioorthogonal chemistry can also be deployed to terminate an ongoing degradation process on demand. Jin and colleagues exploited the IEDDA reaction to sequester Tz‐PROTAC on a dendrimer scaffold, generating a sterically encumbered conjugate that prevents engagement with both the target protein and the E3 ligase, arresting the catalytic degradation cycle [204]. Chemical assembly can therefore provide programmable control over both the initiation and termination of degradation. Other bioorthogonal chemistry‐based degradation systems have been comprehensively reviewed elsewhere [205]. These include combinatorial ubiquitination rEal‐time PROteolysis (CURE‐PROs) enabled by reversible phenylboronic acid‐catechol interactions [206], LYTACs driven by sulfur(VI)‐fluoride exchange (SuFEx) click chemistry [207], and ATTECs assembled through CuAAC reactions [208, 209]. They are therefore not discussed further here.

Bioorthogonal chemistry allows degraders to enter cells as precursors, silent modules, or split components that are reconstituted into active architectures only at the target site, thereby improving spatiotemporal control. Nonetheless, strategies that rely exclusively on intracellular chemical reactions to initiate or terminate degradation still face substantial limitations, chiefly because the repertoire of available bioorthogonal reactions remains restricted and individual reactions often suffer from limitations in reaction kinetics, substrate accessibility, operational simplicity, biocompatibility, or functional‐group stability [210]. In response, the field is increasingly moving toward combinations of chemical and physical assembly. These two modes provide complementary layers of regulation. Physical assembly exploits endogenous signals to achieve in situ enrichment and prolonged retention, with weak intermolecular interactions driving cooperative supramolecular organization. Bioorthogonal chemistry, in turn, enables spatiotemporally controlled covalent bond formation and provides explicit chemical switches for initiating or terminating degradation. Their integration combines intracellular enrichment and retention with precise chemical control, thereby expanding the functional scope of intracellular self‐assembly.

## 6 Translational Challenges and Future Opportunities

From the noncovalent decoupling of functional modules to delivery optimization through single‐component self‐assembly, and from multifunctional integration via multicomponent co‐assembly to intracellular in situ assembly that extends beyond conventional delivery paradigms, these strategies have broadened the design space and functional repertoire of degraders. As this interdisciplinary field moves from proof of concept toward translational development, several shared bottlenecks remain.

These bottlenecks span multiple stages of translation, from degrader design and indication matching to biofluid stability, tissue delivery, cellular entry, and intracellular routing. At the upstream stage, limited design rules, restricted assembly motifs, and unclear indication matching constrain the extension of modular, ligand‐independent, and disease‐tailored platforms beyond selected proof‐of‐concept systems. Once deployed in vivo, supramolecular degraders must maintain functional assembly in complex biofluids. Blood dilution, biofluid‐mediated degradation, and nano‐bio interfacial interactions may compromise assembly integrity, stoichiometric control, and controlled degrader exposure [93]. Dilution‐sensitive assemblies, nuclease‐vulnerable nucleic‐acid scaffolds, and sequence‐ or microenvironment‐sensitive peptide assemblies represent distinct manifestations of this broader robustness problem [68, 83, 93]. These stability issues are further coupled to biodistribution and cellular uptake through protein‐corona formation, immune recognition, and reticuloendothelial clearance. After internalization, the limiting step often shifts to productive intracellular routing. UPS‐directed and some autophagy‐related systems require cytosolic access after endocytosis, whereas insufficient endosomal escape can divert assemblies toward lysosomal sequestration or functional inactivation; lysosome‐directed platforms instead must align receptor engagement, endocytic trafficking, target localization, and lysosomal delivery. Together, these linked barriers frame the following sections, where each challenge is paired with corresponding future opportunities (Figure 19).

**FIGURE 19:**
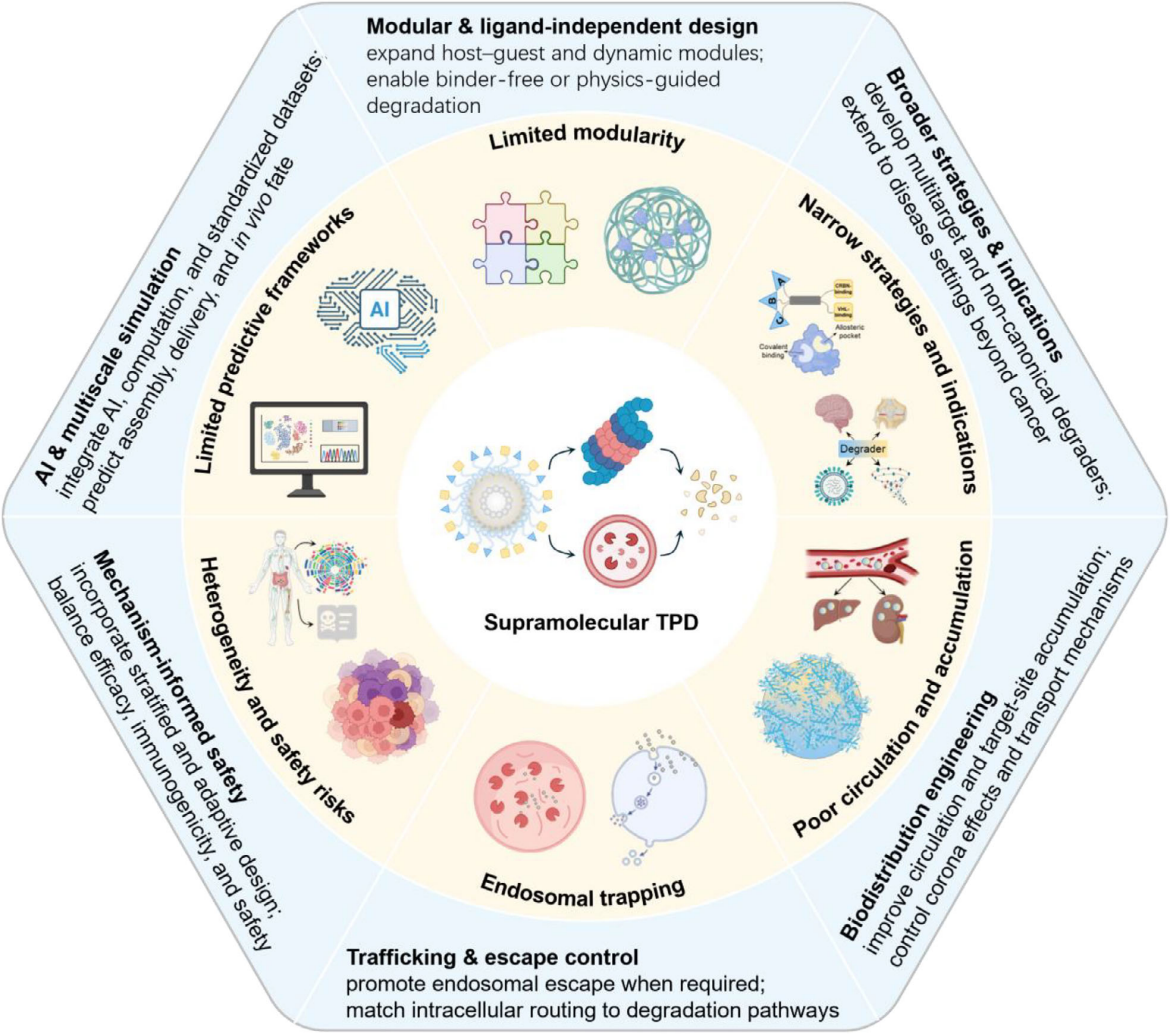
Translational challenges and future opportunities for supramolecular TPD. The inner ring summarizes six major translational challenges, including limited modularity, narrow strategies and indications, poor circulation and accumulation, endosomal trapping, heterogeneity and safety risks, and limited predictive frameworks. The aligned outer sectors present corresponding opportunities, including modular and ligand‐independent design, diversification of degradation strategies and indications, biodistribution engineering, trafficking and escape control, mechanism‐informed safety assessment, and AI‐assisted predictive modeling and multiscale simulation. AI, artificial intelligence; CRBN, cereblon; TPD, targeted protein degradation; VHL, von Hippel–Lindau.

### 6.1 Broadening Modular Design and Ligand‐Independent Degradation

Conventional protein degraders are constrained by covalent linkage architectures that often require synthetically demanding routes, prolonged optimization, and uncertain outcomes. Supramolecular chemistry offers an alternative by decoupling target recognition from recruitment of the degradation machinery through noncovalent interactions, allowing distinct modules to be combined through straightforward admixture and self‐assembly and potentially reducing the optimization burden of conventional chimera engineering. Despite early demonstrations of modular supramolecular TPD, the available design repertoire remains limited, reflecting both the narrow range of self‐assembling motifs and the underuse of established supramolecular paradigms, particularly host–guest recognition. Host–guest interactions are already widely used in pharmaceutical formulations [211, 212] and clinically approved agents such as Sugammadex demonstrate the translational viability of supramolecular recognition [213]. Expanding host–guest systems and other dynamic linking modules therefore represents a clear priority for the field.

Target‐ligand availability presents a separate bottleneck. A large fraction of human proteins, including transcription factors, scaffold proteins, and many other ostensibly undruggable targets, remain inaccessible to high‐quality warheads [214]. Notably, recent work has shown that the intracellular in situ assembly of nanofibers can physically sequester target proteins and enable the degradation of ligand‐deficient targets [193], providing a potential route beyond ligand‐dependent recognition. The approach, however, remains confined to selected systems, and the molecular basis of target capture is still poorly defined, limiting broader generalization. Its wider application will depend on resolving two central questions, namely what governs selective protein capture and how sequestered proteins can be delivered efficiently and stably to the relevant degradation machinery. Systematic investigation of these mechanisms will be required to extend current proof‐of‐concept studies across a broader target landscape.

Beyond ligand‐independent capture, the recently reported TPD‐NPs illustrate a second design principle in which degradation can be achieved without conventional effector modules by harnessing nanoparticle‐governed cellular trafficking following target engagement. Because particle size, shape, and stiffness influence uptake routes and intracellular fate, tuning these properties may direct degraders toward defined endocytic or autophagic pathways [138]. This physics‐guided strategy could support programmable degradation platforms that exploit diverse trafficking routes while reducing reliance on elaborate molecular engineering.

These effector‐independent principles may also inform canonical UPS‐directed PROTACs, for which the repertoire of E3 ligase ligands remains severely restricted. Most degraders in clinical evaluation still rely on CRBN or VHL, while ligand discovery for other E3 ligases is constrained by limited tractability, tissue specificity, and compatibility at the degradation‐recruitment interface [215]. A comparable physics‐guided strategy for UPS‐directed degradation could, in principle, reduce dependence on specific E3‐binding ligands and ease current ligand‐discovery constraints. Together, expansion of the modular toolkit, ligand‐independent protein capture, and physically guided degradation may extend supramolecular TPD beyond conventional bifunctional designs that couple target recognition with recruitment of the degradation machinery.

### 6.2 Diversifying Strategies and Indications Beyond Oncology

Multitarget synergy can enhance therapeutic efficacy and help overcome drug resistance, driving the development of increasingly diverse multitarget degradation strategies, including trivalent PROTACs [216]. Current supramolecular degraders, however, remain focused predominantly on single‐target degradation, and cooperative multitarget assembly is rare, with AS‐TD2‐PRO among the few reported examples [77]. Future efforts should define the design principles, stoichiometric requirements, and applicability of multicomponent co‐assembly for synergistic multitarget degradation and determine whether such systems can be generalized across TPD platforms.

More broadly, most existing supramolecular PROTACs still follow the design logic of conventional noncovalent orthosteric inhibitors and have yet to incorporate noncanonical modalities such as allosteric or covalent PROTACs. Recent advances in allosteric and covalent modulation suggest potential gains in selectivity and resistance control [217, 218], providing a basis for adapting these modalities to supramolecular systems. Recent developments in non‐UPS degradation pathways may likewise inform the design of new supramolecular platforms [219]. Such systems, however, often introduce additional layers of assembly, delivery, and intracellular trafficking, increasing mechanistic complexity relative to conventional degraders. Establishing broadly applicable design principles will therefore require systematic validation across distinct targets, assembly modes, and degradation pathways.

The current supramolecular degrader landscape remains overwhelmingly dominated by oncology, reflecting the early stage of the field and the limited exploration of broader indications. Protein degradation technologies have already shown substantial potential in immunomodulation [220], neurodegenerative disease [221], antiviral therapy [222], and other settings [223], providing a foundation for extending supramolecular strategies beyond cancer. Supramolecular assemblies also offer considerable flexibility in engineering tissue distribution and cellular homing. Parameters such as particle size, surface charge, and morphology can influence biodistribution across organs, including the liver, spleen, and kidney, or bias uptake toward macrophages, endothelial cells, or specific subcellular compartments [224, 225]. Future development should therefore prioritize indications in which tissue‐homing requirements, cellular uptake pathways, and target biology align with the properties of the supramolecular assembly. Expansion of the indication spectrum should be guided by this biological and delivery‐based alignment.

Among potential indications, central nervous system disorders are particularly challenging because of the BBB [226]. Nanomedicines can access transport mechanisms unavailable to many conventional small molecules, including receptor‐mediated and adsorptive‐mediated transcytosis [227, 228]. Yet brain‐directed applications of supramolecular degraders remain rare. The trafficking‐enabled TPD‐NPs discussed above provide an early proof of concept for systemic brain delivery [138]. In an orthotopic glioma model, intravenously administered degradative nanoparticles partially crossed the BBB, increased tumor accumulation, reduced intratumoral target levels, and prolonged survival. Incorporation of the BBB‐targeting ligand angiopep‐2 further enhanced these effects. These findings support the feasibility of brain‐directed supramolecular degradation while underscoring the difficulty of maintaining assembly integrity, target engagement, and productive intracellular trafficking after barrier crossing. Effective brain delivery will therefore require coordinated optimization of systemic circulation, barrier transport, tissue accumulation, and intracellular degradation.

### 6.3 Improving Systemic Circulation and Target‐Site Accumulation

After entering the bloodstream, self‐assembled nanomedicines undergo rapid distribution and clearance by the mononuclear phagocyte system, with hepatic and splenic sequestration constituting major barriers to sustained circulation [229]. The liver is a major clearance organ, where liver sinusoidal endothelial cells and Kupffer cells drive nanoparticle sequestration through endothelial scavenging and phagocytic uptake [230]. Depending on their size and composition, nanoparticles may also undergo renal filtration or redistribution to other organs. Their circulation behavior and ultimate in vivo fate are therefore strongly shaped by material composition, particle size, surface chemistry, and dose [231]. Sustained intravascular residence therefore requires supramolecular degraders to navigate these systemic barriers effectively.

At the nano‐bio interface, these clearance processes are further shaped by the formation of a protein corona [232]. In biological fluids, proteins rapidly and reversibly adsorb onto nanoparticle surfaces to form a dynamic corona whose composition is influenced by particle size, surface charge, hydrophobicity, and surface functionalization [233]. The resulting corona redefines the biological identity of nanoparticles, modulating cellular uptake, phagocytic clearance, and organ distribution. The corona can mask targeting ligands, attenuate tissue selectivity, and enhance phagocytic recognition and clearance [234]. Conversely, adsorbed proteins with intrinsic homing or receptor‐recognition properties may promote tissue‐specific delivery [235], Yet a systematic understanding of how corona composition and temporal evolution influence the delivery behavior and downstream degradation performance of supramolecular TPD systems remains lacking. Detailed proteomic characterization, together with rational control of surface composition, interfacial hydration, and hydrophilic‐hydrophobic balance, may favor corona profiles that preserve ligand accessibility, limit premature phagocytic clearance, and promote interactions with disease‐relevant tissues or cell populations [236].

Once sufficient circulatory persistence is achieved, the next challenge is tumor entry and retention. Early nanomedicine research centered on the enhanced permeability and retention (EPR) effect, whereby nanoparticles passively enter tumors through hyperpermeable vasculature and are retained because of impaired lymphatic drainage [237]. This size‐dependent extravasation‐and‐retention paradigm long served as a cornerstone of nanomedicine design. More recent evidence has highlighted substantial heterogeneity in the EPR effect across tumor types, among patients, and within individual tumors, limiting its robustness as a general model of delivery [238]. This reassessment has brought increasing attention to the active transport and retention (ATR) mechanism, which emphasizes the roles of transcellular endothelial pathways and tumor‐associated lymphatic drainage in determining nanoparticle accumulation alongside passive extravasation [239, 240]. In supramolecular TPD systems, the relative contributions of passive extravasation and active transcellular transport to intratumoral accumulation and degradation efficacy remain unresolved across different tumor microenvironments. Spatially resolved imaging and quantitative biodistribution studies may help clarify how vascular permeability, endothelial transport activity, stromal interactions, and receptor availability shape net tumor accumulation, thereby informing the selection of particle properties and targeting strategies for distinct contexts.

Together, these barriers highlight the need for systematic structure‐property‐performance relationships that capture the stage‐dependent effects of particle size, morphology, surface charge, hydrophilic‐hydrophobic balance, ligand density, and assembly stability across the delivery‐degradation cascade. Importantly, the same feature may exert different or even opposing effects during systemic circulation, target‐site accumulation, cellular uptake, and intracellular release. For example, surface characteristics that reduce nonspecific interactions and prolong circulation may also weaken cellular engagement. Enhanced assembly stability in blood may likewise impede disassembly or functional reorganization after internalization. Particle dimensions that favor tissue penetration may also differ from those required for prolonged retention or avoidance of renal and reticuloendothelial clearance. Future studies should therefore evaluate these properties across successive biological barriers and avoid optimizing individual delivery steps in isolation. Defining context‐dependent design windows and the trade‐offs among systemic stability, tissue accumulation, cellular entry, and degradation output may yield generalizable design principles for the rational development of supramolecular degrader systems.

### 6.4 Overcoming Endosomal Escape and Lysosomal Sequestration

For nanoparticle‐based TPD systems that rely on the UPS for intracellular protein degradation, efficient cellular uptake alone is insufficient. Their activity often depends on productive endosomal escape and subsequent cytosolic access. Failure to escape from early endosomes can direct these assemblies toward late endosomes and lysosomes, where the active cargo may be inactivated or degraded [241]. Studies of lipid nanoparticle mRNA delivery suggest that only 3%–7% of cargo achieves functional cytosolic release, underscoring the gap between cellular uptake and effective intracellular delivery [242]. This issue is particularly relevant to UPS‐directed nanoparticle‐based TPD systems, which require access to cytosolic degradation machinery.

Several strategies have been developed to promote endosomal escape. These include pH‐responsive moieties that undergo conformational changes or destabilize membranes under acidic conditions, cationic polymers that exploit proton‐sponge effects to induce osmotic imbalance, and ionizable lipids that undergo phase transitions during endosomal acidification and promote fusion with endosomal membranes [243]. Intracellular in situ assembly may also address this barrier directly. For example, alkaline phosphatase‐catalyzed dephosphorylation can drive the in situ conversion of peptide nanoparticles into nanofibers within the endosomal lumen. The mechanical forces generated by fiber growth may disrupt the endosomal membrane [244]. Likewise, in ionizable lipid‐based systems, endosomal acidification can drive a transition from disordered internal structures to inverse hexagonal or cubic phases, generating membrane‐fusogenic architectures that favor endosomal escape [245].

Intracellular in situ assembly offers several advantages for TPD, including enhanced target retention through physical confinement, stimulus‐responsive control over degradation activity, and expansion of the targetable landscape of undruggable proteins through physical sequestration. Integrating endosomal escape with intracellular functional amplification within a single assembly strategy may improve degradation efficiency and enable more sophisticated multifunctional platforms.

Distinct degradation pathways also impose different intracellular trafficking requirements. Systems designed to degrade intracellular targets through the UPS or certain autophagy‐related routes are often limited by insufficient endosomal escape and lysosomal sequestration. Lysosome‐directed strategies for extracellular or membrane proteins instead depend on efficient lysosomal delivery. Future work should therefore define the delivery requirements of each TPD modality by aligning the degradation pathway with target localization and intracellular trafficking fate.

### 6.5 Addressing Heterogeneity, Immunogenicity, and Long‐Term Safety

Stimuli‐responsive assembly systems that exploit endogenous cues, including GSH, ROS, and MMP‐2, have been widely used for tumor‐selective delivery. Yet these signals vary markedly among patients, between primary and metastatic lesions, and throughout treatment [246]. Although many current designs seek to sharpen specificity through multilock and multikey response logic, reliance on population‐averaged pathological parameters may still compromise performance in individual patients. Future studies should integrate patient stratification, pretreatment biomarker profiling, longitudinal lesion monitoring, and adaptive design to define indication‐ or patient‐specific activation windows and improve the clinical predictability of supramolecular TPD systems.

To reduce dependence on heterogeneous endogenous cues, several studies have integrated photothermal, photodynamic, or sonodynamic modules with degrader functionality within a single nanoplatform, enabling spatiotemporally controlled activation and synergistic therapy through co‐assembly [117, 120, 129, 163]. These modalities nevertheless face distinct constraints. Light‐based modalities are constrained by limited tissue penetration, whereas ultrasound‐ and heat‐mediated interventions require careful control of energy deposition to minimize unintended thermal or mechanical injury to surrounding tissues [247, 248]. Their use in nanostructured degraders should therefore be calibrated to lesion depth, anatomical accessibility, and surrounding‐tissue tolerance, with gains in therapeutic efficacy weighed against stimulus‐associated toxicity.

Immunological interactions introduce a less predictable layer of complexity, making immunogenicity a major barrier to the clinical translation of nanoparticle‐based degraders. Materials such as cationic polymers, liposomal components, and peptide scaffolds can trigger innate immune recognition, leading to rapid clearance, inflammation, hypersensitivity, and autoimmune‐like reactions that compromise delivery and safety [249]. Conversely, controlled immune stimulation may potentiate antitumor immunity through immunogenic cell death (ICD) and act synergistically with protein degradation [250]. Future work should therefore distinguish therapeutically productive immune activation from systemic or persistent inflammation and define how these responses vary with material properties, disease context, and dosing regimen. Longitudinal assessment of complement activation, cytokine release, and adaptive immune responses, particularly under repeated dosing, may help establish acceptable windows of immune activation [251].

Clinical translation will ultimately require rigorous safety assessment across these approaches, including combination therapy and immune modulation. At present, most studies rely on conventional readouts such as changes in body weight and histopathology of major organs. For event‐driven, nanoparticle‐based degraders, however, long‐term risks are more complex. These risks include chronic functional impairment caused by sustained target degradation in normal tissues, toxicity arising from organ accumulation of carriers or assemblies, complement activation and immune clearance, and collateral injury associated with premature release, off‐target degradation, ectopic activation, or combination therapy. Safety assessment should therefore distinguish among degradation‐related, material‐related, and immune‐mediated toxicities and examine degradation selectivity and reversibility, carrier persistence and clearance, and cumulative effects under repeated dosing. The field, however, lacks tailored and standardized assessment tools and toxicological frameworks for long‐term follow‐up. A mechanism‐informed framework that links therapeutic efficacy to molecular, material, immune, and organ‐level toxicity over time will be essential for advancing supramolecular TPD systems toward clinical translation.

### 6.6 Toward AI‐Assisted Predictive Modeling and Multiscale Simulation

In recent years, AI and computational modeling have increasingly influenced several areas of biomedical research and drug discovery. Structure‐prediction platforms typified by AlphaFold have substantially advanced three‐dimensional protein‐structure modeling, providing useful foundations for target identification and molecular design in drug discovery [252]. AI‐assisted approaches are also beginning to be explored in TPD, particularly for conventional PROTAC design. For example, AI‐assisted design has contributed to the identification of selective aurora kinase B (AURKB) degraders, which showed encouraging activity in models of acute lymphoblastic leukemia [253]. Structure‐aware frameworks such as DeepDegradome have also provided early examples of computationally supported degrader generation, from fragment assembly to candidate design, with validation on WDR5 and CDK9. Selected predicted conformations closely matched those resolved by X‐ray crystallography [254]. Collectively, these studies illustrate the emerging, although still early, contribution of computational methods to conventional PROTAC design.

AI‐assisted methods have also been explored in adjacent areas, particularly nanoparticle formulation and pharmacokinetic modeling. By coupling automated liquid‐handling platforms with machine learning, researchers have built formulation datasets and improved prediction of nanoparticle formation, facilitating nanoformulation optimization for difficult‐to‐encapsulate drugs such as venetoclax and trametinib [255]. In pharmacokinetics, multiview deep‐learning models and experimentally informed PBPK frameworks have begun to relate selected physicochemical parameters, including particle size and zeta potential, to organ distribution [256, 257]. These advances in degrader design and nanomedicine provide a prospective methodological basis for AI‐assisted modeling of supramolecular degraders.

Nevertheless, computational modeling of nanoparticles and self‐assembled systems remains more challenging than modeling classical protein‐protein or ligand‐protein interactions. This complexity reflects their structural heterogeneity, conformational flexibility, polydispersity, and dynamic nano‐bio interfaces. Moreover, many defining properties of nanomedicines emerge at the population level, requiring multiscale predictive frameworks that connect single‐particle dynamics, ensemble behavior, and biological responses [258]. In parallel, although AI‐assisted pharmacokinetic models have advanced, their broader application to nanomedicine remains limited by the scarcity of high‐quality, standardized datasets [259, 260]. Further methodological advances will be required before computational tools can reliably translate in vitro descriptors into predictions of in vivo performance.

For supramolecular self‐assembled degradation platforms in particular, dedicated AI‐assisted predictive frameworks remain largely undeveloped, partly because their design logic differs from that of conventional nanomedicines. Reversible noncovalent interactions can make the emergent structures and functions of these assemblies strongly path‐dependent [261]. Static structure prediction and equilibrium‐based parameterization may therefore be insufficient to fully capture this path‐dependent complexity. Future predictive frameworks will need to integrate the physicochemical properties of the self‐assembled system, ligand‐receptor engagement, cellular uptake, endosomal escape, and pathway‐specific intracellular trafficking. They should also account for protein‐corona formation, interindividual variability, and disease heterogeneity. The broader nanomedicine field offers useful starting points, including design variables and datasets related to ligand density, surface properties, endosomal escape, and in vivo fate [23, 182, 262, 263, 264, 265]. Adapting these insights to supramolecular degraders will require dedicated datasets, mechanistic descriptors, and multiscale simulation and could reduce reliance on empirical optimization.

Progress toward this goal will require a clearer understanding of how supramolecular assembly mechanisms and dynamic behavior shape in vivo fate, supported by close collaboration across chemistry, biology, materials science, pharmaceutics, and computational science. Appropriately developed computational tools may then support more rational design and evaluation of supramolecular degrader systems.

## 7 Conclusion

Supramolecular degraders bring the organizing principles of supramolecular chemistry into targeted protein degradation (TPD), extending assembly beyond formulation to the control of degrader architecture, delivery, functional integration, and intracellular assembly or activation. The studies discussed in this review show that noncovalent modular decoupling, single‐component self‐assembly, multicomponent co‐assembly, and intracellular in situ assembly address distinct and complementary design challenges spanning modular construction, in vivo delivery, functional integration, and local processing. Nevertheless, the field remains at an early stage, and its further development will require quantitative and generalizable design rules that link molecular structure and assembly dynamics to biological fate and degradation outcomes. Translation will further depend on rigorous assessment of biofluid stability, systemic biodistribution, intracellular trafficking, pathway selectivity, indication selection, and long‐term safety, together with the development of experimentally grounded predictive models. Addressing these priorities could move supramolecular degraders beyond diverse proof‐of‐concept systems toward rationally designed TPD platforms with greater translational credibility.

## Author Contributions

K.L. led the manuscript drafting, literature synthesis, and revision coordination. X.Y. and Y.Z. performed the literature survey, organized the reference database, and categorized supramolecular degrader systems. R.Y. and S.X. contributed to figure preparation and graphical summary design. Q.G., J.G., P.W., and Y.C. co‐conceived the review framework, provided scientific guidance, and critically revised the manuscript. Q.G. guided the in vivo pharmacology, toxicology, and safety assessment sections. J.G. guided the mechanistic sections on proteasomal, endosomal–lysosomal, and autophagy–lysosomal degradation. P.W. developed the conceptual framework for the four modes of supramolecular involvement. Y.C. guided the sections on degrader design, structure–activity relationships, and preclinical development. All authors read and approved the final manuscript.

## Conflicts of Interest

The authors declare no conflicts of interest.

## Supporting information




**Supporting File**: advs77305‐sup‐0001‐SuppMat.docx.

## Data Availability

Data sharing not applicable to this article as no datasets were generated or analysed during the current study.
