# Supplementary material for: Supramolecular Degraders: An Emerging Paradigm in Targeted Protein Degradation
Source: Adv Sci (Weinh). 2026 Sep 13;13(58):e77305. doi: 10.1002/advs.77305 (PMC13572836; doi:10.1002/advs.77305)
Supplement: Supplementary file 1 [file ADVS-13-e77305-s001.docx]

**Supporting Information**

**Supramolecular Degraders: An Emerging Paradigm in Targeted Protein Degradation**

*Kongjun Liu^1†^, Xue Yuan^1†^, Yan Zhou^1†^, Rui Yuan^1^, Shaocheng Xie^1^, Yong Chen*^1^, Peiyi Wang^2^*, Jianmei Gao^1^*, Qihai Gong^1^**

^1^Key Laboratory of Basic Pharmacology of Ministry of Education and Joint International Research Laboratory of Ethnomedicine of Ministry of Education, Zunyi Medical University, Zunyi, 563000, China.

^2^State Key Laboratory of Green Pesticide, Key Laboratory of Green Pesticide and Agricultural Bioengineering, Ministry of Education, Center for Research and Development of Fine Chemicals of Guizhou University, Guiyang, 550025, China.

^*^Corresponding authors: [gqh@zmu.edu.cn](mailto:gqh@zmu.edu.cn) (Qihai Gong); [gaojianmei@zmu.edu.cn](mailto:gaojianmei@zmu.edu.cn) (Jianmei Gao); [pywang@gzu.edu.cn](mailto:pywang@gzu.edu.cn) (Peiyi Wang); [18702896006@163.com](mailto:18702896006@163.com) (Yong Chen).

^†^ These authors contributed equally to this review.

**Table S1.** Comparison of major targeted protein degradation modalities.

| **Strategy / class** | **Representative modalities or examples** | **Main route / target scope** | **Major strengths** | **Major limitations / open challenges** |
| --- | --- | --- | --- | --- |
| PROTACs | Vepdegestrant (ARV-471; Veppanu), the first FDA-approved PROTAC; other bifunctional E3-recruiting chimeras | UPS-based induced proximity between a POI and an E3 ligase; mainly cytosolic or nuclear intracellular proteins with ligandable binding sites | Most clinically advanced bifunctional TPD class; modular POI-ligand/linker/E3-ligand design enables target and E3 selection; event-driven degradation can remove scaffolding functions as well as catalytic activity | Productive degradation is highly ternary-complex-, ubiquitination- and cell-context-dependent; high molecular weight/polar surface area often compromise permeability and oral PK; excess degrader can cause a hook effect |
| MGDs | Thalidomide, lenalidomide, and pomalidomide analogues; other monovalent neomorphic E3 ligase glues | UPS-based monovalent molecules that stabilize or create E3-substrate interfaces; intracellular targets present a compatible glueable surface/degron | Compact architecture without a bifunctional linker or separate POI ligand; can redirect E3 ligases to targets that may lack classical inhibitor-like binding pockets | Discovery remains less programmable than PROTAC design; neosubstrate spectra and off-target degradation can be difficult to predict; dependence on a limited set of E3s/glueable interfaces constrains generalization; systemic exposure requires careful control |
| Endocytosis-based lysosome-directed degraders | LYTACs using CI-M6PR/M6Pn or ASGPR/GalNAc; AbTACs recruiting membrane E3 ligases such as RNF43 | Endosome-lysosome route; extracellular proteins and membrane proteins with accessible extracellular domains; tissue scope depends on the chosen internalizing receptor | Extends TPD beyond the cytosol and nucleus; can exploit antibody/aptamer specificity, lysosome-trafficking receptors and receptor recycling to clear soluble or cell-surface disease proteins | LYTAC performance depends on receptor expression, recycling and ligand-conjugation homogeneity that shape uptake and PK; AbTACs are large biologics and remain constrained by membrane-E3 availability and still-evolving mechanistic understanding; trafficking may favor recycling rather than degradation in some contexts |
| Autophagy-based lysosome-directed degraders | AUTACs, ATTECs, AUTOTACs, and related CMA-tethering designs | Macroautophagy or chaperone-mediated lysosomal degradation; intracellular proteins, aggregation-prone species, organelles or lipid droplets depending on the tag/receptor design | Can address bulky, aggregated or organellar substrates that are poorly matched to proteasomal unfolding; couples cargo sequestration with lysosomal digestion and can complement UPS-based degraders | Limitations are pathway-specific: AUTACs rely on S-guanylation/K63-ubiquitin logic, ATTECs require reliable LC3-target engagement, AUTOTAC/p62 approaches must avoid broad autophagy perturbation, and CMA designs require motif/chaperone compatibility; delivery, autophagic-flux capacity and cargo selectivity remain under-defined in vivo |

Abbreviations: TPD, targeted protein degradation; UPS, ubiquitin-proteasome system; PROTAC, proteolysis-targeting chimera; MGD, molecular glue degrader; LYTAC, lysosome-targeting chimera; AbTAC, antibody-based targeted chimera; AUTAC, autophagy-targeting chimera; ATTEC, autophagosome-tethering compound; CMA, chaperone-mediated autophagy.

**Table S2.** Summary of representative supramolecular systems used in TPD.

| **Platform name** | **Degradation mechanism** | **Degraded protein** | **Protein localization** |
| --- | --- | --- | --- |
| SupTACs^[1]^ | UPS | BRD4,  EGFR,  ACSL4,  MAPKs | Intracellular/Membrane |
| HGTACs^[2]^ | Endosome-lysosome | NS-650,  EGFR,  HER2 | Extracellular/Transmembrane |
| PLA-based SM-PROTAC^[3]^ | UPS | BRD4,  ERα,  CDK4 | Intracellular |
| NRs^[4]^ | Autophagy-lysosome | Mutant p53 (p53-S241F/R175H/R248W/R280K) | Intracellular |
| LipoSM-PROTAC^[5]^ | UPS | ERα | Intracellular |
| LipoSM-PROTAC^[6]^ | UPS | MEK1/2,  ALK | Intracellular |
| LIPOTAC^[7]^ | UPS | HK-2 | Intracellular |
| GM-protac^[8]^ | UPS + autophagy-lysosome | EGFR | Transmembrane |
| SM-PROTAC^[9]^ | UPS | ERα,  CDK4/6,  AR,  MEK1/2,  BRD2/4,  BCR-ABL,  EGFR | Intracellular/Transmembrane |
| Multi-SM-PROTAC^[10]^ | UPS | ERα,  CDK4,  AR,  BRD2/4 | Intracellular |
| Supra-PROTACs^[11]^ | UPS | Bcl-xL,  HER2 | Intracellular/Transmembrane |
| Supra-LYTAC^[12]^ | Endosome-lysosome | Streptavidin,  PD-L1 | Extracellular/Membrane |
| LYTACAs^[13]^ | Endosome-lysosome | IL-17A,  PD-L1 | Extracellular/Membrane |
| SM-CMAD^[14]^ | CMA | ERα,  AR,  MEK1/2,  BCR-ABL | Intracellular/Membrane |
| DbTACs^[15]^ | UPS | CDK9,  CDK6,  ERG,  HPK1 | Intracellular |
| DTACs^[16]^ | UPS | Cyclin D1–CDK4/6 complex | Intracellular |
| OligoPROTACs^[17]^ | UPS | BRD4,  MYC | Intracellular |
| miRiaTAC^[18]^ | UPS | BRD4,  CDK6,  CDK4 | Intracellular |
| AS-TD2-PRO^[19]^ | UPS | STAT3,  FOXM1 | Intracellular |
| DNA Nanoinhibitor^[20]^ | Endosome-lysosome | c-Met,  HER2 | Membrane |
| LYTAC Plus hydrogel^[21]^ | Endosome-lysosome | VEGFR-2;  ANG-2 (gene silencing) | Membrane |
| PT-NPs^[22]^ | Endosome-lysosome + UPS | PD-L1 | Transmembrane |
| NanoTACs^[23]^ | Endosome-lysosome + UPS | EGFR (WT and L858R/T790M) | Transmembrane |
| Nano-LYTACs^[24]^ | Endosome-lysosome | CD24 | Membrane (GPI-anchored) |
| Z_EGFR:1907_-MS28 ADCN^[25]^ | UPS | Cyclin D1 | Intracellular |
| Z_HER2:342_-MZ1 APCN^[26]^ | UPS | BRD2,  BRD3,  BRD4 | Intracellular |
| MPRO^[27]^ | UPS | EGFR | Intracellular |
| RCNprotac^[28]^ | UPS | BRD4 | Intracellular |
| Cle-NP (nano-mGlu)^[29]^ | UPS | BCR-ABL,  ABL | Intracellular |
| NFTP^[30]^ | UPS | FOXM1 | Intracellular |
| NapYp-ARV^[31]^ | UPS | BRD4 | Intracellular |
| pNano-PROTAC_GPX4_^[32]^ | UPS | GPX4 | Intracellular |
| SPN_pro_^[33]^ | UPS | IDO | Intracellular |
| SPN_COX_^[34]^ | UPS | COX-1,  COX-2 | Intracellular |
| NAP^[35]^ | UPS | BRD2,  BRD3,  BRD4 | Intracellular |
| NPRO^[36]^ | UPS | SHP2 | Intracellular |
| LPN^[37]^ | UPS | IDO | Intracellular |
| NanoTAC^[38]^ | UPS | HK-2 | Intracellular |
| Fi-dBET6 NPs / Fi-MS432 NPs^[39]^ | UPS | BRD4,  MEK1/2 | Intracellular |
| BHP NPs^[40]^ | UPS | BRD4 | Intracellular |
| PPET₃N₂/P9TC Polyplexes^[41]^ | UPS | ERα | Intracellular |
| TPD-NPs^[42]^ | Autophagy-lysosome | EGFR,  HER2,  PD-1,  PD-L1,  ACE2,  CD13,  etc. | Transmembrane |
| NaC4A-PROTACs^[43]^ | UPS | BRD4 | Intracellular |
| DdLD NPs^[44]^ | UPS | BRD4 | Intracellular |
| Ce6-PROTAC NPs^[45]^ | UPS | CDK4, CDK6 | Intracellular |
| SDNpro^[46]^ | UPS | BRD4 | Intracellular |
| GM NPs^[47]^ | Autophagy-lysosome | IDO | Intracellular |
| PRO-P^[48]^ | UPS | HMGCR | Intracellular |
| POLY-PROTAC NPs (PGD7 / N₃@PGDA7)^[49]^ | UPS | BRD4 | Intracellular |
| LND-MZ1^[50]^ | UPS | BRD4 | Intracellular |
| RPG7 NPs / RPG7@DOX^[51]^ | UPS | BRD4,  CDK9 | Intracellular |
| NP_Ce6+PRO_^[52]^ | UPS | BRD4 | Intracellular |
| PPT7 NPs^[53]^ | UPS | BRD4 | Intracellular |

**Table S3.** Representative carrier-assembled nanomedicine formulations for degrader delivery.

| **Platform name** | **Carrier type / carrier-forming components** | **Loaded degrader** | **Degradation profile** | **Responsive or release mechanism** | **Added function(s)** | **Boundary rationale** |
| --- | --- | --- | --- | --- | --- | --- |
| L@NBMZ^[54]^ | Liposomal carrier; DPPC, DSPE-mPEG2000, NBSEt | MZ1 | UPS; BRD4; Intracellular | Light-triggered NBSEt activation | PDT, photo-driven pyroptosis, innate immune activation | MZ1 as liposomal cargo; lipid carrier governs assembly |
| HFn@Thal^[55]^ | Protein nanocage; HFn protein subunits | Thal-SNS-032 | UPS; CDK9; Intracellular | pH-triggered HFn disassembly/reassembly; acidic release | TfR1-mediated targeting, pH-responsive release | Thal-SNS-032 loaded in HFn; HFn subunits govern architecture |
| ARV-MBs^[56]^ | Phospholipid microbubble; DPPC, DSPE-mPEG2000, C3F8 | ARV-825 | UPS; BRD4; Intracellular | Ultrasound-triggered microbubble destruction | UTMD, sonoporation-assisted uptake | ARV-825 as microbubble cargo; microbubble carrier governs release |
| ARV-DOX/cRGD-P^[57]^ | Polymeric nanoparticle; MPEG-PCL, cRGD-PEG-PCL, DOX | ARV-825 | UPS; BRD4; Intracellular | Not specifically reported | αvβ3 targeting, DOX chemotherapy | ARV-825/DOX co-loaded; polymer carrier governs assembly |
| dBET6@nano-CXCL9^[58]^ | MSPM nanochaperone; PEG-b-PCL, COOH-PEG-b-PCL, PCL-b-PAE, CXCL9 | dBET6 | UPS; BRD4; Intracellular | pH 6.5/CXCL9 release; pH 5.0/dBET6 release | CXCL9 immune recruitment, ICD, PD-L1 downregulation | dBET6 in micellar core; PCL/PAE carrier governs staged release |
| YM@VBM^[59]^ | Coordination/hybrid carrier; NH_4_VO_3_, baicalin, mPEG-NH_2_ | YM281 | UPS; EZH2; Intracellular | pH-dependent YM281 release; acidic POD-like activity; 808 nm/PTT | CDT, GSH depletion, PTT, ICD, immune activation | YM281 as cargo; V–baicalein carrier governs nanozyme/PTT functions |
| ARV@PEG-ICG^[60]^ | Amphiphilic phototherapeutic carrier; PEG-ICG-based carrier | ARV-825 | UPS; BRD4; Intracellular | pH 5.5/ARV-825 release; 808 nm/PTT/PDT | PTT, PDT, ICD, DC/T-cell activation | ARV-825 as cargo; PEG-ICG carrier governs response and phototherapy |
| SPN_FeP_^[61]^ | Semiconducting polymer nanoparticle; PFODBT, ferrocene, DSPE-TK-PEG | NAMPT PROTAC | UPS; NAMPT; Intracellular | H_2_O_2_/US-amplified PROTAC release | SDT, ferroptosis, ICD, MDSC suppression | PROTAC as cargo; PFODBT/ferrocene carrier governs activation |
| RPB7H^[62]^ | hybrid nanoparticle; CRGDK-PEG-b-PLGA/HfO_2_ nanosensitizer | BPA771 | UPS; BRD4; Intracellular | RT-induced H_2_O_2_; PBE cleavage | NRP-1 targeting, RT sensitization, DDR blockade | BPA771/HfO2 co-loaded; PLGA/HfO2 carrier governs targeting and RT response |

**Table S4.** Summary of representative intracellular in situ construction systems used to regulate TPD.

| **Platform name** | **Degradation mechanism** | **Degraded protein** | **Protein localization** |
| --- | --- | --- | --- |
| Nano-PROTACs^[63]^ | UPS | EGFR,  AR | Transmembrane/Intracellular |
| ROS-instructed supramolecular assembly platform^[64]^ | UPS | BRD4 | Intracellular |
| Psa-AR^[65]^ | UPS | AR,  AR-V7,  HSP90 | Intracellular |
| PACES^[66]^ | UPS | H2B,  Cofilin-2 | Intracellular |
| Nano-CLIPTACs^[67]^ | UPS | EML4-ALK | Intracellular |
| BT-PROTAC^[68]^ | UPS | BRD4 | Intracellular |
| BTz-enabled ROS-activated tetrazine ligation^[69]^ | UPS | BRD4 | Intracellular |
| ABC-PROTAC^[70]^ | UPS | BRD4,  PARP1  NF-κB | Intracellular |
| Split-Deliver-Click^[71]^ | UPS | BRD4 | Intracellular |
| Ligation to scavenging system^[72]^ | UPS-mediated degradation termination | BRD2/3/4 and PARP1 degradation was terminated | Intracellular |

Note: The ligation-to-scavenging system does not itself induce protein degradation. It terminates ongoing UPS-mediated degradation by covalently sequestering intracellular Tz-PROTACs on PAMAM-G5-TCO.

**Reference**

1. J. Liu, T. Ma, R. Yao, L. Li, Q. Zheng, and M. Wang, “Multimodal Supramolecular Targeting Chimeras Enable Spatiotemporally Resolved Protein Degradation in Vivo,” *Cell* *189*, no. 4 (2026): 1108-1123.e20.

2. X. Chen, T. Wu, Y. Chen, et al., “Supramolecular Host–Guest Assemblies for Tunable and Modular Lysosome-Targeting Protein Degradation,” *Angewandte Chemie* *137*, no. 33 (2025): e202506618.

3. M. Zhan, H. Chen, M. He, et al., “Highly Efficient and Long-Acting Split-and-Mix Proteolysis Targeting Chimera Based on Self-Assembled Polylactic Acid,” *Nature Communications* *16*, no. 1 (2025): 10555.

4. X. Huang, Z. Cao, J. Qian, et al., “Nanoreceptors Promote Mutant P53 Protein Degradation by Mimicking Selective Autophagy Receptors,” *Nature Nanotechnology* *19*, no. 4 (2024): 545–553.

5. C. Song, Z. Jiao, Z. Hou, et al., “Selective Protein of Interest Degradation through the Split-and-Mix Liposome Proteolysis Targeting Chimera Approach,” *Journal of the American Chemical Society* *145*, no. 40 (2023): 21860–21870.

6. C. Song, Z. Jiao, Z. Hou, et al., “Versatile Split-and-Mix Liposome PROTAC Platform for Efficient Degradation of Target Protein *In Vivo*,” *JACS Au* *4*, no. 8 (2024): 2915–2924.

7. L. Gong, S. Li, J. Sun, et al., “Targeted Degradation of Hexokinase 2 by a Novel Engineered Bispecific Chimeric Liposome-Based Nano-PROTACs for Enhancing Tumor Chemotherapy,” *Biomaterials* *326* (2026): 123651.

8. D. Wang, Y. Liu, Y. Chen, et al., “EGFR Targeted Liposomal PROTAC Assisted with Epigenetic Regulation as an Efficient Strategy for Osimertinib-Resistant Lung Cancer Therapy,” *Advanced Science* *12*, no. 43 (2025): e10197.

9. F. Yang, Q. Luo, Y. Wang, et al., “Targeted Biomolecule Regulation Platform: A Split-and-Mix PROTAC Approach,” *Journal of the American Chemical Society* *145*, no. 14 (2023): 7879–7887.

10. Y. Wang, Q. Luo, Y. Xing, et al., “A Multi-Split-and-Mix Platform for a Variety of Targeted Protein Degradation,” *ACS Chemical Biology* *20*, no. 9 (2025): 2287–2297.

11. N. Chen, Z. Zhang, X. Liu, et al., “Sulfatase-Induced In Situ Formulation of Antineoplastic Supra-PROTACs,” *Journal of the American Chemical Society* *146*, no. 15 (2024): 10753–10766.

12. D. Kim, G. Yang, C. Lim, et al., “Cancer Specific CAIX-Targeting Supramolecular Lysosome-Targeting Chimeras (Supra-LYTAC) for Targeted Protein Degradation,” *Advanced Science* *12*, no. 26 (2025): 2503134.

13. Q. Wang, X. Yang, R. Yuan, et al., “A Co-Assembly Platform Engaging Macrophage Scavenger Receptor a for Lysosome-Targeting Protein Degradation,” *Nature Communications* *15*, no. 1 (2024): 1663.

14. J. Wang, Y. Wang, F. Yang, et al., “A Novel Lysosome Targeting Chimera for Targeted Protein Degradation via Split-and-Mix Strategy,” *ACS Chemical Biology* *19*, no. 5 (2024): 1161–1168.

15. L. Zhou, B. Yu, M. Gao, et al., “DNA Framework-Engineered Chimeras Platform Enables Selectively Targeted Protein Degradation,” *Nature Communications* *14*, no. 1 (2023): 4510.

16. R. Zheng, A. Prasad, D. Satyabola, et al., “DNA-Templated Spatially Controlled Proteolysis Targeting Chimera for Cyclin D1–CDK4/6 Complex Protein Degradation,” *Journal of the American Chemical Society* *147*, no. 33 (2025): 29742–29755.

17. D. Kashyap, S. Haider, T. A. Milne, and M. J. Booth, “DNA-Programmable Protein Degradation: Dynamic Control of Proteolysis-Targeting Chimera Activity via DNA Hybridization and Strand Displacement,” *JACS Au* *5*, no. 8 (2025): 3799–3807.

18. J. Zhan, X. Li, Z. Feng, et al., “MicroRNA-Triggered Programmable DNA-Encoded Pre-PROTACs for Cell-Selective and Controlled Protein Degradation,” *Angewandte Chemie International Edition* *64*, no. 3 (2025): e202415323.

19. S. Li, T. Zeng, Z. Wu, et al., “DNA Tetrahedron-Driven Multivalent Proteolysis-Targeting Chimeras: Enhancing Protein Degradation Efficiency and Tumor Targeting,” *Journal of the American Chemical Society* *147*, no. 2 (2025): 2168–2181.

20. J. Tang, C. Qi, X. Bai, et al., “Cell Membrane-Anchored DNA Nanoinhibitor for Inhibition of Receptor Tyrosine Kinase Signaling Pathways via Steric Hindrance and Lysosome-Induced Protein Degradation,” *ACS Pharmacology & Translational Science* *7*, no. 1 (2024): 110–119.

21. Y. Huang, X. Zhou, Y. Zhang, et al., “A Nucleic Acid-Based LYTAC plus Platform to Simultaneously Mediate Disease-Driven Protein Downregulation,” *Advanced Science* *11*, no. 13 (2024): 2306248.

22. Y. Moon, H. Cho, J. Kim, et al., “Self-Assembled Peptide-Derived Proteolysis-Targeting Chimera (PROTAC) Nanoparticles for Tumor-Targeted and Durable PD-L1 Degradation in Cancer Immunotherapy,” *Angewandte Chemie* *137*, no. 5 (2025): e202414146.

23. J. Jeong, H. Cho, Y. Moon, et al., “Effective Degradation of Wild-Type and Mutant EGFR Using Self-Assembling Peptide-Derived PROTAC Nanoparticles (NanoTACs) for Cancer Therapy,” *Advanced Materials* *38*, no. 9 (2026): e16974.

24. K. Wang, A. Yu, K. Liu, et al., “Nano-LYTACs for Degradation of Membrane Proteins and Inhibition of CD24/Siglec-10 Signaling Pathway,” *Advanced Science* *10*, no. 13 (2023): 2300288.

25. W. Gao, X. Xia, X. Yang, et al., “Amphiphilic Affibody-PROTAC Conjugate Self-Assembled Nanoagents for Targeted Cancer Therapy,” *Chemical Engineering Journal* *495* (2024): 153437.

26. Q. Li, X. Yang, M. Zhao, et al., “A Self-Assembled Affibody-PROTAC Conjugate Nanomedicine for Targeted Cancer Therapy,” *Nano Research* *17*, no. 11 (2024): 9954–9964.

27. J. Ma, L. Fang, Z. Sun, et al., “Folate-PEG-PROTAC Micelles for Enhancing Tumor-Specific Targeting Proteolysis In Vivo,” *Advanced Healthcare Materials* *13*, no. 20 (2024): 2400109.

28. M. Xu, Y. Yun, C. Li, et al., “Radiation Responsive PROTAC Nanoparticles for Tumor-Specific Proteolysis Enhanced Radiotherapy,” *Journal of Materials Chemistry B* *12*, no. 13 (2024): 3240–3248.

29. J. Sun, M. Gu, L. Peng, et al., “A Self-Assembled Nano-Molecular Glue (Nano-mGlu) Enables GSH/H_2_O_2_-Triggered Targeted Protein Degradation in Cancer Therapy,” *Journal of the American Chemical Society* *147*, no. 1 (2025): 372–383.

30. H. Zeng, Z. Fang, Y. Feng, T. Su, W. Miao, and Z. Wang, “Self-Assembled Peptide PROTAC Prodrugs Targeting FOXM1 for Cancer Therapy,” *Molecular Pharmaceutics* *22*, no. 6 (2025): 3286–3296.

31. Y. Yang, C. Yang, Z. Fu, et al., “Enzyme-Instructed Nanofibrous Assemblies Enable Targeted PROTAC Release for Cancer Therapy,” *Advanced Healthcare Materials* (2026): e05105.

32. N.-Y. Zhang, Z. Wen, M.-Z. Cai, et al., “De Novo Design of Hexamer-Linker Dual-Action Nano-PROTAC for Tumor-Specific Ferroptosis,” *Angewandte Chemie* *138*, no. 2 (2026): e09924.

33. C. Zhang, Z. Zeng, D. Cui, et al., “Semiconducting Polymer Nano-PROTACs for Activatable Photo-Immunometabolic Cancer Therapy,” *Nature Communications* *12*, no. 1 (2021): 2934.

34. C. Zhang, S. He, Z. Zeng, P. Cheng, and K. Pu, “Smart Nano-PROTACs Reprogram Tumor Microenvironment for Activatable Photo-Metabolic Cancer Immunotherapy,” *Angewandte Chemie International Edition* *61*, no. 8 (2022): e202114957.

35. W. Wang, C. Zhu, B. Zhang, Y. Feng, Y. Zhang, and J. Li, “Self-Assembled Nano-PROTAC Enables near-Infrared Photodynamic Proteolysis for Cancer Therapy,” *Journal of the American Chemical Society* *145*, no. 30 (2023): 16642–16649.

36. C. Zhang, M. Xu, S. He, J. Huang, C. Xu, and K. Pu, “Checkpoint Nano-PROTACs for Activatable Cancer Photo-Immunotherapy,” *Advanced Materials* *35*, no. 6 (2023): 2208553.

37. J. Choi, B. Park, J. Y. Park, et al., “Light-Triggered PROTAC Nanoassemblies for Photodynamic IDO Proteolysis in Cancer Immunotherapy,” *Advanced Materials* *36*, no. 38 (2024): 2405475.

38. B. Park, J. Choi, J.-H. Lee, et al., “Reprogramming of Cancer Metabolism via Photoresponsive Nano-PROTAC Enhances Pyroptosis-Mediated Immunotherapy,” *Signal Transduction and Targeted Therapy* *10*, no. 1 (2025): 310–325.

39. K. C. Vogt, M. Panagiotakopoulou, M. T. Manzari Honu, et al., “Tumor Microenvironment–Targeted PROTAC Nanoparticle Self-Assembly Broadly Predicted by Structural Descriptors,” *Science Advances* *11*, no. 49 (2025): eadu2292.

40. K. Zhang, Y. Ha, Y. Gan, et al., “Self-Assembled Dual-Targeting Nanoplatform for Synergistic Photodynamic and PROTAC-Mediated Metabolic Starvation in Triple-Negative Breast Cancer,” *Chemical Engineering Journal* *520* (2025): 165815.

41. Y. Sun, L. Jiang, Z. Zhang, N. Xu, Y. Jiang, and C. Tan, “Conjugated Polyelectrolyte/Single Strand DNA Hybrid Polyplexes for Efficient Nucleic Acid Delivery and Targeted Protein Degradation,” *ACS Appl. Mater. Interfaces* *16* (2024): 19987–19994.

42. Y. Liu, R. Liu, J. Dong, et al., “Targeted Protein Degradation via Cellular Trafficking of Nanoparticles,” *Nature nanotechnology* *20*, no. 2 (2025): 296–302.

43. J. Lin, W. Wang, A.-K. Ying, et al., “Hypoxia-Sensitive Macrocycle Inclusion Complexes for Targeted Protein Degradation,” *Journal of Controlled Release* *384* (2025): 113921.

44. L.-P. Zhao, R.-R. Zheng, X.-N. Rao, et al., “Chemotherapy-Enabled Colorectal Cancer Immunotherapy of Self-Delivery Nano-PROTACs by Inhibiting Tumor Glycolysis and Avoiding Adaptive Immune Resistance,” *Advanced Science* *11*, no. 15 (2024): 2309204.

45. T. Wang, Y. Zhang, K. Chen, et al., “CDK4/6 Nano-PROTAC Enhances Mitochondria-Dependent Photodynamic Therapy and Anti-Tumor Immunity,” *Nano Today* *50* (2023): 101890.

46. L.-P. Zhao, X.-N. Rao, R.-R. Zheng, et al., “Carrier-Free Nano-PROTACs to Amplify Photodynamic Therapy Induced DNA Damage through BRD4 Degradation,” *Nano Letters* *23*, no. 13 (2023): 6193–6201.

47. Y. Wang, L. Yang, C. Yan, et al., “Supramolecular Artificial Nano-AUTACs Enable Tumor-Specific Metabolism Protein Degradation for Synergistic Immunotherapy,” *Science Advances* *10*, no. 25 (2024): eadn8079.

48. T. Su, G. Li, Y. Guan, et al., “PROTAC-Mediated HMGCR Depletion Reprograms Lipid Metabolism in Breast Cancer to Potentiate Photoimmunotherapy via Ferroptosis,” *Advanced Science* *13* (2026): e202521525.

49. J. Gao, B. Hou, Q. Zhu, et al., “Engineered Bioorthogonal POLY-PROTAC Nanoparticles for Tumour-Specific Protein Degradation and Precise Cancer Therapy,” *Nature Communications* *13*, no. 1 (2022): 4318.

50. M. Pan, C. Yang, Z. Fu, et al., “Remodeling the Physicochemical and Pharmacokinetic Properties of PROTAC via Lipid Nanodisks for Cancer Therapy,” *Advanced Science* *12*, no. 36 (2025): e01384.

51. Z. Zou, L. Yang, H. Nie, et al., “Tumor-Targeted PROTAC Prodrug Nanoplatform Enables Precise Protein Degradation and Combination Cancer Therapy,” *Acta Pharmacologica Sinica* *45*, no. 8 (2024): 1740–1751.

52. Y. Liu, H. Wang, M. Ding, et al., “Ultrasound-Activated PROTAC Prodrugs Overcome Immunosuppression to Actuate Efficient Deep-Tissue Sono-Immunotherapy in Orthotopic Pancreatic Tumor Mouse Models,” *Nano Letters* *24*, no. 28 (2024): 8741–8751.

53. X. Jiang, Y. Lai, W. Xia, et al., “Self-Oxygenating PROTAC Microneedle for Spatiotemporally-Confined Protein Degradation and Enhanced Glioblastoma Therapy,” *Advanced Materials* *37*, no. 13 (2025): 2411869.

54. D. Huang, Y. Zou, H. Huang, et al., “A PROTAC Augmenter for Photo-Driven Pyroptosis in Breast Cancer,” *Advanced Materials* *36*, no. 21 (2024): 2313460.

55. H. Xu, Y. Fan, J. Jin, et al., “Ferritin Nanocages Facilitate PROTAC Delivery for Enhanced Targeted Therapy in Triple-Negative Breast Cancer,” *Chemical Engineering Journal* *518* (2025): 164697.

56. H. He, F. Li, R. Tang, et al., “Ultrasound Controllable Release of Proteolysis Targeting Chimeras for Triple-Negative Breast Cancer Treatment,” *Biomaterials Research* *28* (2024): 0064.

57. Y. He, Y. Ju, Y. Hu, et al., “Brd4 Proteolysis-Targeting Chimera Nanoparticles Sensitized Colorectal Cancer Chemotherapy,” *Journal of Controlled Release* *354* (2023): 155–166.

58. J. Chen, F. Ma, Y. Zhang, et al., “Synergizing CXCL9 with BRD4-PROTAC Using Nanochaperone Boosts Robust T Cell-Dependent Antitumor Immune Responses for Cancer Immunotherapy,” *Adv. Funct. Mater.* *34* (2024): 2314203.

59. Z. Chen, Y. Tai, C. Deng, et al., “Innovative Sarcoma Therapy Using Multifaceted Nano-PROTAC-Induced EZH2 Degradation and Immunity Enhancement,” *Biomaterials* *321* (2025): 123344.

60. Z. Li, G. Ren, X. Wang, et al., “Tumor Microenvironment Responsive Nano-PROTAC for BRD4 Degradation Enhanced Cancer Photo-Immunotherapy,” *Biomaterials* *322* (2025): 123387.

61. F. Wang, G. Dong, M. Ding, N. Yu, C. Sheng, and J. Li, “Dual-Programmable Semiconducting Polymer NanoPROTACs for Deep-Tissue Sonodynamic-Ferroptosis Activatable Immunotherapy,” *Small* *20*, no. 8 (2024): 2306378.

62. S. Zhang, Y. Lai, J. Pan, et al., “PROTAC Prodrug-Integrated Nanosensitizer for Potentiating Radiation Therapy of Cancer,” *Advanced Materials* *36*, no. 23 (2024): 2314132.

63. N.-Y. Zhang, D.-Y. Hou, X.-J. Hu, et al., “Nano Proteolysis Targeting Chimeras (PROTACs) with Anti-Hook Effect for Tumor Therapy,” *Angewandte Chemie International Edition* *62*, no. 37 (2023): e202308049.

64. Q. Yao, Z. Wu, J. Li, et al., “Reactive Oxygen Species-Instructed Supramolecular Assemblies Enable Bioorthogonally Activatable Protein Degradation for Pancreatic Cancer,” *Journal of the American Chemical Society* *147*, no. 21 (2025): 18208–18218.

65. F.-Y. Yang, N.-Y. Zhang, Y. Yang, et al., “In Vivo Self-Assembled Nano-PROTAC for the Dual Degradation of AR and HSP90 to Overcome Castration-Resistant Prostate Cancer Resistance,” *Signal Transduction and Targeted Therapy* *10*, no. 1 (2025): 346.

66. X. Hu, Q. Yao, X. Wu, et al., “In Situ Construction of Intracellular Supramolecular Assemblies as an Alternative Strategy for Protein Degradation,” *Nature Communications* *16*, no. 1 (2025): 5974.

67. S. Xie, J. Zhu, Y. Peng, et al., “*In Vivo* Self-Assembly of PROTACs by Bioorthogonal Chemistry for Precision Cancer Therapy,” *Angewandte Chemie* *137*, no. 11 (2025): e202421713.

68. T. Bi, P. Liang, Y. Zhou, et al., “Rational Design of Bioorthogonally Activatable PROTAC for Tumor-Targeted Protein Degradation,” *Journal of Medicinal Chemistry* *66*, no. 21 (2023): 14843–14852.

69. D. Ming, J. Zhang, B. Mu, et al., “Reactive Oxygen Species-Activated Bioorthogonal Chemistry in Living Systems Enabled by Boronate-Caged Dihydrotetrazines,” *Nature Communications* *17* (2026): 2568.

70. D. Ouyang, R. Yang, Y. Yao, et al., “In Situ PROTAC Synthesis Enabled by Pathologically Activated Bioorthogonal Catalysis for Precision Cancer Therapy,” *Journal of the American Chemical Society* *147*, no. 43 (2025): 39987–39998.

71. H. Dong, C. Chu, I. Ullah, Q. Xu, Z. Pan, and Y. Yuan, “Split-Deliver-Click: Tumor-Specific Protein Degradation via ‘AND’ Logic-Gated In-Cell Bioorthogonal Clicking of PROTACs,” *Angewandte Chemie International Edition* (2026): e20774.

72. Y. Jin, J. Fan, R. Wang, et al., “Ligation to Scavenging Strategy Enables On-Demand Termination of Targeted Protein Degradation,” *Journal of the American Chemical Society* *145*, no. 13 (2023): 7218–7229.
